# Enhanced SNP-sensing using DNA-templated reactions through confined hybridization of minimal substrates (CHOMS)†

**DOI:** 10.1039/d0sc00741b

**Published:** 2020-03-24

**Authors:** Ki Tae Kim, Nicolas Winssinger

**Affiliations:** Department of Organic Chemistry, NCCR Chemical Biology, Faculty of Science, University of Geneva 30 quai Ernest Ansermet 1211 Geneva Switzerland Nicolas.Winssinger@unige.ch

## Abstract

DNA or RNA templated reactions are attractive for nucleic acid sensing and imaging. As for any hybridization-based sensing, there is a tradeoff between sensitivity (detection threshold) and resolution (single nucleotide discrimination). Longer probes afford better sensitivity but compromise single nucleotide resolution due to the small thermodynamic penalty of a single mismatch. Herein we report a design that overcomes this tradeoff. The reaction is leveraged on the hybridization of a minimal substrate (covering 4 nucleotides) which is confined by two guide DNAs functionalized respectively with a ruthenium photocatalyst. The use of a catalytic reaction is essential to bypass the exchange of guide DNAs while achieving signal amplification through substrate turnover. The guide DNAs restrain the reaction to a unique site and enhance the hybridization of short substrates by providing two π-stacking interactions. The reaction was shown to enable the detection of SNPs and SNVs down to 50 pM with a discrimination factor ranging from 24 to 309 (median 82, 27 examples from 3 oncogenes). The clinical diagnostic potential of the technology was demonstrated with the analysis of RAS amplicons obtained directly from cell culture.

## Abstract 2

Confined hybridization of a minimal substrate: striking the right balance between specificity and turnover

## Graphical abstract

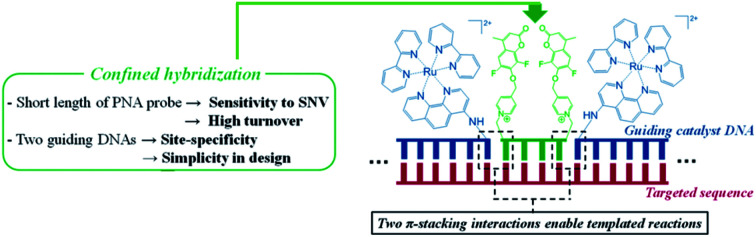

## Introduction

There are over fifty million single nucleotide polymorphisms (SNPs) in humans,^1^ defined as a variation of a single nucleotide at a given position in the genome. While the vast majority are inconsequential, a number of SNPs have been correlated directly with specific phenotypes or diseases^2,3^ (such as sickle-cell anaemia^4^). These SNPs also provide important ancestry information that is fundamental in forensic and anthropological sciences.^5^ An individual's SNPs can be used to infer their geographic origins, down to a hundred kilometers in some cases.^6^ SNPs also affect susceptibility to cancer.^7^ Furthermore, a single nucleotide variation (SNV) may occur from point mutation arising in the development of tumours. Notably, resistance to targeted therapy with imatinib, an inhibitor of *BCR-ABL* used in the treatment of chronic myeloid leukaemia, can arise from a C to T SNV resulting in T315I mutation;^8^ specific therapeutics have been developed for this mutation.^9^ Another example is a mutation in *KRAS* (G12C)^10^ which is the target of the first KRAS-targeting drug in clinical development.^11^ Accordingly, technologies to analyse SNPs and SNVs are critical in medical diagnosis, personalized medicine and many other scientific disciplines. Currently, allele-specific PCR is the default technology. This technology makes use of a primer covering the SNP of interest, thus resulting in different rates of amplification for a matched *vs.* mismatched primer with 40- to 100-fold discrimination.^12,13^ The need to achieve more cost-effective, faster analysis and higher single nucleotide resolution continues to drive technological developments.^14^ Recent examples include amendments to existing technologies such as molecular beacons,^15,16^ melting analysis,^17,18^ environmentally sensitive fluorescent nucleobases,^19–24^ and strand displacement probes^25,26^ or new technologies such as polymerase-amplified release of ATP (POLARA)^27^ or graphene-based biosensors for real-time kinetic monitoring of hybridization.^28^ The analysis of SNVs requires technologies with the highest nucleotide resolution to ascertain the polymorphism or variation. This challenge can be exacerbated in the case of tumour biopsies where samples may be heterogeneous due to the polyclonal nature of the tumour and contaminated with healthy tissue.

**Fig. 1:**
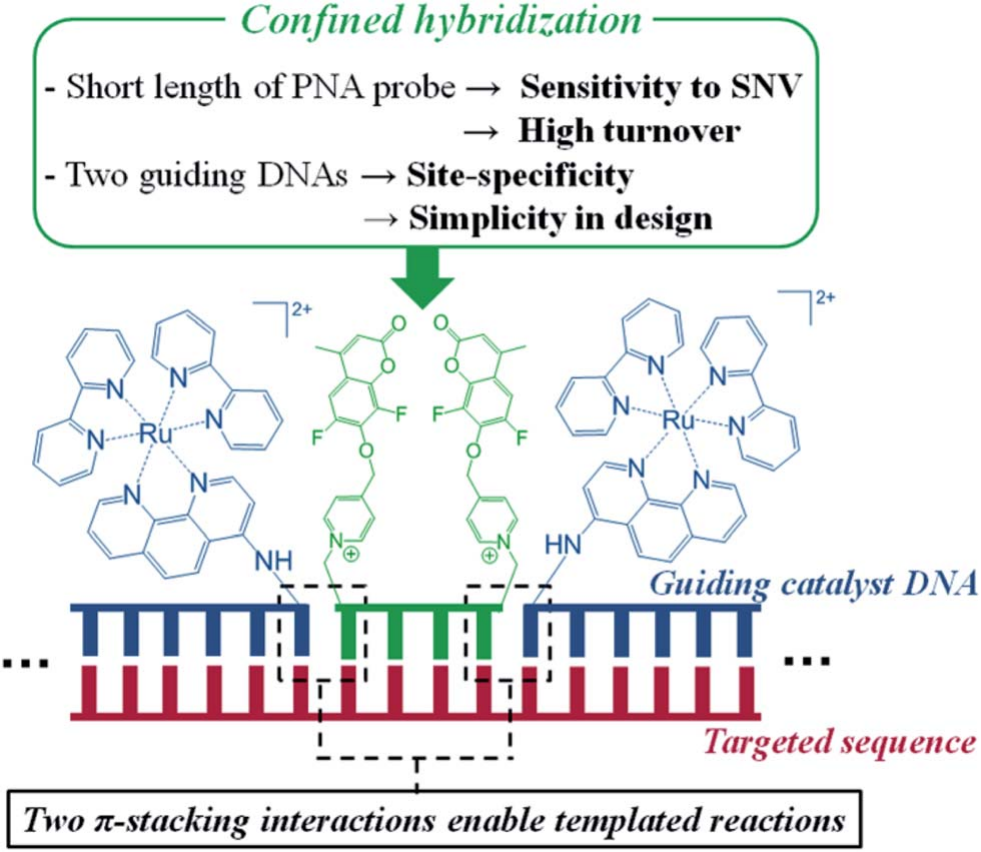
Graphical representation of the reaction with confined hybridization of a minimal substrate (4-mer) – CHOMS.

DNA- or RNA-templated reactions, wherein hybridization of probe–reagent conjugates to a targeted oligonucleotide promotes a reaction, have proven to be a fast, simple and robust approach for nucleic acid sensing.^29–32^ This technology has been applied to SNP detection.^33–39^ However, as for any binary probe detection,^40^ there is a delicate trade-off between sensitivity and single nucleotide resolution; the longer the probes, the better the detection threshold but the lower the resolution. In templated reactions, this must be further balanced with amplification; the longer the probe, the slower the template turn-over (amplification). The ideal case is a fast templated reaction capable of providing high turnovers which requires short probes. In practice, reactions are typically performed with probes of 8–15 nt to achieve a detection threshold below the nanomolar range of analytes. For a unique sequence in the genome, strings of >18 nt should ideally be considered.^41^ A further complication in templated reactions is that many reactions have low sensitivity to the distance between the two probes,^42^ and a small gap between the reaction sites has in fact been found to moderately enhance the rate in some cases.^34,43,44^ Thus, the analysis of the uniqueness of a target sequence in a templated reaction must consider different permutations of gaps between the two probes. Recently, we discovered that templated reactions with short substrates are enhanced when performed at a sticky ends, allowing reactions with just 4-mer PNA substrates, provided that the sticky-end hybridization involved purine–purine π-stacking.^45^ Herein, we extend the utility of this finding and report a system that is exceptionally selective to SNPs/SNVs by virtue of a confined-hybridization of a minimal substrate between two guide DNAs (Fig. 1). The reaction makes use of a ruthenium-photocatalyzed immolation of a pyridinium linker to uncage a pro-fluorophore.^46^ The choice of this reaction was based on its fast kinetics, its biorthogonality^47^ and the fact that the guide DNAs do not need to exchange on the template in order to achieve amplification. While a templated reaction with sandwich probes has been reported,^37^ it explored the benefit of a double ligation reaction to suppress the background signal arising from the hydrolysis of a labile quencher. Since two reactions are required to remove the quenchers, the signal to noise ratio of detection was improved. However, this system is not suitable for catalytic amplification and the benefits of π-stacking to enhance single nucleotide resolution with short substrates.

## Results and discussion

**Fig. 2:**
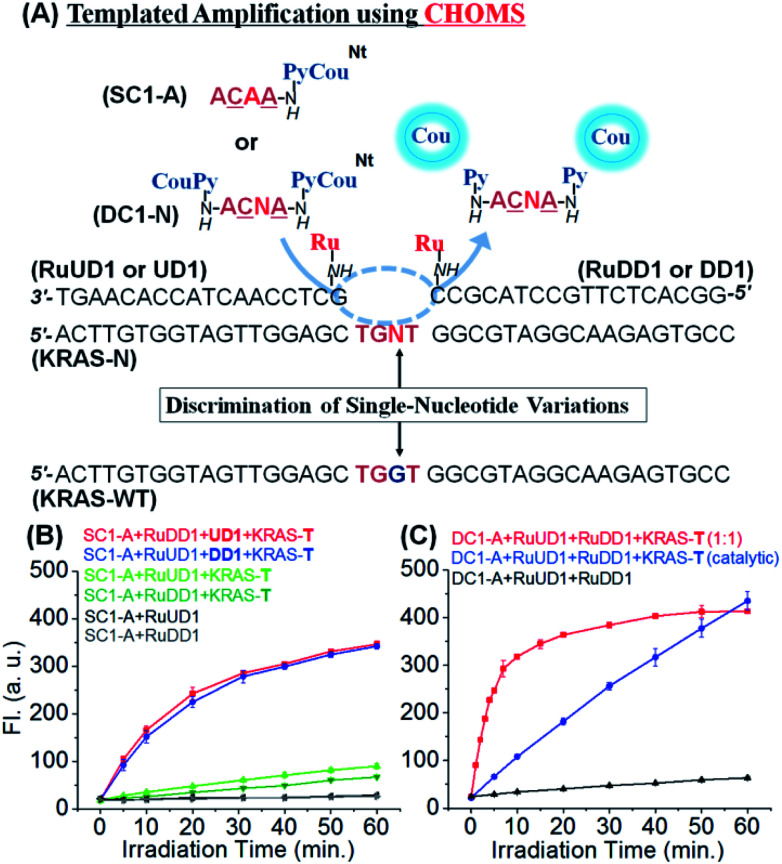
(A) Design of templated reactions with CHOMS targeting *KRAS* variants. SC1 (single coumarin substrate) and DC1 (double coumarin substrate) are PNAs, and the underlined letters denote serine-γ-modified PNA residues; UD1 (upstream DNA guide) and DD1 (downstream DNA guide). (B) Comparison of templated reactions with one (green curves) or two guides (red and blue curves, only one guide with the catalyst and SC1 substrate); conditions: 100 nM of SC1-A and 50 nM of RuUD1, RuDD1, UD1, DD1, and KRAS-T. (C) Templated reaction with two DNA guides functionalized with the catalyst and DC1 substrate at different template loadings; conditions: stoichiometric (red curve): 50 nM of DC1-A, 50 nM of RuUD1 and RuDD1, and 50 nM of KRAS-T; catalytic (blue curve): 100 nM of DC1-A, 5 nM of RuUD1 and RuDD1, and 5 nM of KRAS-T. All templated reactions were performed at pH 7.4 in 1× PBS buffer, 0.01% Tween-20 with 5 mM NaAsc, and 25 °C using a 1 W LED (455 nm).

To investigate the performance of the reaction with this confined hybridization, we initiated our study with a sequence from *KRAS*, a notorious oncogene.^48^ The reaction requires three components: two guide oligonucleotides functionalized with the ruthenium catalyst [Ru(bpy)_2_phenCl_2_] and the substrate. The guide oligonucleotides (18 nt) were prepared from commercial DNAs by functionalizing a 5′ and 3′ amino group (for the upstream and downstream guides, respectively) with the ruthenium catalyst through standard amide coupling (see Fig. 2 for sequences and reaction design). For the substrate, a γ-modified PNA was selected based on its higher duplex stability and sequence discrimination compared to DNA.^49–51^ The templated reduction makes use of a ruthenium-photocatalyzed reduction of pyridinium (Py, Fig. 2) which releases fluorogenic coumarin (Cou).^46^ To evaluate the advantage of the confined hybridization, we compared a templated reaction with only one guide DNA to the reaction with two guides. In order to simplify the analysis, a substrate with only one fluorophore was initially used (SC1-A, Fig. 2A). The reaction with the two guide DNAs was not strongly influenced by the position of the catalyst (upstream or downstream of pyridinium-coumarin; blue and red curves respectively, Fig. 2B). This can be rationalized based on the fact that the substrate covers only 4 nt hybridization, *ca.* 13 Å, and the linkers between the guide DNA and the ruthenium catalyst are 17 atoms (*ca.* 20 Å) and can reach both sides of the substrate. Notably, there was a dramatic difference in the reaction rate when only one guide DNA was used (green curves, Fig. 2B). Compared with the initial rate of reaction (first 5 min), the reaction with two guides proceeded at 16 fluorescent unit per min (red and blue lines, Fig. 2B) which is 9 and 20-fold faster than the reaction with only one guide DNA (1.8 and 0.8 florescent unit per min, light and dark green curves respectively). It is interesting to note the small difference in the rate between the two reactions with only one guide DNA (RuUD1 : upstream DNA *vs.* RuDD1 : downstream DNA). The upstream guide benefits from a larger π-stacking than the downstream guide (purine–purine *vs.* purine–pyrimidine). Taken together, these data strongly support the benefit of the confined hybridization for a minimal substrate was not investigated.

In order to maximize the output of the system, we next investigate the reaction with both guide DNAs functionalized with the catalyst and the substrate bearing two pro-fluorophores (DC1, Fig. 2A). Performing the reaction under stoichiometric conditions (1 equivalent of both guide DNAs, substrate and template) at 50 nM afforded a fast reaction (*t*_1/2_ = 3.5 min; pseudo first order = 3.3 × 10^−3^ s^−1^). The output of this reaction using a catalytic amount of template and guide was clearly superior to that of the same reaction with only a monofunctionalized substrate and guide. For comparison, the same fluorescence output is reached at 30 min using a 10 times lower loading of template and guide DNA (red curve, Fig. 2B*vs.* blue curve Fig. 2C). To gain further insight into the contribution of π-stacking at the interface of the guide DNA and substrate, we compared the rate of reaction using a single guide DNA with adjacent substrate-hybridization to a reaction with a 3 nt gap between the substrate and the guide DNA (Fig. S1†). The reaction with the gap (lacking the π-stacking) was 15-fold slower than the reaction with adjacent hybridization of a single guide. While this may appear contradictory to the prior observations of distance-independence in templated reactions,^42^ including ruthenium-photocatalyzed reactions,^52^ previous reactions made use of substrates that formed thermodynamically stable duplexes with the template and did not necessitate the added interactions provided by π-stacking to benefit from the templating effect.

**Fig. 3:**
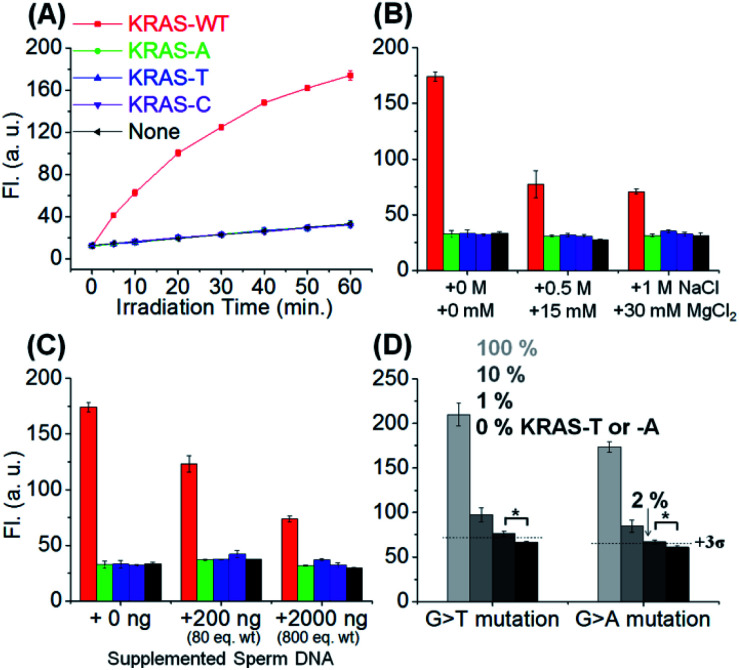
(A) SNV detection in *KRAS* wild type (*KRAS*-WT) *vs.* variants, -A, -T, and -C. Conditions: pH 7.4 1× PBS buffer, 0.01% Tween-20, 5 mM NaAsc, 25 °C, 50 nM of DC1-C, 5 nM of RuUD1 and RuDD1, 1 nM of targets, and irradiation with a 1 W LED (455 nm). Templated reaction has been done in the presence of an (B) additional salt concentration (1 h reaction) or (C) additional single stranded sperm DNA (1 h reaction) to evaluate condition robustness. (D) Detection of KRAS-T or -A in the presence of an excess amount of KRAS-WT sequences and 300 ng of sperm ssDNA. 1 or 2% of KRAS-T or KRAS-A was detectable in 5 nM total target concentration (1 h reaction). Statistics were obtained by an unpaired two-sample *t*-test (*p < 0.05).

Next, we evaluated the SNV discrimination efficiency of the templated reaction with CHOMS. The reaction was performed using a catalytic amount of guide DNAs (5 nM each) and target *KRAS* sequences, including wild-type (G) and A, T, and C variants. As shown in Fig. 3A, the reaction using DC1-C showed exceptional WT-specificity over other sequences with a single mutation. We observed 123-, 92-, and 303-fold discrimination factors between WT and all other mismatched targets (A, T, and C respectively; calculated by dividing the gain of fluorescence for the target sequence by the gain for the mismatch, both values being corrected for the background in the absence of the template). This high level of discrimination is attributed to the difference in the duplex stability of a single base pair mismatch in this short substrate sequence. As a comparison, the reaction was performed with an 8-mer substrate using the same KRAS templates (Fig. S2†). In this case, the discrimination is 1.6–3.2 fold, with a G → A mutation affording the least discrimination and G → T affording the largest discrimination. These results clearly highlight the enhanced performance of the templated reaction with CHOMS. The reactions were also tested with PNA guides rather than DNA guides but the performances were reduced due to higher background reactions, even in the absence of the template (Fig. S3†). The higher background is attributed to stronger non-specific interactions of PNA guides : substrate *vs.* DNA guides : substrate. The reaction was also tested with a shorter substrate (Fig. S4,† 3-mer substrate). While the reaction worked under stoichiometric conditions (50 nM), it performed poorly at a lower templated loading, indicating that this substrate is too short for detection below nanomolar concentrations.

The robustness of the reaction was also tested under different conditions to evaluate its potential application in clinical diagnosis. The addition of MgCl_2_ (up to 30 mM) reduced the overall reaction yield but did not compromise the single nucleotide specificity (Fig. 3B). High concentrations of MgCl_2_ reduce the favourable electrostatic interaction of the positively charged substrate (pyridinium) with the negatively charged template. Addition of sperm ssDNA (up to 800 weight equivalents, Fig. 3C) also led to a small reduction of overall yield without compromising the selectivity. Both of these conditions contribute to reduced interactions of the reaction partners in the templated reaction and thus reduce the overall yield, but do not alter the single nucleotide resolution of the reaction.

We next evaluated the potential CHOMS for low abundance detection of a mutant sequence contaminated with a wild type sequence. The KRAS-T or -A template was mixed with the KRAS-WT template at different ratios (0% to 100%) to measure the minimum concentration for detection. The CHOMS templated reaction enabled the detection of 1–2% (50–100 pM) of SNV sequence within a sample of the WT sequence (Fig. 3D). This is in good agreement with the detection threshold at 60 min reaction time (20 pM and 39 pM after 3 and 1 h respectively, Fig. S5;† detection threshold determined by 3*σ* between the test reaction and control). The results suggest that the CHOMS reaction could be utilized for the direct analysis of PCR amplicons even if heavily contaminated with a WT sequence.

**Fig. 4:**
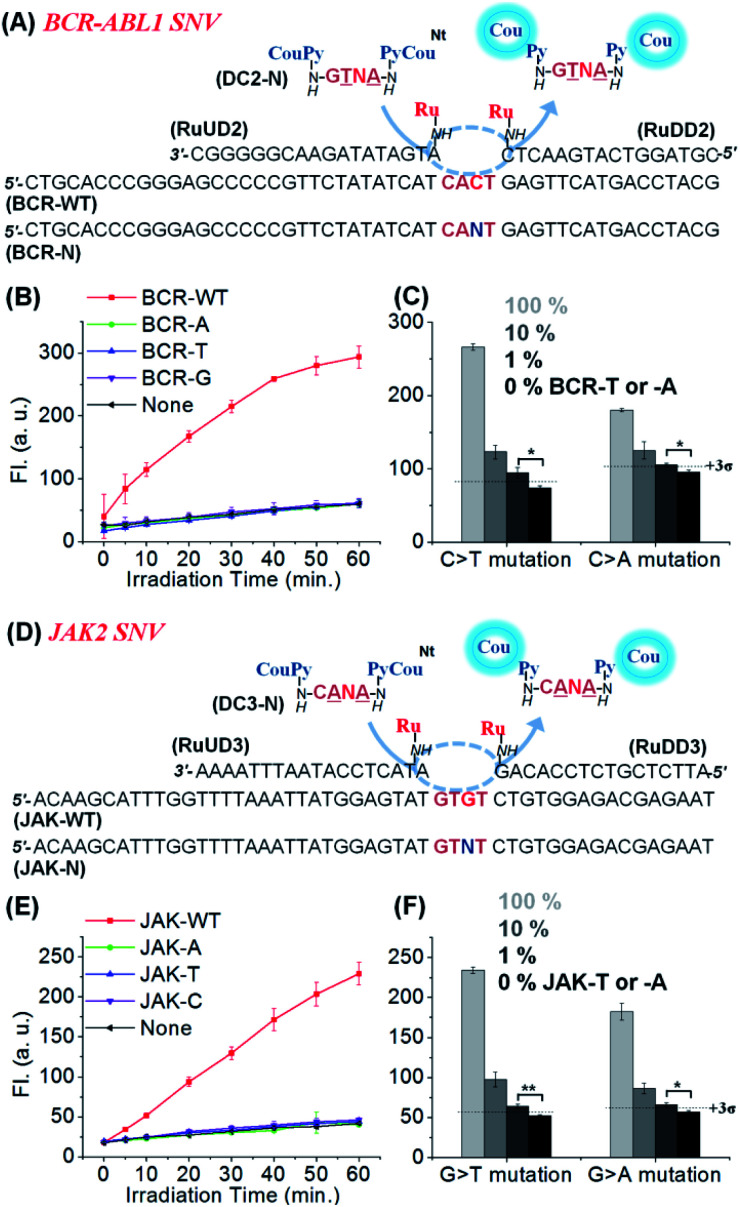
(A) Schematics of the templated reaction with CHOMS for the SNV analysis of *BCR-ABL1* variants. (B) Fluorescence signal from the released coumarin in the presence of BCR-WT and -A, -T, and -G variants (1 nM). Conditions: pH 7.4 1× PBS buffer, 0.01% Tween-20, 5 mM NaAsc, 25 °C, 50 nM of DC2-G, 2.5 nM of RuUD2 and RuDD2, and 200 ng of single stranded sperm DNA, using a 1 W LED (455 nm). (C) Detection of low abundant BCR-T or -A in the presence of an excess amount of BCR-WT. 1% of KRAS-T or KRAS-A was detectable in 5 nM total target concentration (1 h reaction for BCR-T and 90 min for BCR-A). Conditions: pH 7.4 1× PBS buffer, 0.01% Tween-20, 5 mM NaAsc, 25 °C, 50 nM of DC2-N, 5 nM of RuUD2 and RuDD2, 300 ng of single stranded sperm DNA. (D) Schematics of the templated reaction with CHOMS for the SNV analysis of *JAK2* variants. (E) Fluorescence signal in the presence of JAK-WT and -A, -T, and -C variants (1 nM). Conditions: pH 7.4 1× PBS buffer, 0.01% Tween-20, 5 mM NaAsc, 25 °C, 50 nM of DC3-C, 5 nM of RuUD3 and RuDD3, and 200 ng of single stranded sperm DNA using a 1 W LED (455 nm). (F) Detection of low abundant JAK-T or -A in the presence of an excess amount of JAK-WT. 1% of JAK-T or -A was detectable in 5 nM total target concentration (1 h reaction). Conditions: pH 7.4 1× PBS buffer, 0.01% Tween-20, 5 mM NaAsc, 25 °C, 50 nM of DC3-N, 5 nM of RuUD3 and RuDD3, and 300 ng of single stranded sperm DNA. The detection threshold line was set as 3 times the standard deviation (3*σ*) of the blank reaction. Statistics were obtained by an unpaired two-sample *t*-test (*p ≤ 0.05 and **p ≤ 0.01).

We applied the same reaction design to other SNVs relevant in cancer therapy: JAK2 V617F (1849 G/T),^53^ and *BCR-ABL1* T315I (944 C/T)^9^ as shown in Fig. 4. In both cases, clear discrimination of WT from other variants was observed using similar conditions to those previously used for *KRAS*. High discrimination factors were observed for the detection of all permutations: average 178-fold for *BCR-ABL1* (309, 90, and 135-fold to -A, -T, and -G SNV, respectively) and 96-fold for JAK2 (163, 81, and 44-fold for -A, -T, and -C SNV, respectively). These high discrimination factors enable the detection of a mutant template at low concentration even if contaminated with the WT template (Fig. 4C and F). Moreover, substrates designed for the detection of the mutant rather than the WT sequence performed equally well (Fig. S6, see Table S2† for the analysis of the 27 permutations of sequences). The high selectivity of the reaction enabled the detection of 1% (50 pM) A- or T-mutant sequence of *BCR-ABL1* and *JAK2* in the presence of 99% of the corresponding WT types within 1 h irradiation time (Fig. 4C and F). These results show that CHOMS reactions are able to achieve rapid detection of low abundant variants (50 pM) with high discrimination, across a large cross-section of substrate sequences.

**Fig. 5:**
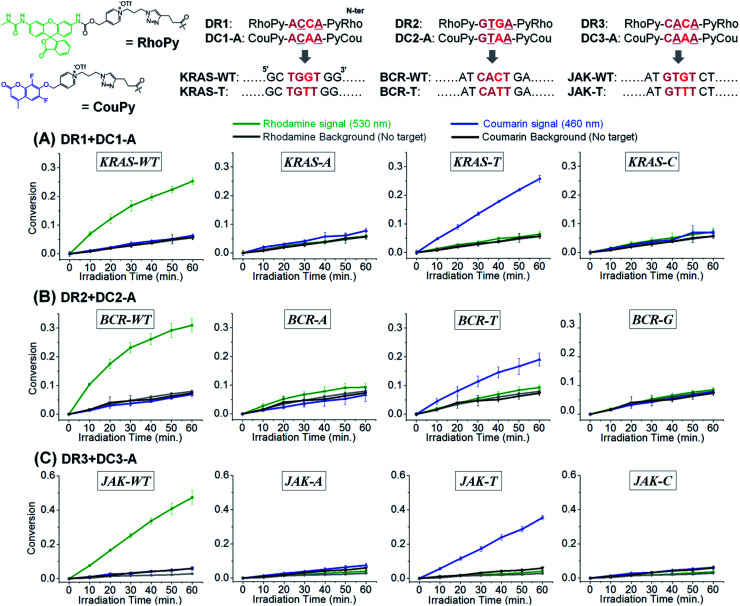
Two-colour system in templated reactions with CHOMS using 4-mer PNAs releasing rhodamine or coumarin for simultaneous detection of two different SNVs (WT and T variants) of (A) *KRAS*, (B) *BCR-ABL1*, and (C) *JAK2*. Conditions: pH 7.4 1× PBS buffer, 0.01% Tween-20, 5 mM NaAsc, 25 °C, 50 nM of each coumarin and rhodamine 4-mer PNAs, 5 nM of corresponding RuUD and RuDD (total 10 nM of Ru), 1 nM of target sequences, and 300 ng of single stranded sperm DNA. Fluorescence measurement: *λ*_exc_: 490 nm, *λ*_emi_: 530 nm, and cutoff: 515 nm for rhodamine; *λ*_exc_: 360 nm, *λ*_emi_: 460 nm, and cutoff: 455 nm for coumarin.

Target SNP or SNV detection could be further enhanced by the simultaneous reaction of two substrates targeting the WT and SNV simultaneously. This would allow a ratio-metric analysis that would further enhance the discrimination factor of SNVs. We opted for a coumarin and rhodamine that are spectroscopically resolved (em: 460 and 530 nm respectively) and can be uncaged with the same pyridinium chemistry. To this end, we further synthesized 4-mer PNAs having a rhodamine-pyridinium^54^ linker, instead of coumarin, to produce DR1, 2, and 3, targeting the WT of *KRAS*, *BCR-ABL1*, and *JAK2*, respectively (Fig. 5). 4-mer PNA targeting sequences having any types of SNV were functionalized with pyridinium coumarin (DC-A sequences) for second signalling and medically relevant T variations in *KRAS*, *BCR-ABL1*, and *JAK2* were selected as test SNVs.^16,27^ After 10 min of irradiation, a sample containing both coumarin and rhodamine substrates selectively afforded a fluorescence signal exclusively for the fully matched sequences (WT for rhodamine and SNV for coumarin). Other variants afforded a negligible signal gain in either channel. For a test considering an SNV *vs.* WT, the discrimination is now augmented by the signal in the rhodamine channel relative the signal in the coumarin channel. Applying this analysis to *KRAS*, the discrimination factor for WT *vs.* T genotype goes from 31 to 1022. The same analysis for *BCR* and *JAK* (WT *vs.* T genotype) yielded a discrimination factor of 1113 and 3148 respectively. An alternative analysis is to use a two samples *t*-test (*i.e.* the probability of false genotyping). Based on the triplicate experiments, the probability of a false genotype is between 0.001 and 0.00002 (see Table S2† for the analysis of different permutations). The performance of CHOMS templated reactions compares favourably to values reported for the same genotyping (224 and 680 for *KRAS*,^55,56^ 4.7 for *BCR-ABL1*,^27^ 9.3, 26, 14, and 615 for *JAK2*).^27,57^ We further showed that the reaction kinetics of DC1-A with the WT template was not affected by the presence of a competing substrate (DR1, Fig. S7†) and that the presence of two templates could be detected simultaneously (Fig. S8†).

**Fig. 6:**
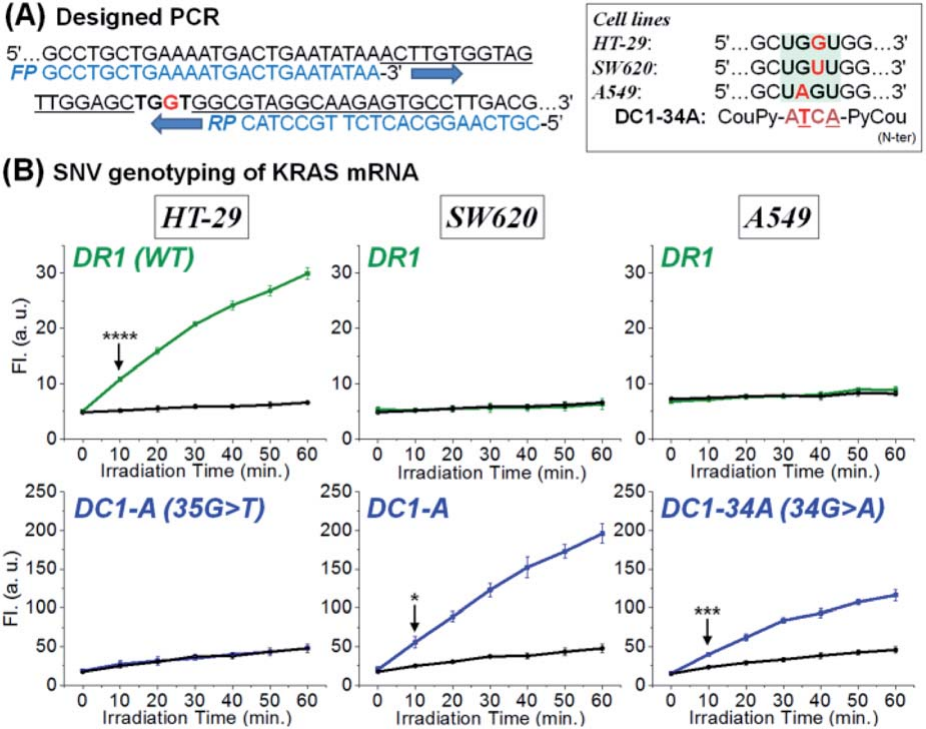
Two-colour templated reaction with CHOMS using RT-PCR products of total RNA extracted from cell lines, HT-29, SW620, and A549. (A) Design of asymmetric PCR for a 71-mer product containing an identical sequence to *KRAS* mRNA and a mutation type in *KRAS* mRNA depending on cell lines. (B) SNV genotyping of *KRAS* mRNA using a two-colour templated reaction with CHOMS. Reaction samples containing DR1 + DC1-A afforded a rhodamine or coumarin fluorescence signal in the presence of the PCR product obtained from HT-29 and SW620, respectively. DC1-34A, targeting a 34 G > A mutation of *KRAS*, afforded a coumarin fluorescence signal in the presence of the sample of the A549 cell line. Black line corresponds to the background (no target, only buffer). Conditions for the two-colour system: pH 7.4 1× PBS buffer, 0.01% Tween-20, 5 mM NaAsc, 25 °C, 50 nM of DR1 and 50 nM of DC1-A for HT-29 or SW620 or 50 nM of DR1 and 50 nM of DC1-34A for A549, 5 nM of each RuUD1 and RuDD1, 20 μL of PCR sample, and total 200 μL. Statistics were obtained by an unpaired *t*-test with unequal variances (*p = 0.012, ***p = 3.83 × 10^−4^, and ****p = 8.66 × 10^−5^).

Inspired by the sensitivity and selectivity of CHOMS, we investigate its performance on an amplicon from a cellular extract as could be performed in the case of a biopsy. We opted to analyse the SNV status of *KRAS* across different cell lines. As the first step in this diagnosis, total RNA was extracted from HT-29 (*KRAS* wild-type),^58,59^ SW620 (*KRAS* p.G12V, c.35 G > T mutation),^60,61^ and A549 (*KRAS* p.G12S c.34 G > A mutation, see Fig. 6A)^62,63^ and the total RNA of each cell line (sample corresponding to *ca*. 10 mm^3^) was subsequently amplified by reverse transcription and asymmetric PCR using an excess amount of forward primers (see Fig. S9† for PAGE analysis of the PCR product).^64^ The resulting PCR mixture containing 71-mer single-stranded DNA (Fig. 6A), which is an identical sequence to the targeted mRNA region, was directly assessed using a CHOMS reaction without further manipulations. Using two-colour reactions (rhodamine for WT and coumarin for a mutation), the presence of a mutation was unambiguously (*p* = 8.66 × 10^−5^) observed after 10 min of reaction for SW620 and A549 while HT29 was WT (Fig. 6B). The capability of CHOMS for high precision genotyping is clearly illustrated by the control cell line, A549, having a different type of mutation (34 G > A) than SW620. The PCR amplicon of A549 produced no fluorescence signal from both DR1 and DC1-A (WT and SW620 mutant) but yielded an unambiguous reaction with DC1-34A, 4-mer coumarin PNA fully matched to the 34 G > A mutated region of *KRAS* (Fig. 6B, right and see Fig. S10† for all negative control experiments). Taken together, these results demonstrate that CHOMS reactions with PCR amplicons are capable of direct analysis of cellular samples. Moreover, the two-colour system could be utilized for the diagnosis of heterozygosity of genetic samples (simultaneous detection of the WT and mutant type). While this analysis was performed with a PCR amplification of the sample, the accuracy of genotyping is not dependent on the fidelity of primer hybridization/extension as is the case for allele-specific PCR.^65^ Previously, a templated reaction based on native chemical ligation was shown to be sufficiently robust to withstand the thermocycling of PCR and could be used as a real-time readout of PCR progression with SNP accuracy.^38^ In this example, probes designed to respond to either WT-RAF or mutant-RAF required a different number of PCR cycles to yield a positive signal with an equal DNA input. The two-step process used in the current study is important because it dissociates the sensitivity (detection threshold) from the accuracy (signal from perfect match *vs.* mismatch). In PCR-based analyses, these two parameters are intertwined and are both reflected in the number of PCR cycles required to reach a threshold amplification. Thus, interpretation of the readout must be calibrated to the initial concentration of a control reaction. In the present case (PCR; CHOMS detection), the amplification can be driven to completion by excess cycles prior to the templated reaction and SNV/SNP analysis.

## Conclusions

In summary, we demonstrate a templated reaction with constrained hybridization of minimal substrates (CHOMS). We show that 4-mer-γ-modified PNAs are sufficient for nucleic acid sensing down to 20 pM of analyte provided that it is constrained between two guide DNAs. These short substrates provided exceptional discrimination of SNPs or SNVs (average > 100-fold for three different oncogenes) which can be carried out on a PCR amplicon from cell extracts without further purification. Compared to binary probes and previous designs of templated reactions, CHOMS has unique and important advantages: (1) π-stacking on either side of the confined hybridization enables a short substrate with a high sequence discrimination and turn-over (signal amplification); (2) there is no trade-off between the length of the nucleic acid considered and the sensitivity of the SNP since the sensing is based on the hybridization of a constant 4-mer while the overall length is dictated by the guide DNA and promiscuous hybridization of the guide DNA is unlikely to provide a 4 nt gap for the reaction; (3) the presence of two guide DNAs confines the reaction to a unique position in a genome; (4) the design is simple and was found to operate with exceptional sensitivity and discrimination across 27 tested combinations of SNVs suggesting that it does not require the optimization of temperature or concentration for new analytes; (5) the reaction is robust and performed under a range of conditions (MgCl_2_ content in buffer, excess non-complementary DNA, and other biomolecules present in crude RNA extracts). The reaction with amplicons from the cell extract afforded a distinguishable signal in 10 min (*p* = 8.66 × 10^−5^ for WT *vs.* SNV). Considering the advent in ultrafast PCR reactions (30 cycles in 5 min using photonic PCR^66^), it should be possible to perform a diagnosis under 30 min from a biopsy. Furthermore, the fact that high fidelity amplification can be used prior to genotyping suggests that this technology will be compatible with liquid biopsies.

## Conflicts of interest

There are no conflicts to declare.

## Supplementary Material

SC-011-D0SC00741B-s001
