# Supplementary material for: Enhanced SNP-sensing using DNA-templated reactions through confined hybridization of minimal substrates (CHOMS)
Source: Chem Sci. 2020 Mar 24;11(16):4150–7. doi: 10.1039/d0sc00741b (PMC8152519; doi:10.1039/d0sc00741b)
Supplement: SC-011-D0SC00741B-s001 [file SC-011-D0SC00741B-s001.pdf]

Electronic Supplementary Material (ESI) for Chemical Science.
This journal is © The Royal Society of Chemistry 2020

Supporting Information

Enhanced SNP-sensing using DNA-templated reactions through confined hybridization of minimal substrates (CHOMS)

Ki Tae Kim and Nicolas Winssinger*

Department of Organic Chemistry, NCCR Chemical Biology, Faculty of Science, University of Geneva, 30 quai Ernest-Ansermet, 1205 Geneva, Switzerland

Section Page

1. Materials and methods S2
1.1 General information S2
1.2 DNA-templated reaction S3
1.3 Total RNA extraction from cells and RT-PCR S4
1.4 Information of sequences (Table S1) S5
2. Results S6
Figure S1 Templated reaction having no base stacking between PNA and guide DNA S6
Figure S2 Canonical templated reaction using RuUD-s, SC-8, KRAS-WT S7
Figure S3 PNA-version of CHOMS templated reaction using RuUP1, RuDP1, and KRAS-WT S8
Figure S4 CHOMS templated reaction using 3-mer gap, RuUD-3M, RuDD1, DC-3M, KRAS-WT S9
Figure S5 Detection limit of CHOMS templated reactions using RuUD1, RuDD1, DC1 S10
Figure S6 CHOMS templated reaction for selective discrimination of SNVs S11
Figure S7 CHOMS templated reaction using DC1-A, RuUD1, RuDD1, and KRAS-T S12
Figure S8 Two-color system of CHOMS templated reactions S13
Figure S9 Analysis of PCR products by 10 % 8M urea denaturing polyacrylamide gel electrophoresis S14
Figure S10 Two-color CHOMS templated reaction using RT-PCR products S15
Table S2 Discrimination factor and two sample unpaired t-test S16
3. Mass spectra of the synthesized DNAs and PNAs S17
4. References S40

1. Materials and methods

1.1 General information

Ruthenium catalyst-N-hydroxysuccinimide ester (**2**) for DNA conjugation has been synthesized by a synthetic scheme below. Detailed procedures are shown in our previous report.[1]

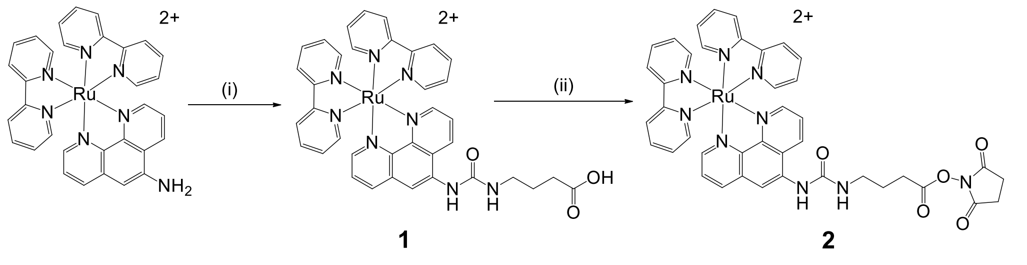

Reagents and conditions: (i) a) triphosgene (0.5 eq), diisopropylethylamine (4.0 eq), DCE, r.t., 4 h. b) γ-aminobutyric acid (3.0 eq), DMF, r.t., 12 h, 14 %; (ii) *N*-hydroxysuccinimide (1.5 eq), EDC•HCl (1.3 eq), DCM/DMF, r.t., 2 h, 95%;

Preparation and identification of Ru-modified DNA and coumarin- or rhodamine-modified PNA have been done according to our previous report.[1] All RNA and DNA samples were purchased from Eurogentec with reverse phase HPLC purification step (85 % of purity by supplier's specifications). The purity of oligonucleotides was monitored by A_{260}/A_{280} (ratio of 1.7–2.0) and gel electrophoresis. Synthetic target DNAs were used for experiment without further purification step. For the ruthenium-conjugated DNA (guide DNA), 14 mM of Ru-NHS in DMSO, 0.091 M of pH 8.5 NaB buffer, and 400 μM of amino-modified (C6 linker) hairpin DNA were prepared. 20 μL of DNA solution, 60 μL of NaB buffer, and 20 μL of Ru-NHS solution were mixed well in a microtube and the mixture was incubated in room temperature for 12 h. After reaction, the mixtures were directly injected into HPLC. An Agilent high-performance liquid chromatography system (1260 Series) was used to purify the labelled DNAs; Agilent, ZORBAX 300SB-C18 column (9.4 x 250 mm); gradient elution: 0 min, A:B = 100:0; 28 min, A:B = 50:50; 29 min, A:B = 0:100; 30 min, A:B = 100:0; solution A: 0.1 M pH 7.3 TEAA buffer; solution B, 0.1 % TFA in HPLC grade acetonitrile; flow rate: 3.0 mL/min; UV detection: 260 nm and 455 nm for Ruthenium complex labelled DNAs. Detailed structures are shown below.

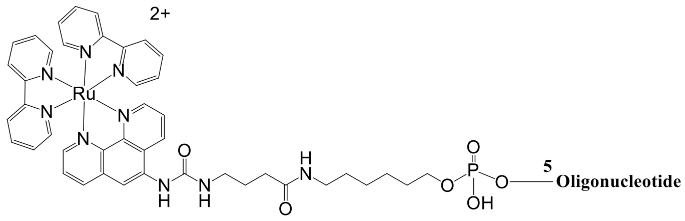

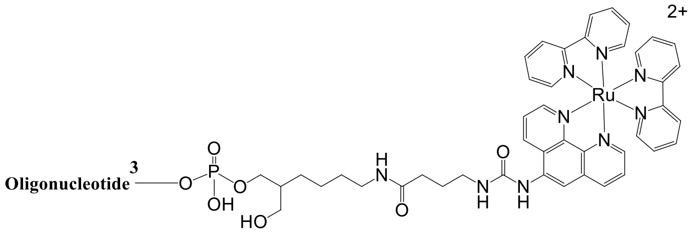

For conjugation of Ru with PNAs, a mixture of 15 μL of 14 mM Ru catalyst NHS ester, 6.3 μL of 10 % (v/v) TEA in DMF, and 100 μL of DMF was treated to amino-modified PNAs on 1 mg of Rink amide resin and incubated for overnight. PNAs and DNAs were purified by HPLC and used without further manipulations. Azido-pyridinium-coumarin and azido-pyridinium-rhodamine were synthesized according to our previous synthetic procedures.[2,3] LC-MS spectra were recorded by using a DIONEX Ultimate 3000 UHPLC coupled with a Thermo LCQ Fleet Mass Spectrometer System (electrospray ionization (ESI)) operated in positive. A Bruker Daltonics Autoflex spectrometer was used for MALDI-TOF mass results.

Nanodrop 2000c spectrophotometer (Thermo Fisher Scientific) was used to determine the DNA and PNA concentrations. The absorbance at 260 nm of the sample was measured. For quantification of oligonucleotides, 13700, 6600, 11700, 8800 M^{-1} were used as extinction coefficient at 260 nm for A, T, G, C, respectively. For quantification of PNAs containing pyridinium coumarin, the absorption by natural nucleobases (A, T, G, and C) at 260 nm was calculated by followed equation: $A_{260\,(A,\,T,\,G,\,C)} = A_{260} - 1.15 \times A_{315}$. Weight of dried PNAs and calibration curve[3] were used for quantification of PNAs containing pyridinium rhodamine.

1.2 DNA-templated reaction

Reactions were performed in 1×PBS buffer: pH 7.4, 0.01% tween-20, 5 mM sodium ascorbate, 25 °C. Each experiment was performed in triplicates. Stock solutions of each reaction component were prepared in water at 0.5 μM. In a plastic 96-well plate (standard opaque), 50 μL of pH 7.4 4×PBS buffer, 10 μL of 0.2 % Tween-20, 10 μL of 100 mM sodium ascorbate, and 2 or 3 μL of 100 ng/μL single stranded sperm DNA were added to the reaction well. To the mixture, the stock solution of RuUD, RuDD, target SNV sequence (or PCR amplicons), and 4-mer coumarin or rhoamine PNA (DC or DR) were added to the desired concentration (final volume 200 μL). Water was added to reach the 200 μL of reaction volume. Final mixtures were mixed well using a micropipette and directly applied to templated reaction without

further manipulation.

In case of low abundant detection experiment, 0.5 μM stock solutions of wild type and mutant type were mixed in different ratio (0 % to 100 %) and then 2 μL of the resulting mixture was used for the templated reaction (5 nM of total target concentration at 200 μL volume).

Fluorescence of the samples was measured immediately after irradiation. Molecular Devices Spectra Max M5 were used for measurement of fluorescence intensities with following parameters (For coumarin: λ_{ex}: 360 nm, λ_{em}: 460 nm, cutoff: 455 nm, PMT gain: medium, flash per read: 6, shake 5 sec before first read; for rhodamine: λ_{ex}: 490 nm, λ_{em}: 530 nm, cutoff: 515 nm, PMT gain: medium, flash per read: 6, shake 5 sec before first read). The well-plate was irradiated with a collimated LED light 10 cm above the plate (455 nm, 1W: Thorlabs, part number M455L2-C1 – www.thorlabs.com). The percentage of conversion was calculated based on a titration curve of coumarin or rhodamine. We validated that the reaction reaches a plateau and that the fluorescent units of this plateau indeed correspond to 100% yield according to the titration curve.

For calculation of discrimination factor (DF), we adopted the equation, DF = (signal of WT target – signal of control (no target)) / (signal of SNV target – signal of control (no target)), using the Fl. values obtained at 60 min. of reaction time.

1.3 Total RNA extraction from cells and RT-PCR

Total RNA extraction from each HT-29, SW620, A549 cell line was done by RNeasy Mini Kit (QIAGEN) using 5×10^6 cells. 5 μg of total RNA was converted to cDNA of total mRNA by using oligo(dT)$_{15}$ of Reverse Transcription System (Promega). The mixture of cDNA was directly used for PCR using conditions: 1 μL of aliquot of cDNA mixture, 5 μL of 5×Phusion HF buffer (ThermoScientific), 0.5 μL of 10 mM dNTP mix (ThermoScientific), 1 μL of 10 μM forward primer (FP), 1 μL of 1.25 μM reverse primer (RP), 1U Phusion High-Fidelity DNA Polymerase (ThermoScientific), 17 μL of nuclease-free water, total 26 μL. Cycling conditions: 1) 95 °C for 5 min, 2) 35 cycles of 95 °C for 30 sec min, 60 °C for 30 sec, 72 °C for 30 sec, 3) 72 °C for 4 min. 20 μL of the PCR product was directly applied to templated reaction analysis or PAGE without any purification step.

1.4 Information of sequences

Table S1 RNA, DNA and PNA sequences investigated in this study

Name	Sequence (5′ to 3′ for RNA and DNA, N- to C-terminal for PNA)
**RuUD1**	Ru-GCTCCAACTACCACAAGT
**RuDD1**	GGCACTCTTGCCTACGCC-Ru
**RuUD-s**	Ru-CTCCAACTACCACAAGT
**RuDD-s**	CCTACGCCACCAGCTCCA-Ru
**SC1-A**	CouPy-Lys-AACA
**DC1-C**	CouPy-Lys-ACCA-Lys-PyCou
**SC-8**	CGCCACCA-Lys-Cou
**KRAS-WT**	ACTTGTGGTAGTTGGAGC TGGT GGCGTAGGCAAGAGTGCC
**KRAS-A**	ACTTGTGGTAGTTGGAGC TGAT GGCGTAGGCAAGAGTGCC
**KRAS-T**	ACTTGTGGTAGTTGGAGC TGTT GGCGTAGGCAAGAGTGCC
**KRAS-C**	ACTTGTGGTAGTTGGAGC TGCT GGCGTAGGCAAGAGTGCC
**UD1**	NH_2-GCTCCAACTACCACAAGT
**DD1**	GGCACTCTTGCCTACGCC-NH_2
**RuUP1**	Ru-Lys-GCTCCAACTACC
**RuDP1**	Ac-CTTGCCTACGCC-Lys-peg-Ru
**DC-3M**	CouPy-Lys-ACC-Lys-PyCou
**RuUD-3M**	Ru-AGCTCCAACTACCACAAG
**DC2-G**	CouPy-Lys-AGTG-Lys-PyCou
**RuUD2**	Ru-ATGATATAGAACGGGGGC
**RuDD2**	CGTAGGTCATGAACTC-Ru
**BCR-WT**	CTGCACCCGGGAGCCCCCGTTCTATATCAT CACT GAGTTCATGACCTACG
**BCR-A**	CTGCACCCGGGAGCCCCCGTTCTATATCAT CAAT GAGTTCATGACCTACG
**BCR-T**	CTGCACCCGGGAGCCCCCGTTCTATATCAT CATT GAGTTCATGACCTACG
**BCR-G**	CTGCACCCGGGAGCCCCCGTTCTATATCAT CAGT GAGTTCATGACCTACG
**DC3-C**	CouPy-Lys-ACAC-Lys-PyCou
**RuUD3**	Ru-ATACTCCATAATTTAAAA
**RuDD3**	ATTCTCGTCTCCACAG-Ru
**JAK-WT**	ACAAGCATTTGGTTTTAAATTATGGAGTAT GTGT CTGTGGAGACGAGAAT
**JAK-A**	ACAAGCATTTGGTTTTAAATTATGGAGTAT GTAT CTGTGGAGACGAGAAT
**JAK-T**	ACAAGCATTTGGTTTTAAATTATGGAGTAT GTTT CTGTGGAGACGAGAAT
**JAK-C**	ACAAGCATTTGGTTTTAAATTATGGAGTAT GTCT CTGTGGAGACGAGAAT
**DR1**	RhoPy-Lys-ACCA-Lys-PyRho
**DC1-A**	CouPy-Lys-AACA-Lys-PyCou
**DC1-T**	CouPy-Lys-ATCA-Lys-PyCou
**DR2**	RhoPy-Lys-AGTG-Lys-PyRho
**DC2-A**	CouPy-Lys-AATG-Lys-PyCou
**DC2-T**	CouPy-Lys-ATTG-Lys-PyCou
**DR3**	RhoPy-Lys-ACAC-Lys-PyRho
**DC3-A**	CouPy-Lys-AAAC-Lys-PyCou
**DC3-T**	CouPy-Lys-ATAC-Lys-PyCou
**DC1-34A**	CouPy-Lys-ACTA-Lys-PyCou
**FP**	GCCTGCTGAAAATGACTGAATATAA
**RP**	CGTCAAGGCACTCTTGCCTAC

[a] L-serine modified PNA is marked with underline

2. Results

Figure S1. Templated reaction having no base stacking between PNA and guide DNA (3-mer gap between **DC1-C** and **RuDD-s**). This experiment was designed to observe reaction at non-targeted site by protection of the KRAS SNV site using **RuDD-s**. Comparing the initial rate of reaction , reaction without stacking showed significantly slower fluorescent enhancement (15-fold) than reaction with single stacking (**DC1-C+RuUD1**) in 1:1 stoichiometry. None of the reactions showed fluorescent enhancement when catalytic amount of **RuDD-s** was used. Conditions: 1×PBS buffer pH 7.4, no divalent cation, 0.01% tween-20, 5 mM NaAsc, 25 °C. 50 nM of **DC1-C**, **RuUD1**, **RuDD-s**, **and KRAS-WT** were used for 1:1 reaction. 50 nM of **DC1-C**, 5 nM of **RuDD-s**, and 1 nM of **KRAS-WT** were used for catalytic reaction.

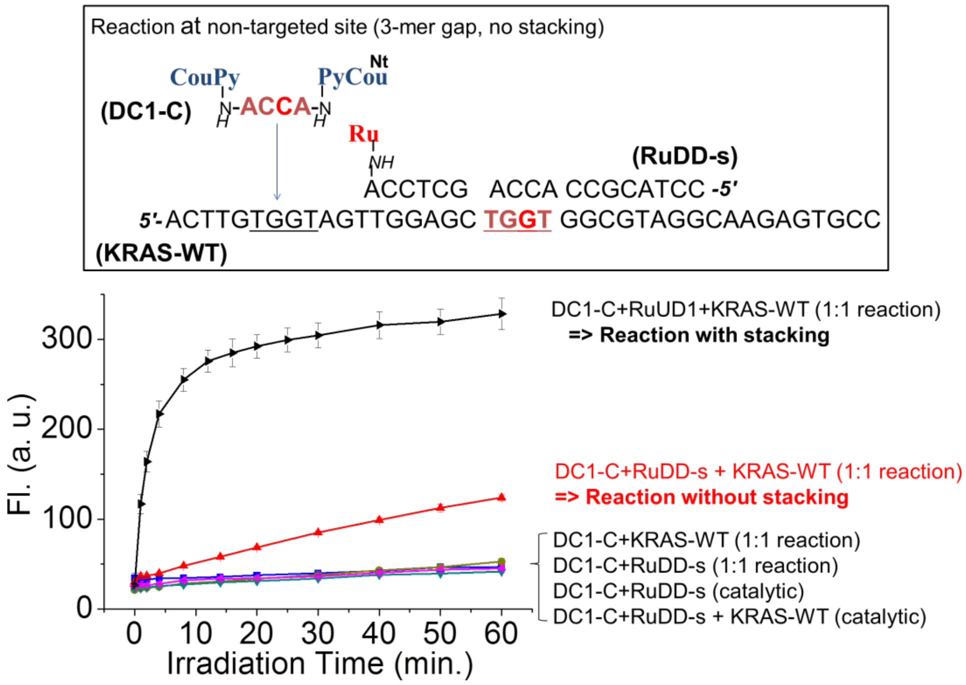

Figure S2. Templated reaction with binary probes using **RuUD-s**, **SC-8**, **KRAS-WT**. (a) 1:1 reaction using 50 nM of each component. (b) Catalytic reaction using 20 nM of **SC-8**, 12.5 nM of **RuUD-s**, 12.5 nM of **KRAS-WT**, Conditions: 1×PBS buffer pH 7.4, no divalent cation, 0.025% tween-20, 5 mM NaAsc, 25 °C. 8-mer PNA templated reaction is not sensitive to KRAS mutations due to low sequence selectivity of 8-mer PNA and mismatched hybridization.

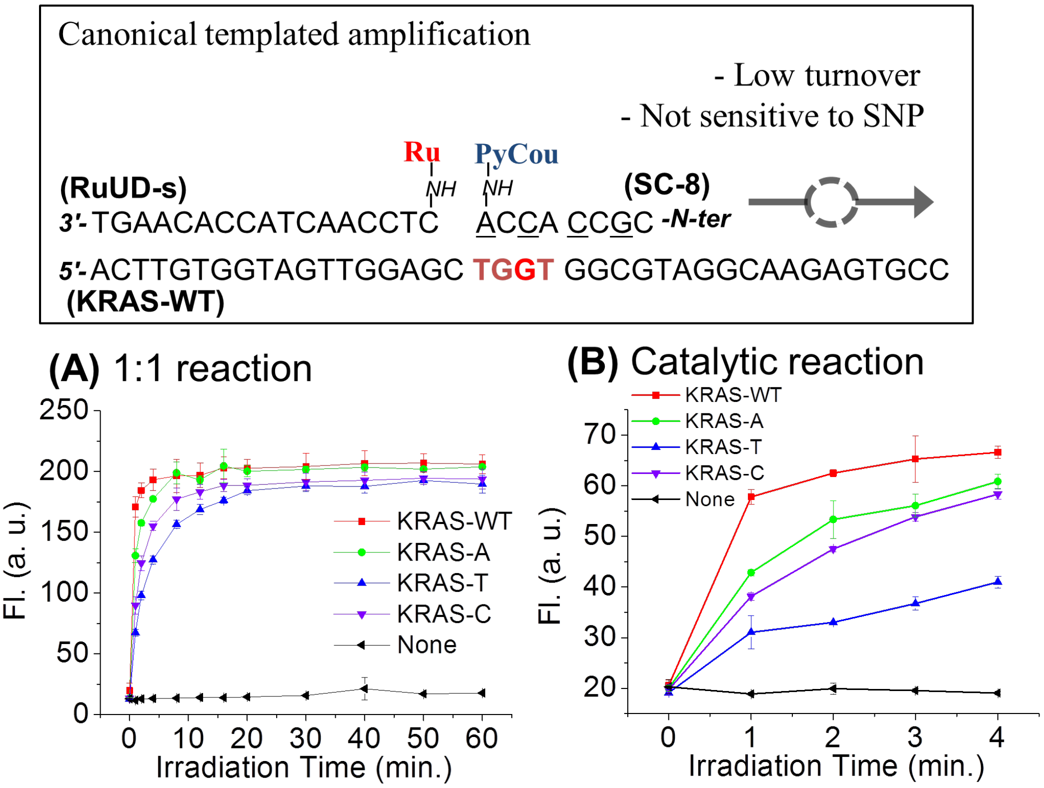

Figure S3. PNA-version of CHOMS templated reaction using **RuUP1**, **RuDP1**, and **KRAS-WT**. (a) 1:1 reaction using 50 nM of each component. (b) Catalytic reaction using 50 nM of **DC1-C**, 5 nM of **RuUP1** and **RuDP1**, 1 nM of **KRAS-WT** or mutated sequences, Conditions: 1×PBS buffer pH 7.4, no divalent cation, 0.01% tween-20, 5 mM NaAsc, 25 °C.

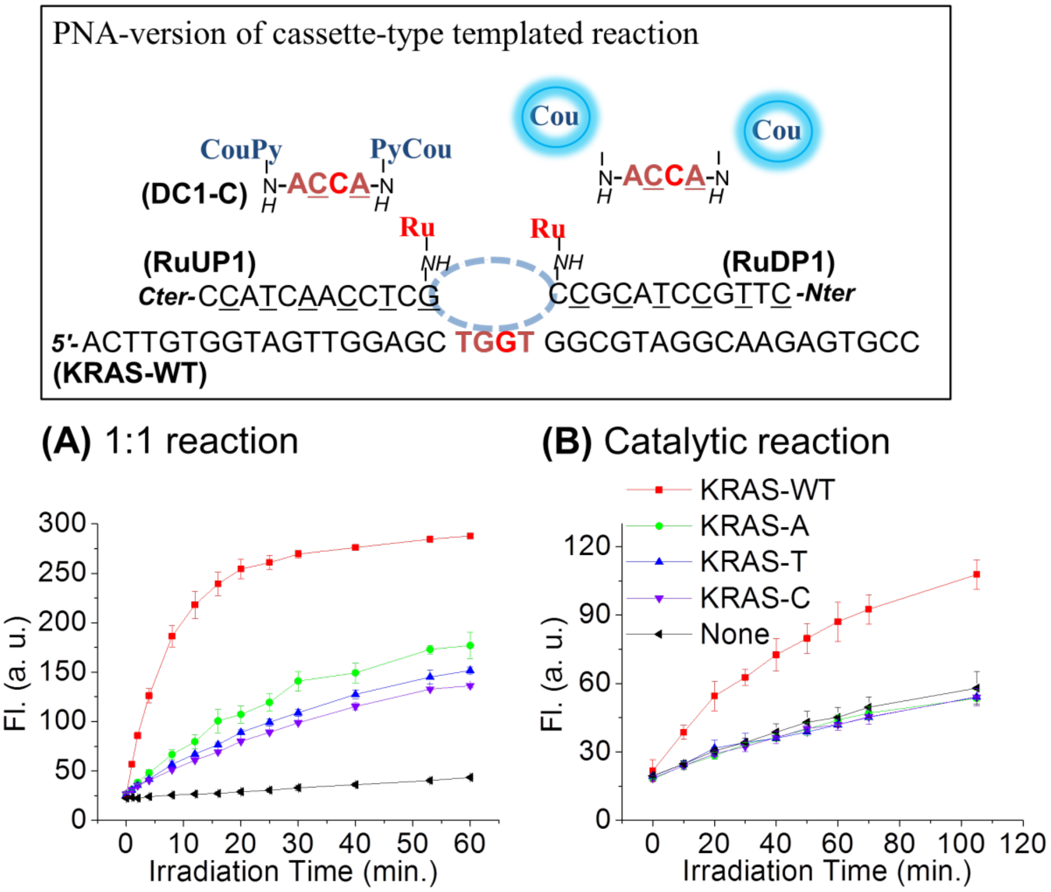

Figure S4. CHOMS templated reaction using 3-mer gap, **RuUD-3M**, **RuDD1, DC-3M, KRAS-WT**. Conditions for 1:1 reaction: 50 nM of each reaction component, 1×PBS buffer pH 7.4, no divalent cation, 0.01% tween-20, 5 mM NaAsc, 25 °C.

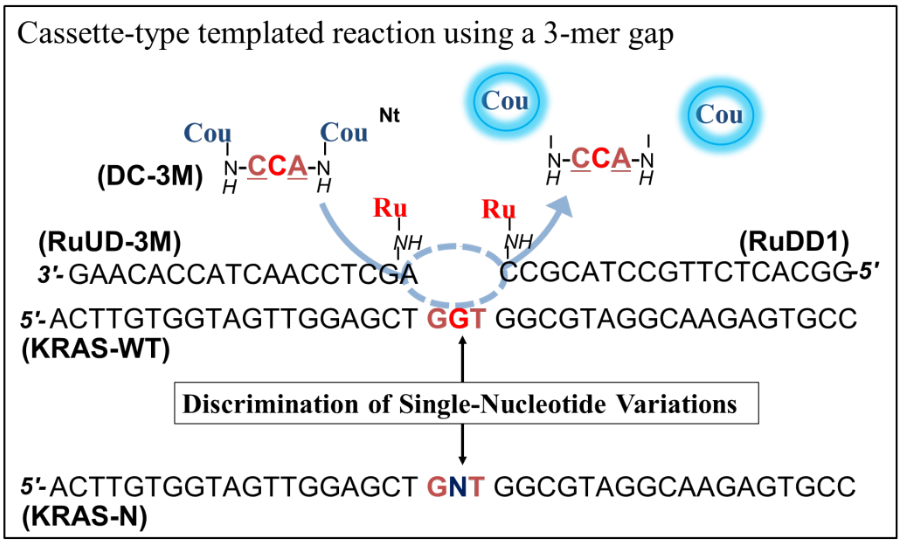

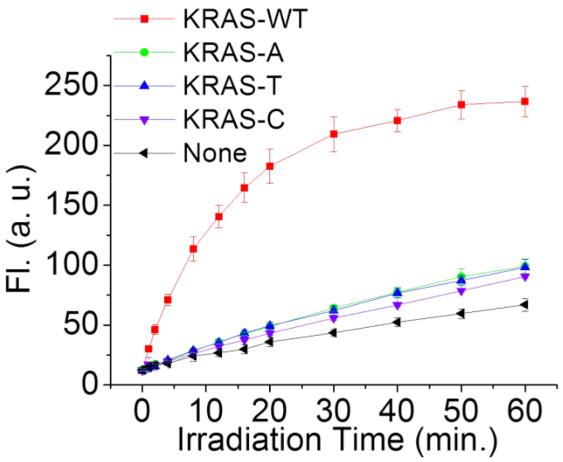

Figure S5. Detection limit of CHOMS templated reactions using **RuUD1**, **RuDD1**, **DC1-C**, and **KRAS-WT**. Conditions: 1×PBS buffer pH 7.4, no divalent cation, 0.01% tween-20, 5 mM NaAsc, 25 °C, 50 nM of **DC1-C**, 5 nM of **RuUD1** and **RuDD1**. 20 pM or 40 pM of detection threshold was observed after 3 h or 1 h of irradiation time, respectively.

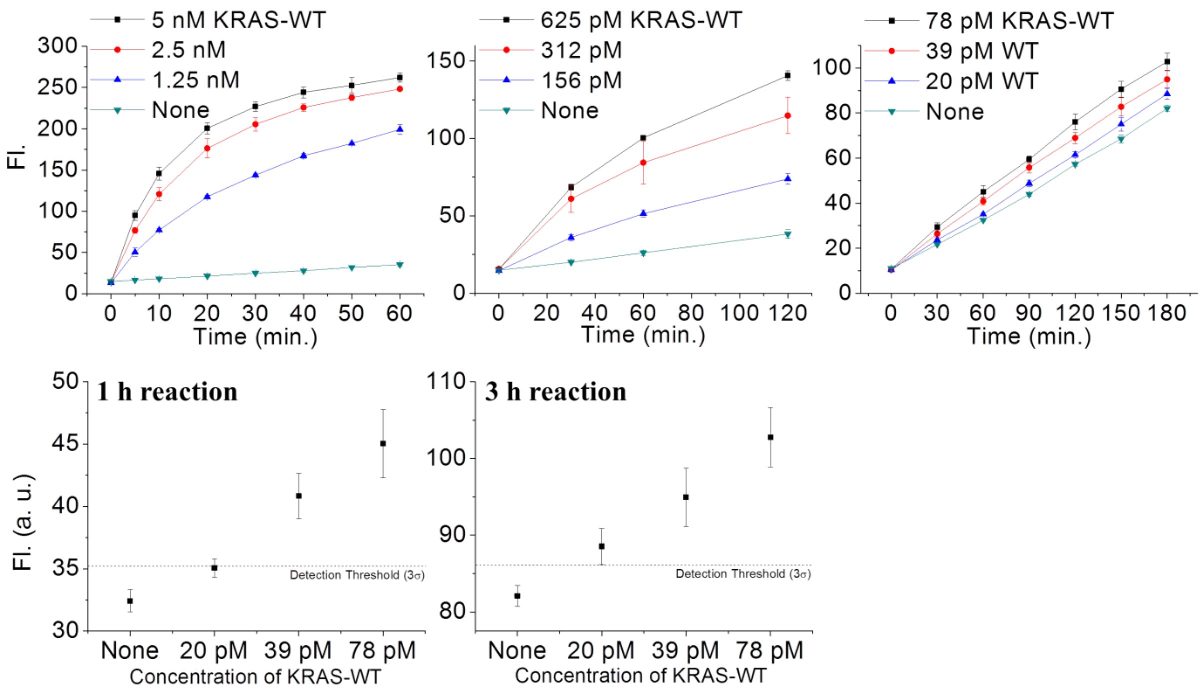

Figure S6. CHOMS templated reaction for selective discrimination of (a) **KRAS-T**, (b) **KRAS-A**, (c) **BCR-T**, (d) **BCR-A**, (e) **JAK-T**, (f) **JAK-A** from other SNVs by using **DC1-A**, **DC1-T**, **DC2-A**, **DC2-T**, **DC3-A**, **DC3-T**, respectively. Discrimination factors of a full-matched sequence to a single mismatch sequence are given in the figures. All reaction was done in conditions: 50 nM of dual coumarin PNAs, 5 nM of **RuUD** and **RuDD**, 1 nM of targets (5 nM for **KRAS-A** and **BCR-A**), 300 ng of single stranded sperm DNA, 1×PBS buffer pH 7.4, no divalent cation, 0.01% tween-20, 5 mM NaAsc, 25 °C. *signal is below the background.

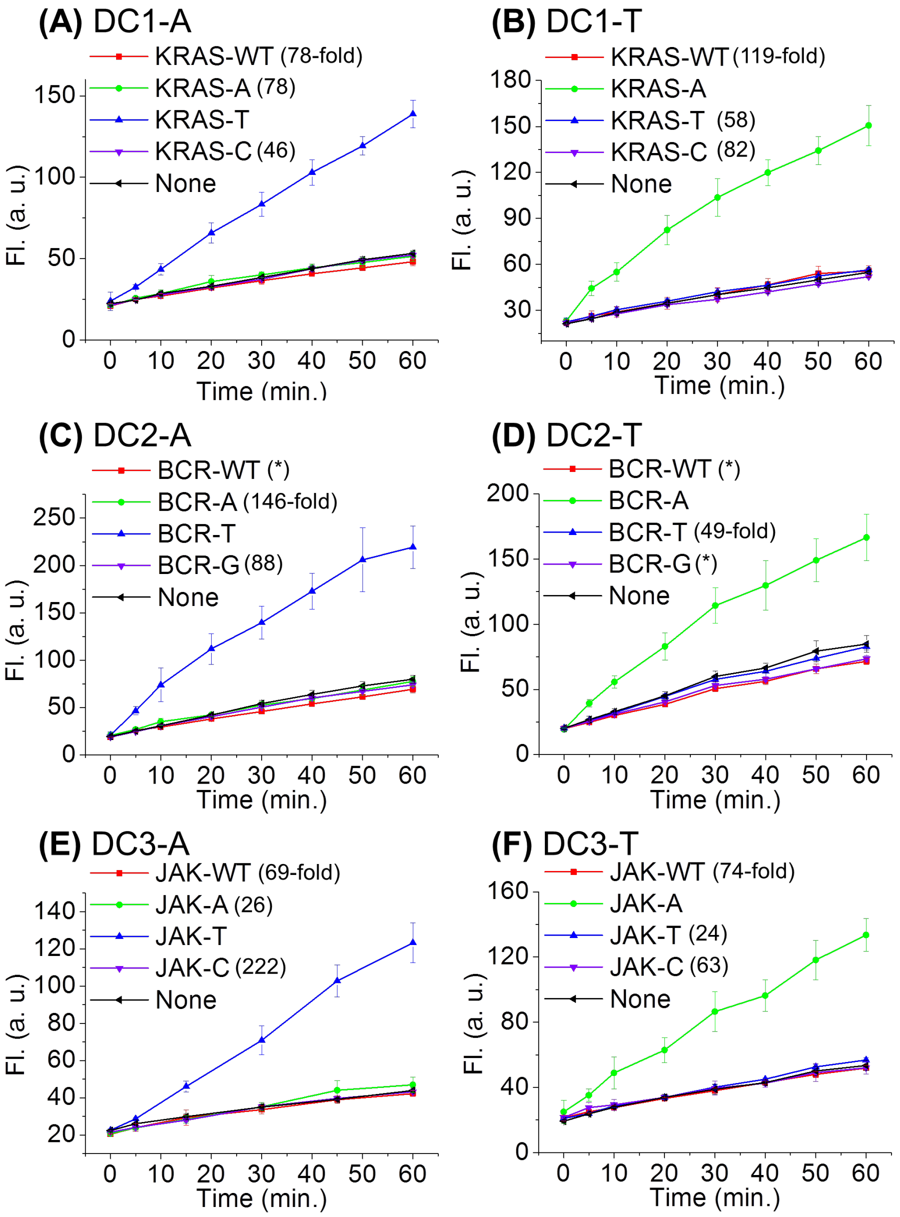

Figure S7. CHOMS templated reaction using **DC1-A**, **RuUD1**, **RuDD1**, and **KRAS-T**. Templated reaction of **DC1-A** was not affected by 50 nM of **DR1** rhodamine PNA, which implies no cross reactivity between two PNAs containing. Conditions: 50 nM of PNAs, 5 nM of **RuUD1**, **RuDD1**, 1 nM of **KRAS-T** for the catalytic condition, 1×PBS buffer pH 7.4, no divalent cation, 0.01% tween-20, 5 mM NaAsc, 25 °C.

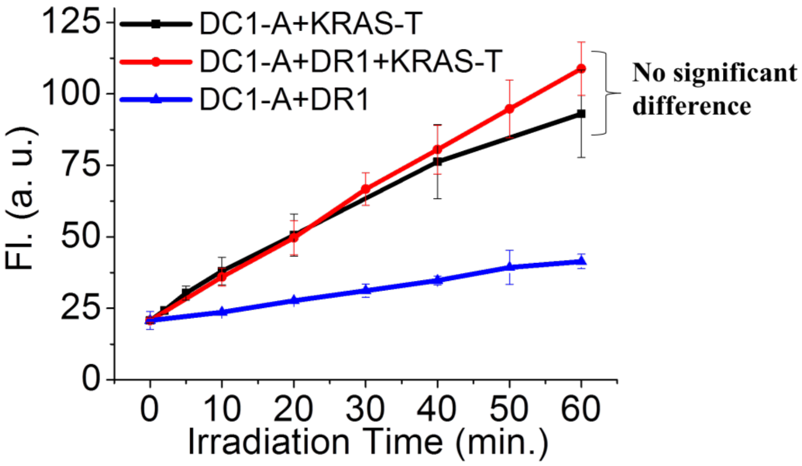

Figure S8. Two-color system of CHOMS templated reactions using 4-mer PNAs releasing rhodamine or coumarin for simultaneous detection of two different wild-type. Conditions: pH 7.4 1×PBS buffer, 0.01% tween-20, 5 mM NaAsc, 25 °C, 50 nM of each coumarin and rhodamine 4-mer PNAs, 5 nM of corresponding RuUD and RuDD for each target (total 20 nM of Ru), 1 nM of target sequences, 300 ng of single stranded sperm DNA. Fluorescence measurement: λ_{exc}: 490 nm, λ_{emi}: 530 nm, cutoff: 515 nm for rhodamine; λ_{exc}: 360 nm, λ_{emi}: 460 nm, cutoff: 455 nm for coumarin.

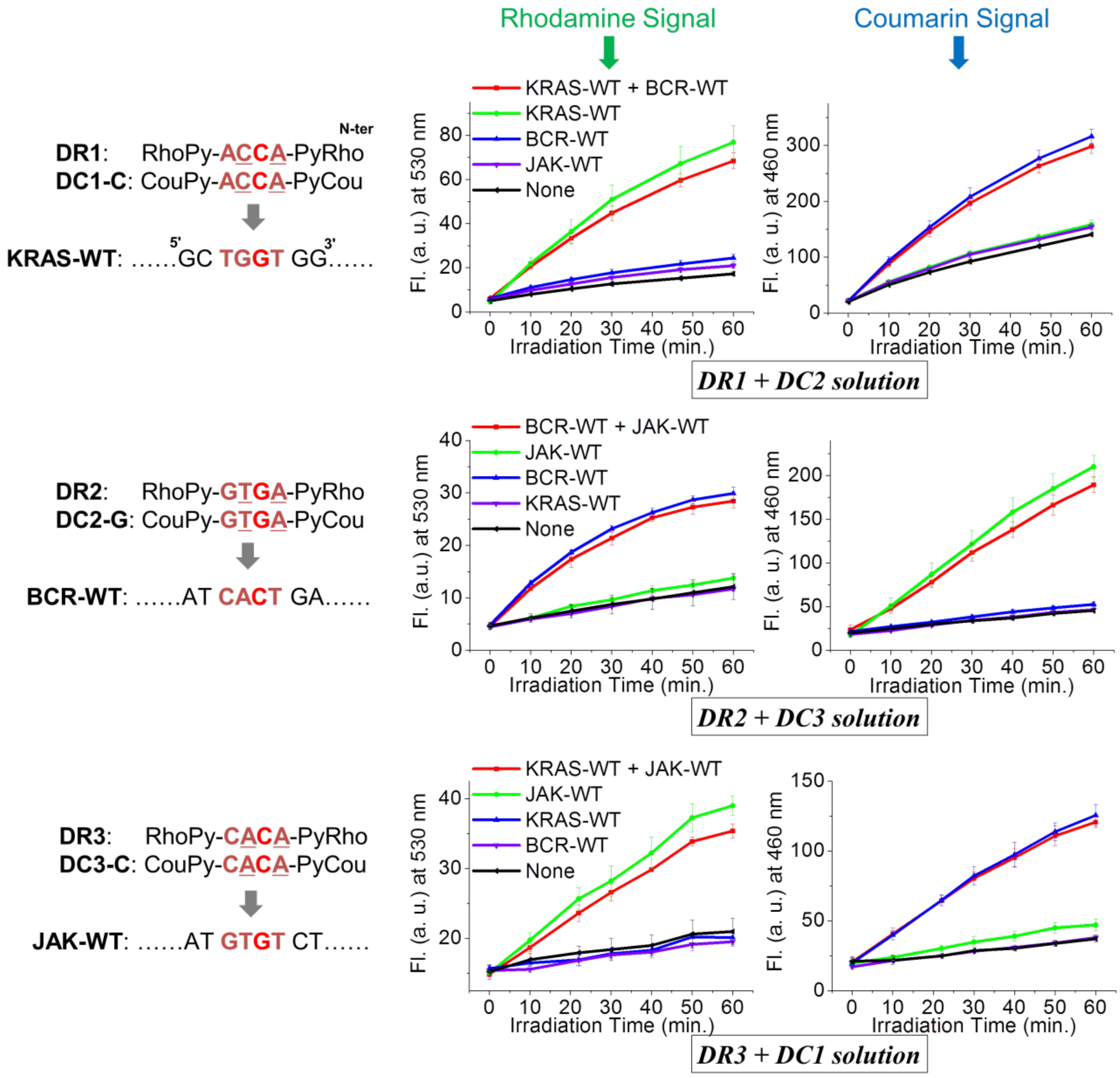

Figure S9. Analysis of PCR products by 10 % 8M urea denaturing polyacrylamide gel electrophoresis. Lane 1: Reverse primer; Lane 2: Forward primer; Lane 3: **BCR-WT** (50 nt); Lane 4: PCR product without cDNA; Lane 5: PCR product of cDNA from A549 cells; Lane 6: PCR product of cDNA from SW620 cells; Lane 7: PCR product of cDNA from HT-29 cells; Lane 8: 100 bp DNA ladder. Gel was stained by 2XSYBR Gold for 30 min. cDNA of each cell line produced identical strong PCR bands (expected to be 71-mer) observed in between 50 nt and 100 bp while no product was found in control PCR (lane 4).

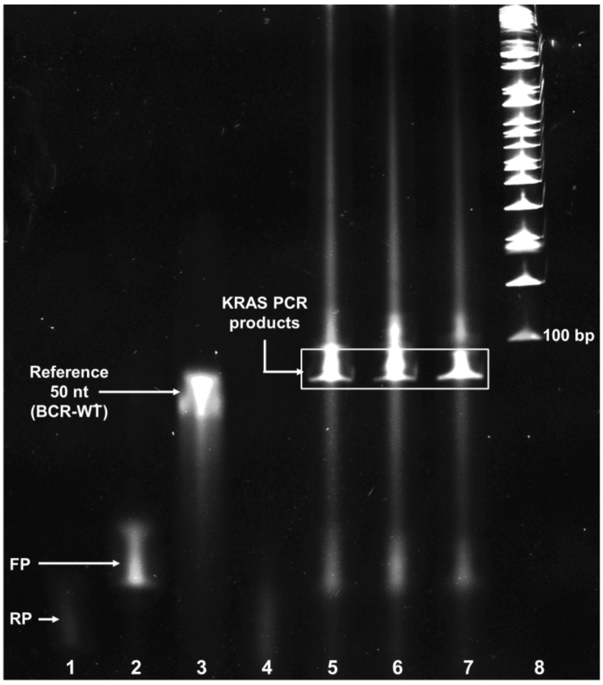

Figure S10. Two-color CHOMS templated reaction using RT-PCR products of total RNA extracted from cell lines, HT-29, SW620, and A549. (a) Rhodamine fluorescence signal from **DR1**, (b) coumarin fluorescence signal from **DC1-A**, (c) coumarin fluorescence signal from **DC1-34A** were monitored in the prescence of RT-PCR product of HT-29, SW620, A549 cell lines. No signal was observed for non-targeted reaction. Conditions for the two-color system: pH 7.4 1×PBS buffer, 0.01% tween-20, 5 mM NaAsc, 25 °C, 50 nM of **DR1**, 50 nM of **DC1-A** for (a and b) or **DC1-34A** for (c), 5 nM of each **RuUD1** and **RuDD1**, 20 μL of PCR sample, total 200 μL.

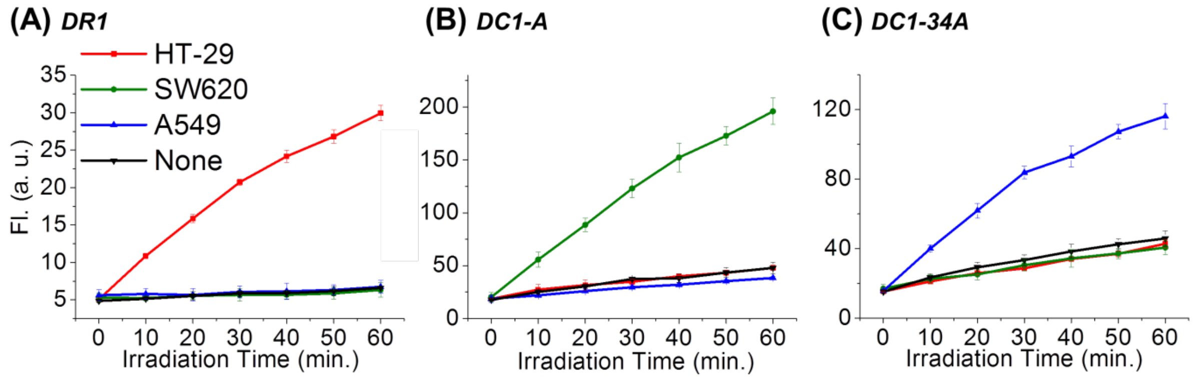

Table S2. Discrimination factor and two sample unpaired t-test with unequal variances (p-value) between target and single mismatch sequences. The target signal was below background (none) signal for #16, 19, 21. The discrimination factor is calculated as follow: (fluorescence of target-background)/(fluorescence of mismatch-background); The two sample t-test was calculated from raw fluorescence value using ORIGIN software. The discrimination for two color is calculated as follow: (Rh-fluorescence of target-background)/(Rh-fluorescence of mismatch-background)*(Coumarin-fluorescence of target-background)/(Coumarin-fluorescence of mismatch-background)

#	Target (probe)	Mismatch	Discrimination Factor	Two-sample t-test (p-value)
1	KRAS-WT (DC1-C)	KRAS-A	123	0.001874
2		KRAS-T	92	0.00134
3		KRAS-C	303	0.002705
4	BCR-WT (DC2-G)	BCR-A	309	0.001778
5		BCR-T	90	0.000958
6		BCR-G	135	0.000394
7	JAK-WT (DC3-C)	JAK-A	163	0.002386
8		JAK-T	81	0.002363
9		JAK-C	44	0.001905
10	KRAS-T (DC1-A)	KRAS-WT	78	0.001344
11		KRAS-A	78	0.000404
12		KRAS-C	46	0.002246
13	KRAS-A (DC1-T)	KRAS-WT	119	0.000548
14		KRAS-T	58	4.22E-05
15		KRAS-C	82	0.000174
16	BCR-T (DC2-A)	BCR-WT	Below background	0.008417
17		BCR-A	146	0.008506
18		BCR-G	88	0.009496
19	BCR-A (DC2-T)	BCR-WT	Below background	0.01388
20		BCR-T	49	0.017829
21		BCR-G	Below background	0.015376
22	JAK-T (DC3-A)	JAK-WT	69	0.004959
23		JAK-A	26	0.002621
24		JAK-C	222	0.005534
25	JAK-A (DC3-T)	JAK-WT	72	0.005211
26		JAK-T	24	0.006021
27		JAK-C	63	0.003971
28	KRAS-WT (DR1) in two-color	KRAS-T	1022	1.57E-07
29	BCR-WT (DR2) in two-color	BCR-T	1113	2.92E-05
30	JAK-WT (DR3) in two-color	JAK-T	3148	4.17E-07

3. Mass spectra of the synthesized DNAs and PNAs. Inserts show the theoretical isotopic distribution of the exact mass calculation.

RuUD1 (Ru-GCTCCAACTACCACAAGT); Exact Mass for $C_{216}H_{264}N_{76}O_{108}P_{18}Ru^{2+}$: 6309.1823, (negative) MALDI-TOF m/z found: 6308.807 $[M-3H]^{-}$, 3154.819 $[M-4H]^{2-}$

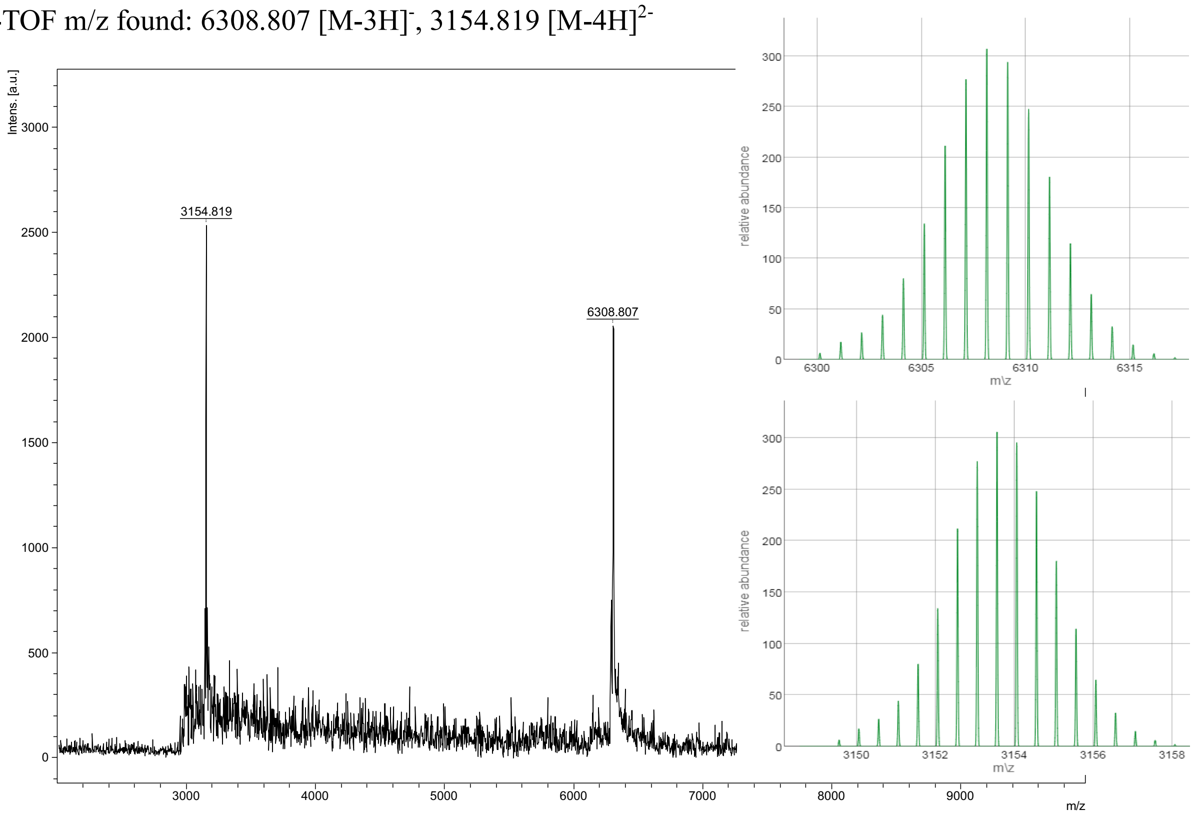

RuDD1 (GGCACTCTTGCCTACGCC-Ru); Exact Mass for $C_{216}H_{267}N_{71}O_{114}P_{18}Ru^{2+}$: 6338.1599, (negative) MALDI-TOF m/z found: 6337.986 $[M-3H]^{-}$, 3167.948 $[M-4H]^{2-}$

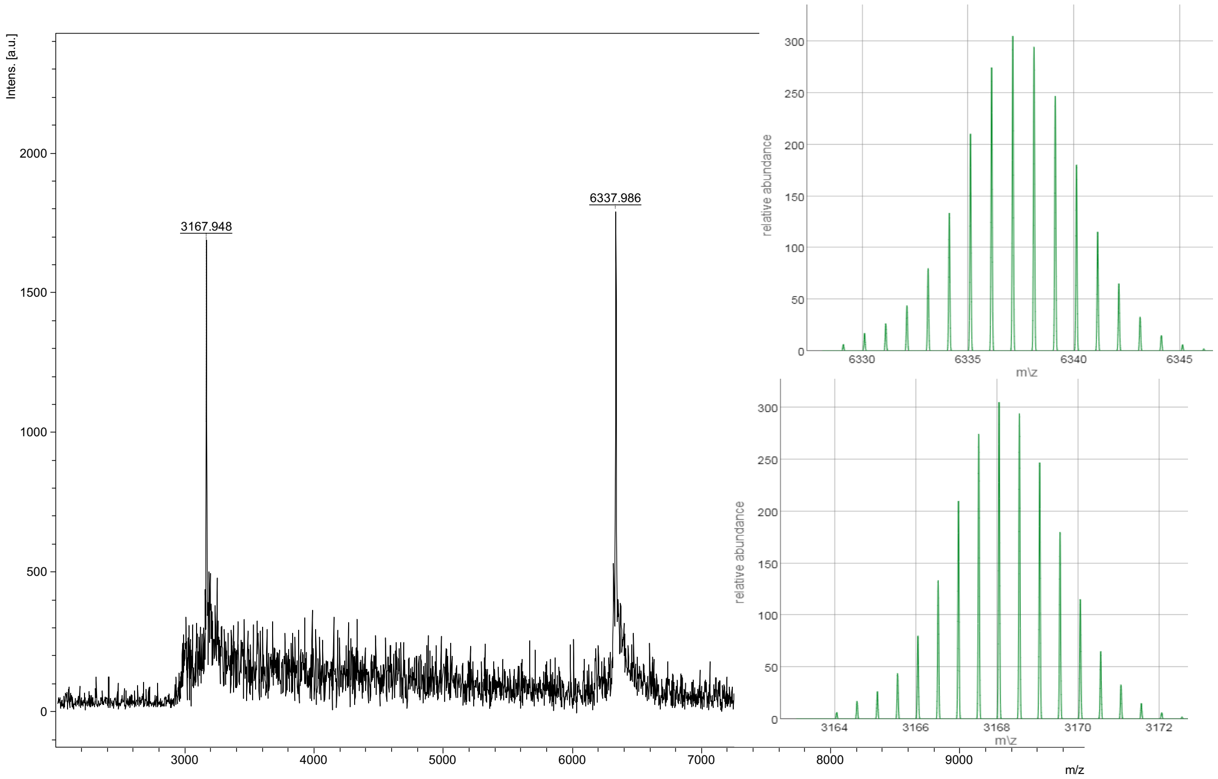

RuUD-s (Ru-CTCCAACTACCACAAGT); Exact Mass for $C_{206}H_{252}N_{71}O_{102}P_{17}Ru^{2+}$: 5980.1297, (negative) MALDI-TOF m/z found: 5979.746 $[M-3H]^{-}$

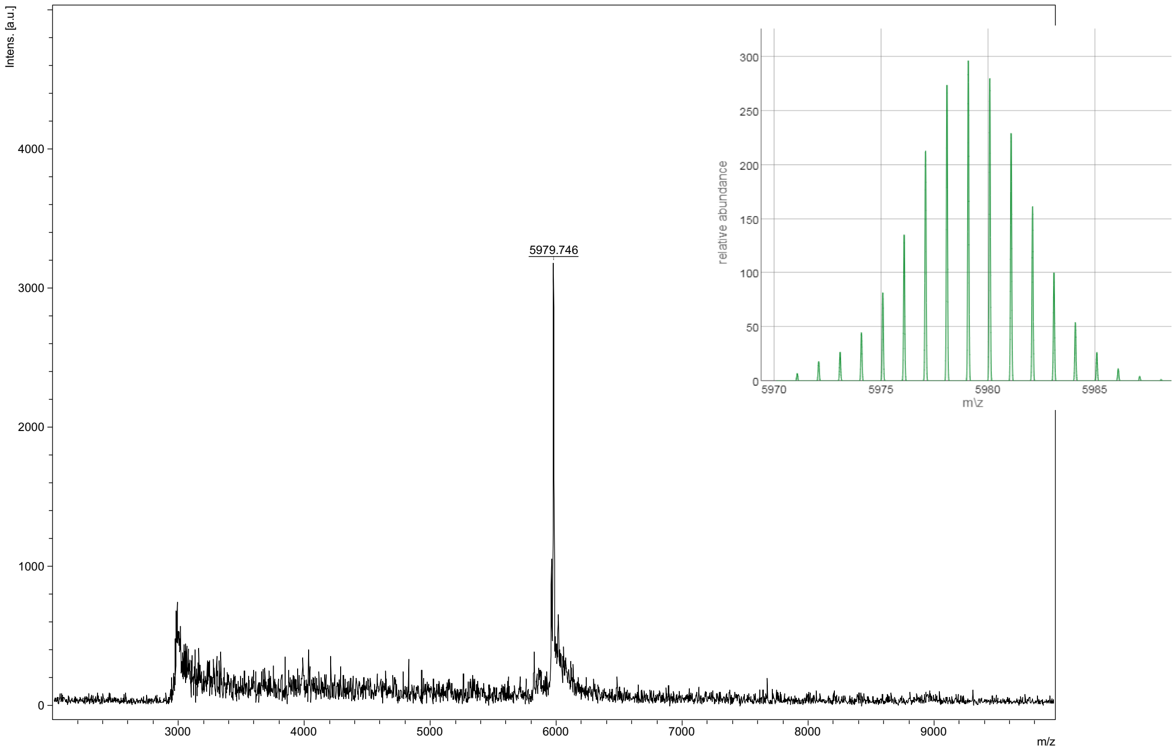

RuDD-s (CCTACGCCACCAGCTCCA-Ru); Exact Mass for $C_{214}H_{265}N_{73}O_{110}P_{18}Ru^{2+}$: 6276.1707, (negative) MALDI-TOF m/z found: 6276.297 $[M\text{-}3H]^{-}$, 3137.978 $[M\text{-}4H]^{2-}$

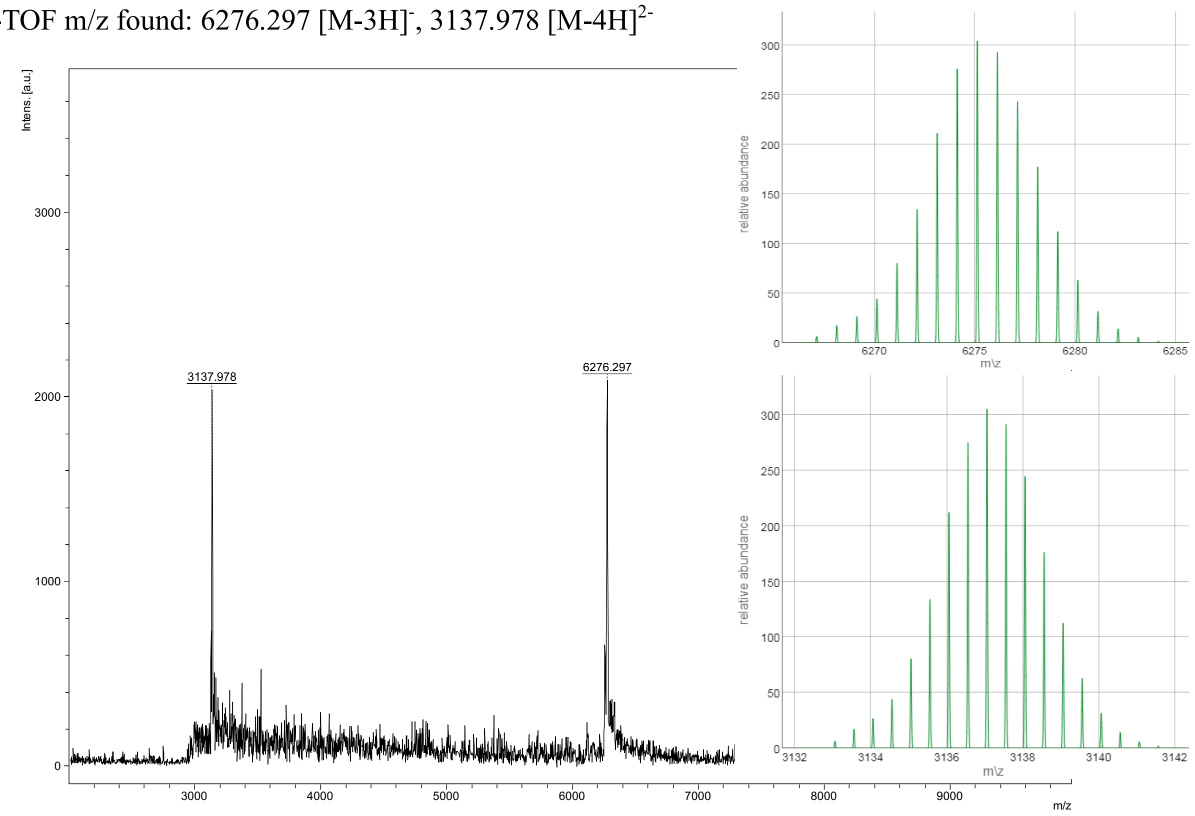

RuUD-3M (Ru-AGCTCCAACTACCACAAG); Exact Mass for $C_{216}H_{263}N_{79}O_{106}P_{18}Ru^{2+}$: 6318.1938, (negative) MALDI-TOF m/z found: 6319.410 $[M\text{-}3H]^{-}$, 3159.371 $[M\text{-}4H]^{2-}$

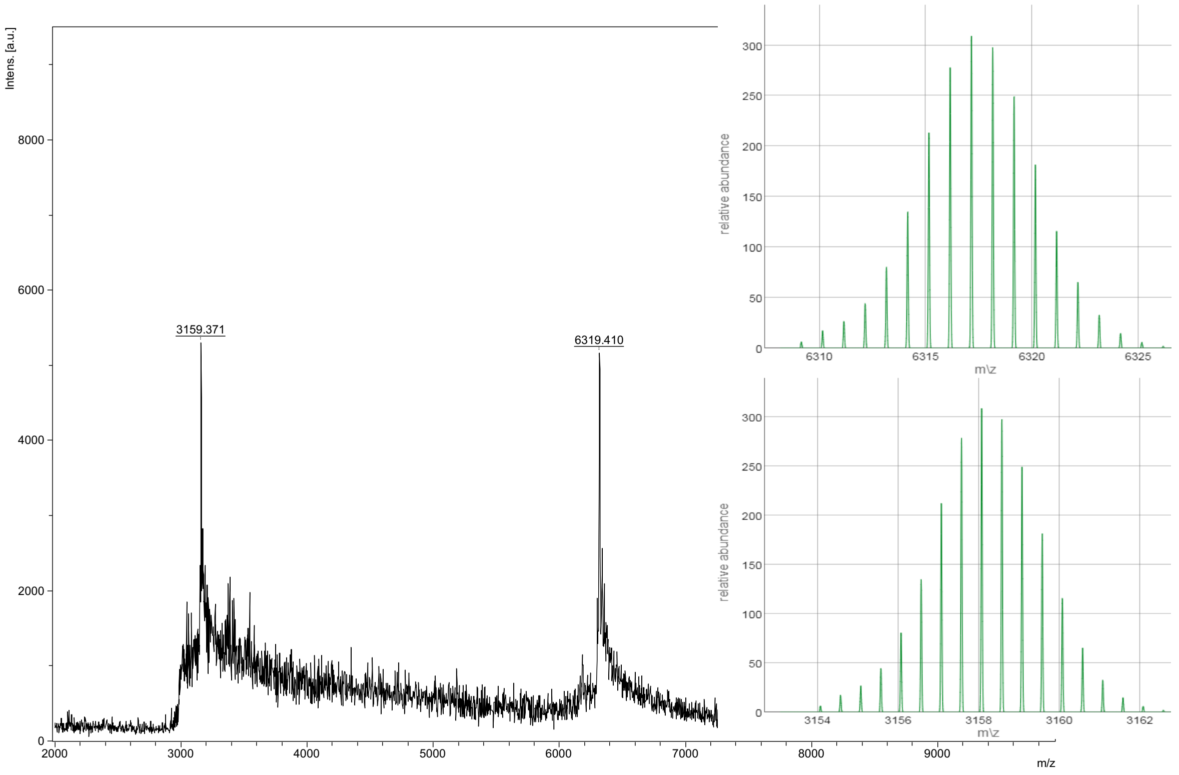

RuUD2 (Ru-ATGATATAGAACGGGGGC); Exact Mass for $C_{221}H_{264}N_{86}O_{108}P_{18}Ru^{2+}$: 6509.2130, (negative) MALDI-TOF m/z found: 6510.564 $[M-3H]^{-}$

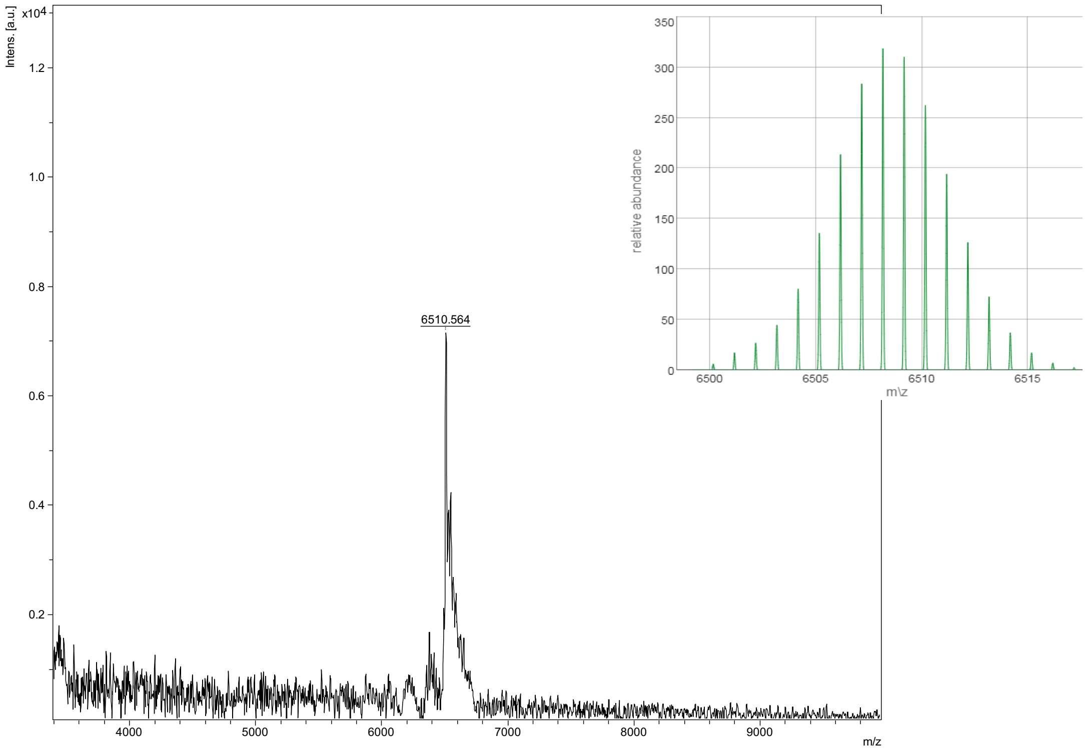

RuDD2 (CGTAGGTCATGAACTC-Ru); Exact Mass for $C_{200}H_{243}N_{69}O_{100}P_{16}Ru^{2+}$: 5808.0896, (negative) MALDI-TOF m/z found: 5807.997 $[M-3H]^{-}$

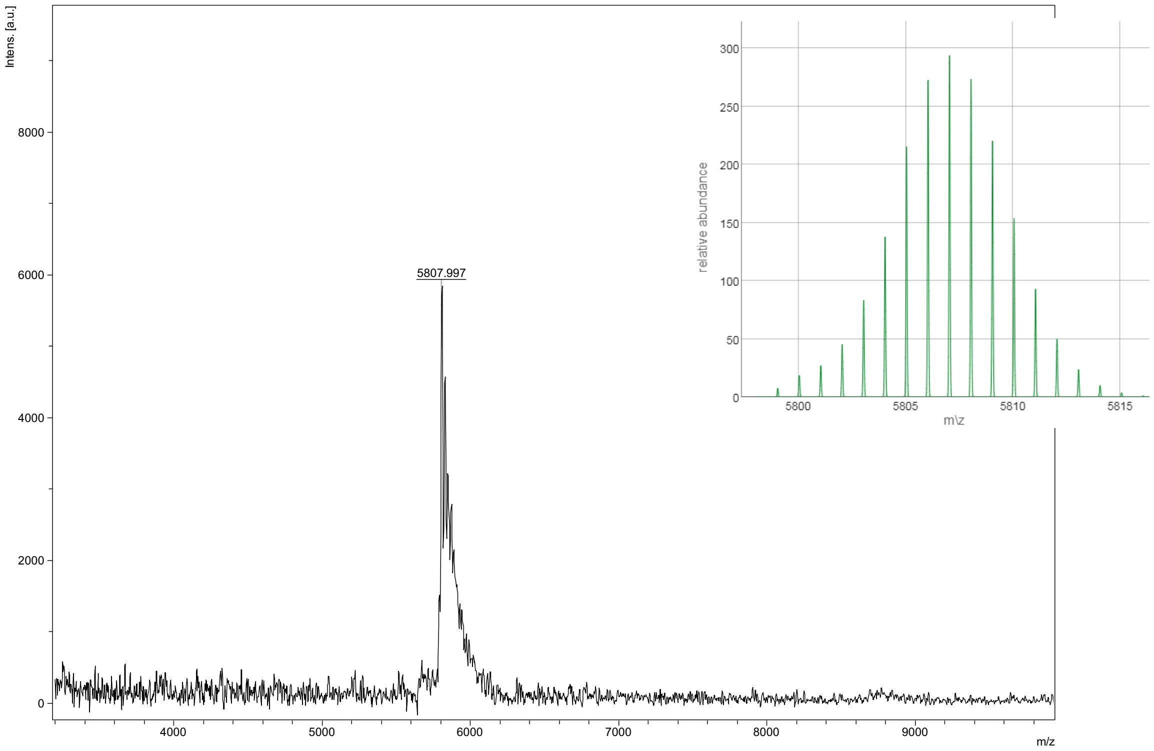

RuUD3 (Ru-ATACTCCATAATTTAAAA); Exact Mass for $C_{220}H_{267}N_{75}O_{108}P_{18}Ru^{2+}$: 6346.2027, (negative) MALDI-TOF m/z found: 6346.770 $[M-3H]^{-}$, 3173.496 $[M-4H]^{2-}$

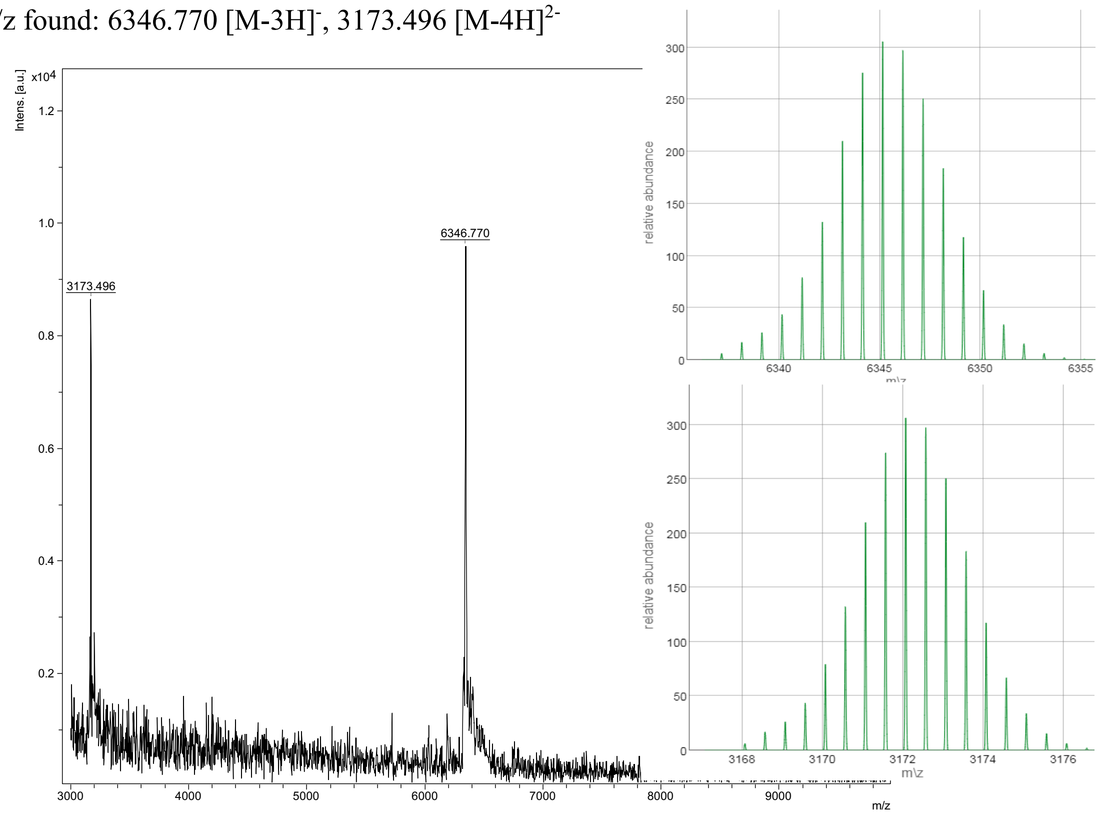

RuDD3 (ATTCTCGTCTCCACAG-Ru); Exact Mass for $C_{198}H_{244}N_{62}O_{102}P_{16}Ru^{2+}$: 5719.0657, (negative) MALDI-TOF m/z found: 5718.805 $[M-3H]^{-}$

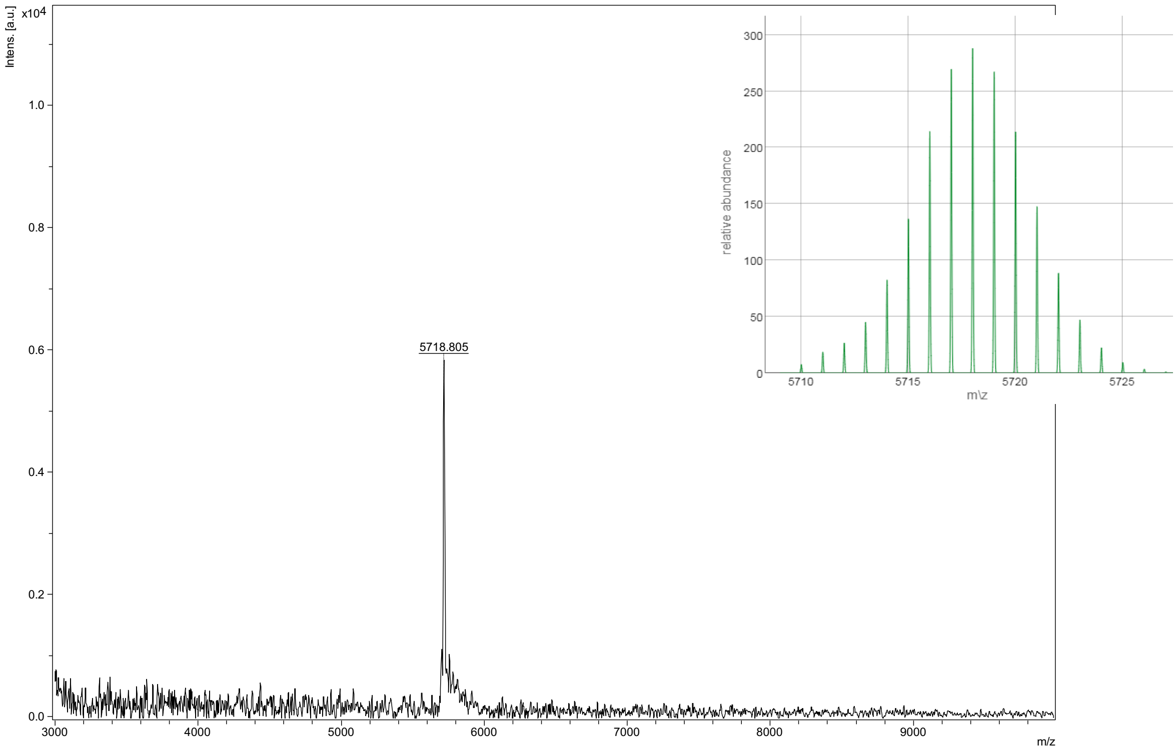

SC1-A (CouPy-Lys-A̲AC̲A); Exact Mass for $C_{77}H_{94}F_2N_{33}O_{17}^{+}$: 1790.7474, LC-MS (ESI) RT= 1.38 min. m/z: 1791.5 $[M]^{+}$, 1194.25 $[2M+H]^{3+}$, 896.33 $[M+H]^{2+}$, 597.92 $[M+2H]^{3+}$, 448.58 $[M+3H]^{4+}$; (positive) MALDI-TOF m/z found: 1581.15 [M-Coumarin]$^{+}$.

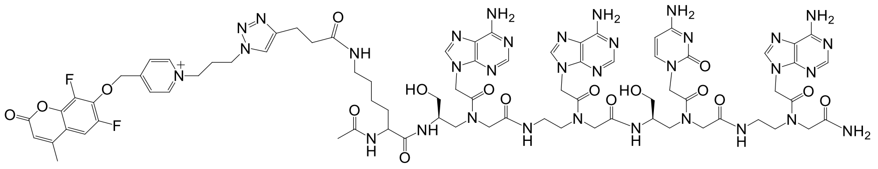

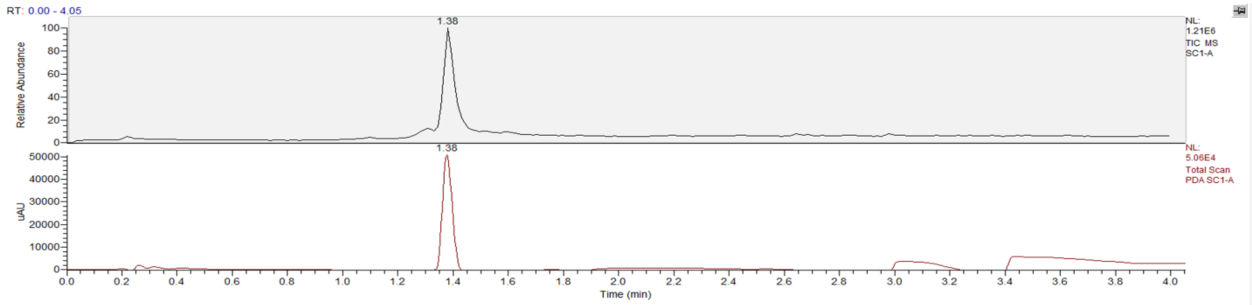

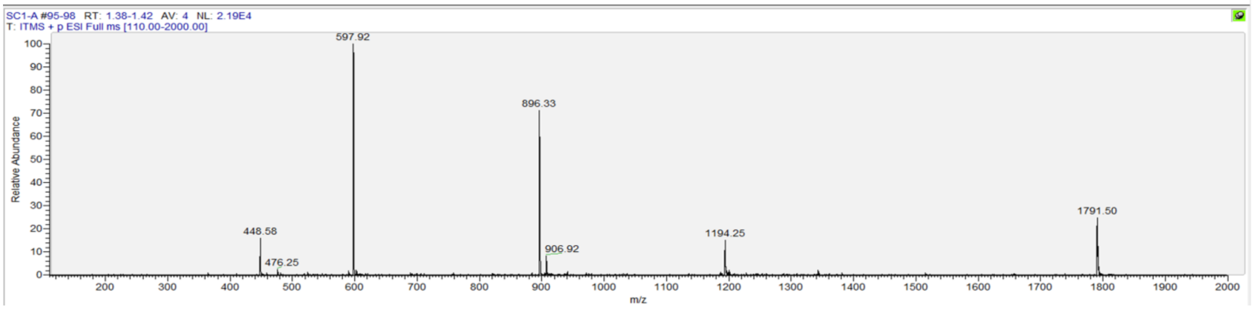

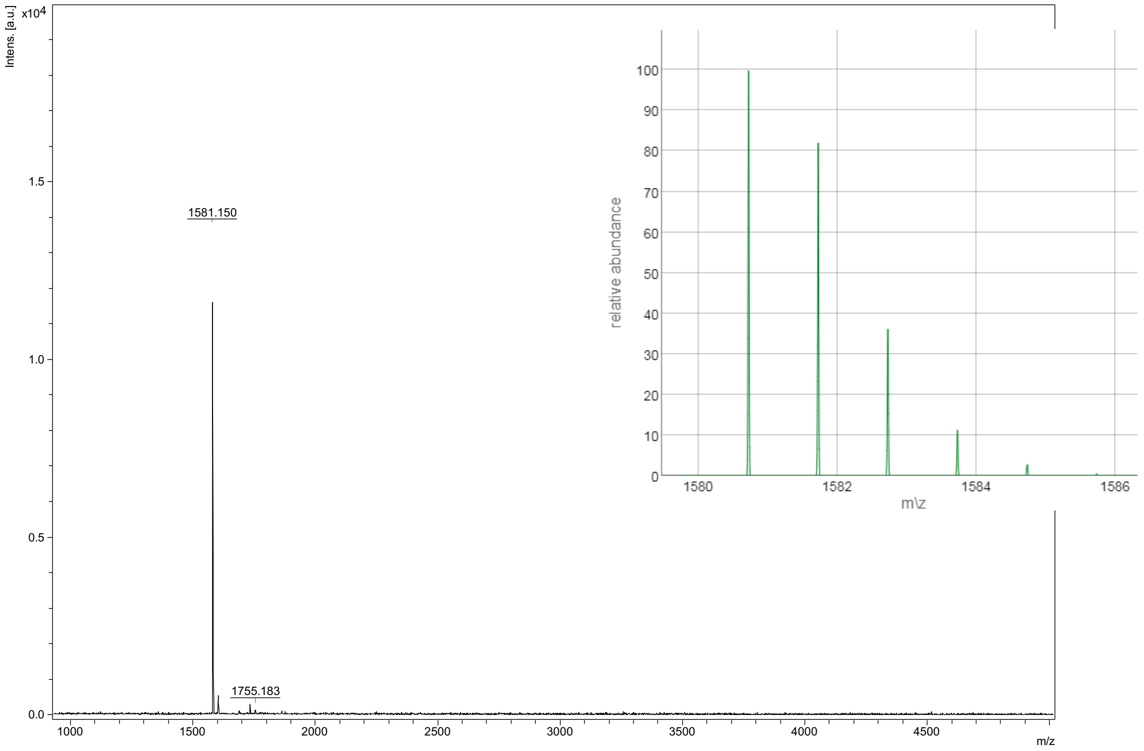

DC1-C (CouPy-Lys-ACCA-Lys-PyCou); Exact Mass for $C_{106}H_{127}F_4N_{37}O_{23}^{2+}$: 2361.9836, LC-MS (ESI) RT= 1.60 min. m/z: 1181.50 $[M]^{2+}$, 788.08 $[M+H]^{3+}$, 591.25 $[M+2H]^{4+}$; (positive) MALDI-TOF m/z found: 1941.101 [M-H-2 • Coumarin]$^{+}$.

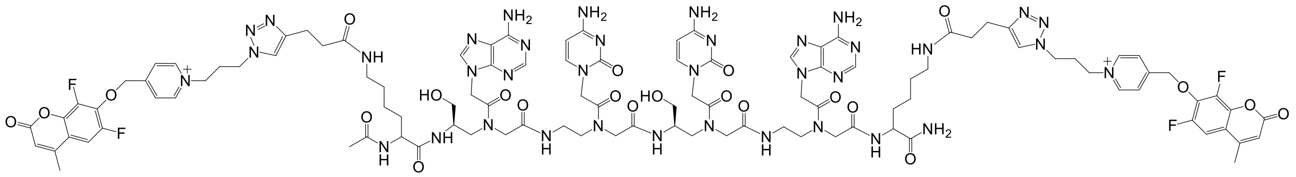

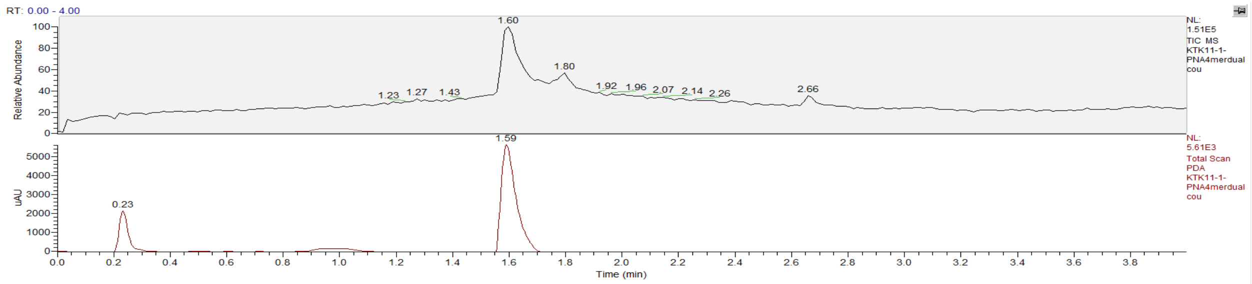

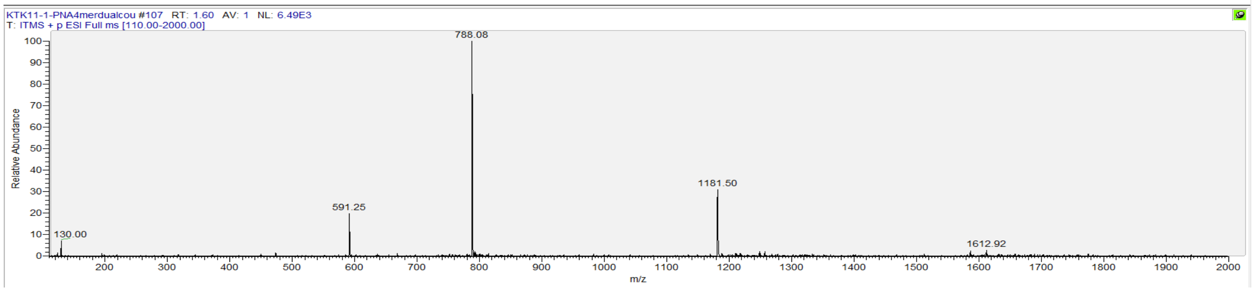

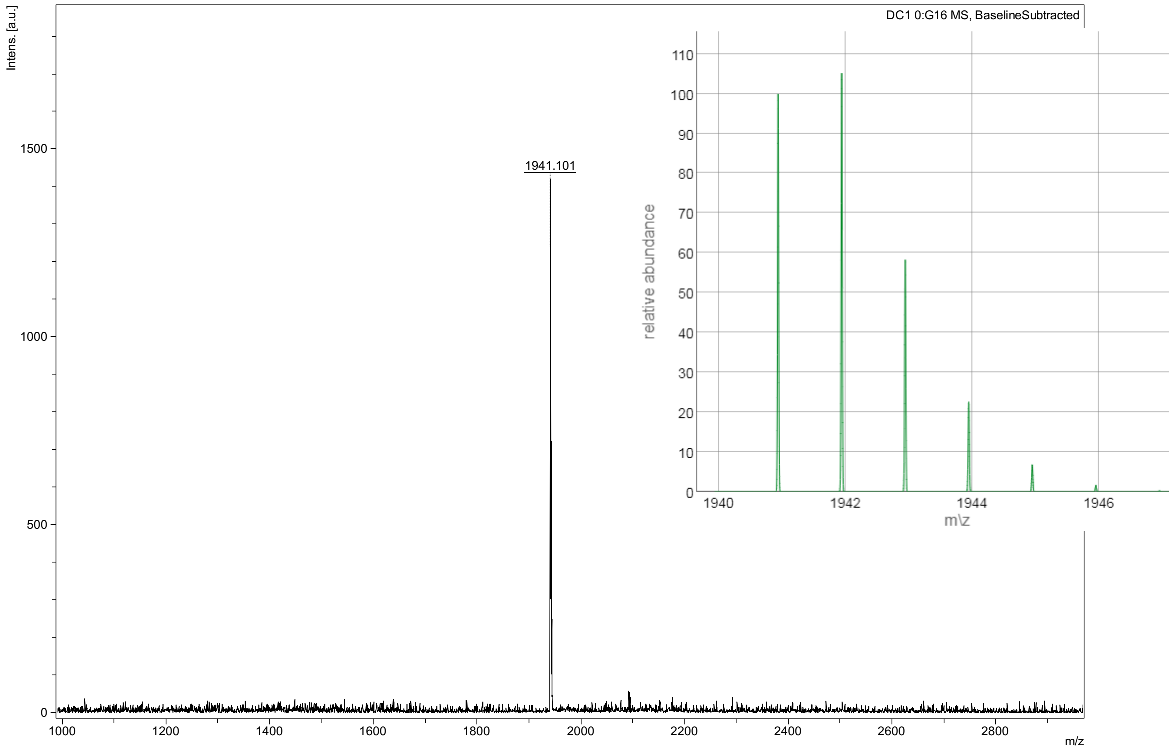

SC-8 (CGCCACCA-Lys-PyCou); Exact Mass for $C_{119}H_{150}F_2N_{53}O_{32}^+$: 2871.1708, LC-MS (ESI) RT= 1.32 min. m/z: 1436.67 $[M+H]^{2+}$, 958.25 $[M+2H]^{3+}$, 719.00 $[M+3H]^{4+}$, 575.67 $[M+4H]^{5+}$; (positive) MALDI-TOF m/z found: 2661.372 $[M\text{-Coumarin}]^+$.

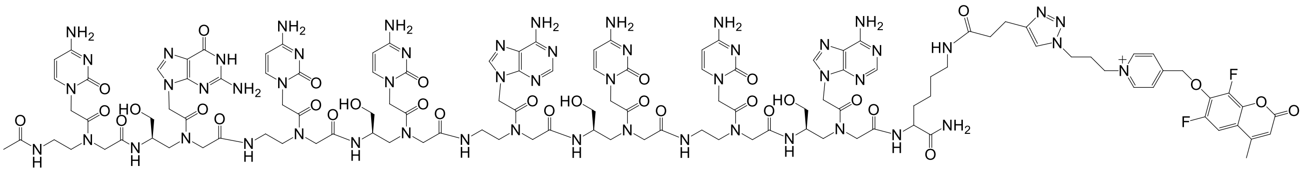

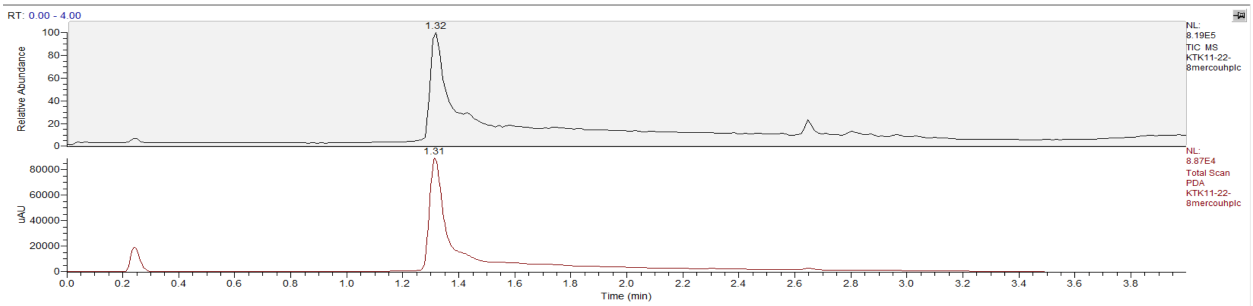

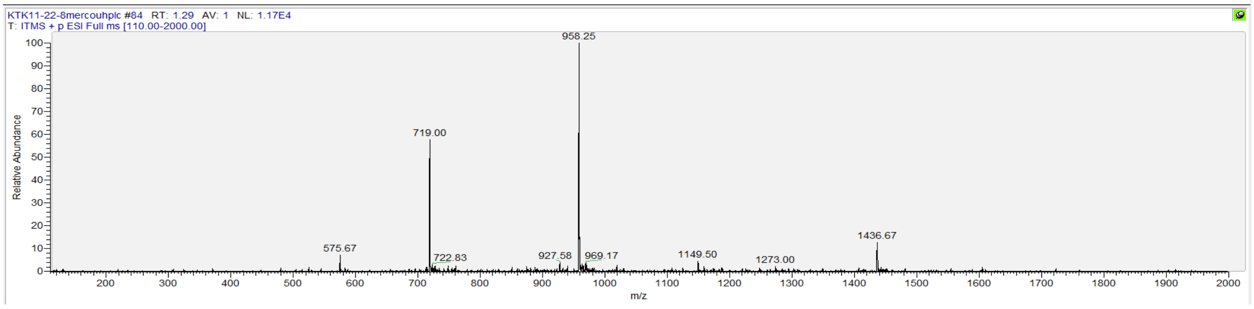

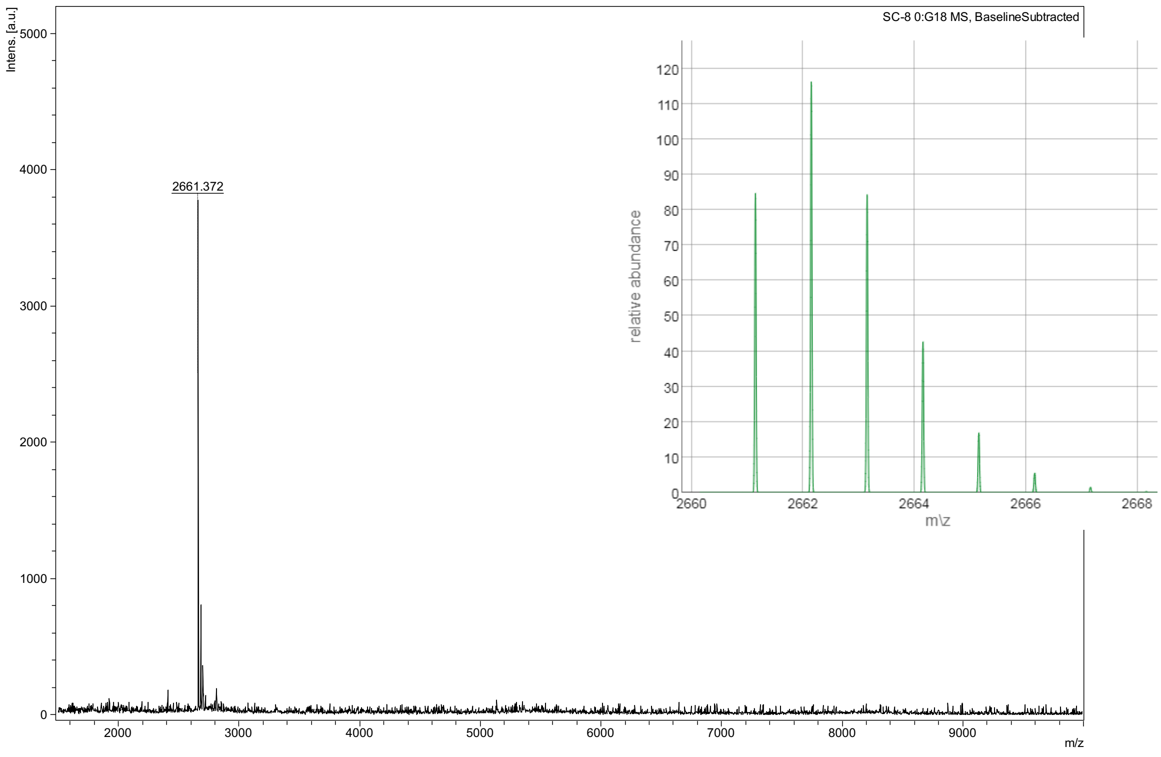

RuUP1 (Ru-Lys-GCTCCAACTACC); Exact Mass for $C_{177}H_{217}N_{77}O_{45}Ru^{2+}$: 4242.6102, LC-MS (ESI) RT= 1.27 min. m/z: 1414.5 $[M+H]^{3+}$, 1061.5 $[M+2H]^{4+}$, 849.67 $[M+3H]^{5+}$, 708.08 $[M+4H]^{6+}$; 607.17 $[M+5H]^{7+}$; 531.50 $[M+6H]^{8+}$; 472.58 $[M+7H]^{9+}$; (positive) MALDI-TOF m/z found: 4241.999 $[M-H]^{+}$.

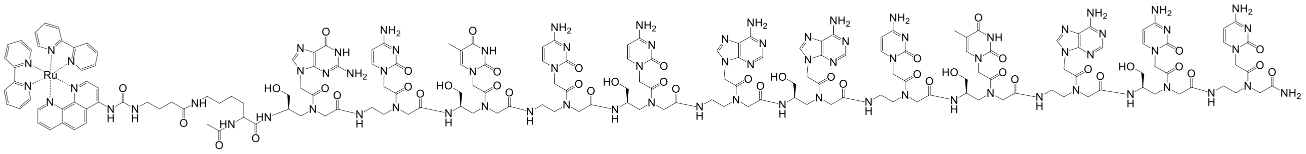

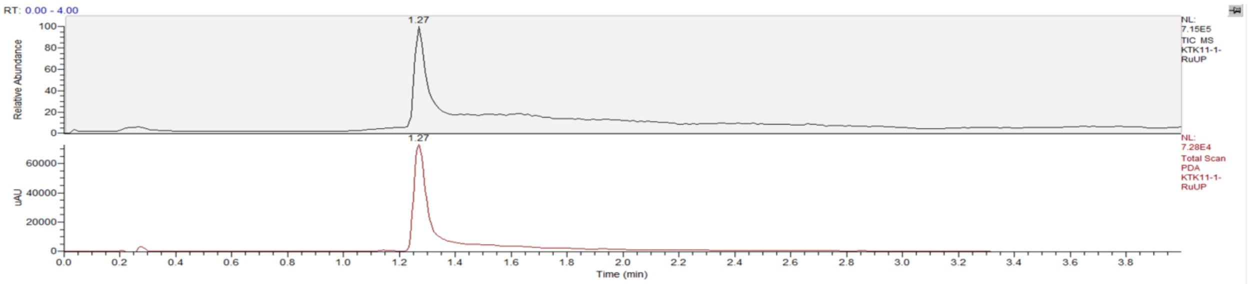

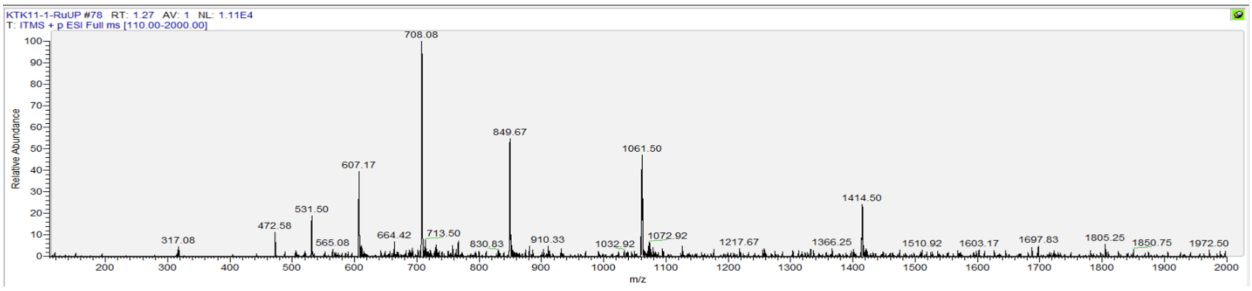

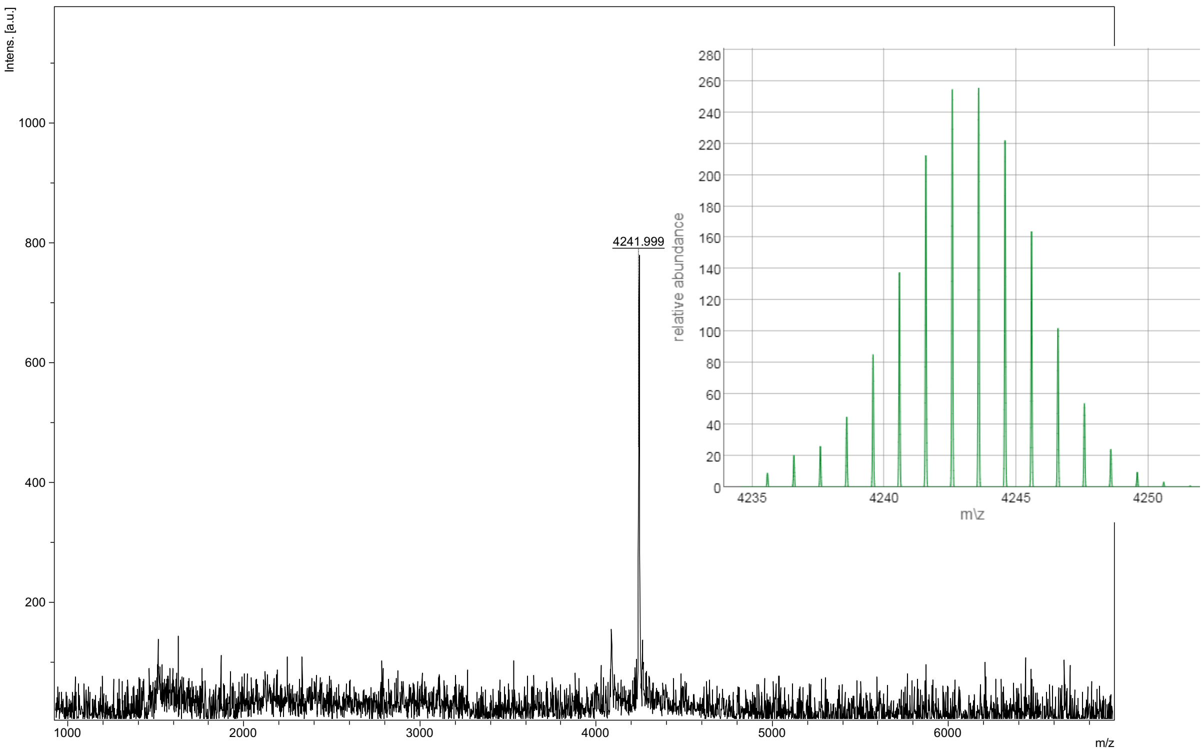

RuDP1 (Ac-CTTGCCTACGCC-Lys-peg-Ru); Exact Mass for $C_{183}H_{228}N_{75}O_{51}Ru^{2+}$: 4393.6597, LC-MS (ESI) RT= 1.33 min. m/z: 1466.17 $[M+H]^{3+}$, 1099.67 $[M+2H]^{4+}$, 880.00 $[M+3H]^{5+}$, 733.50 $[M+4H]^{6+}$; 629.08 $[M+5H]^{7+}$; (positive) MALDI-TOF m/z found: 4392.5 $[M-H]^{+}$.

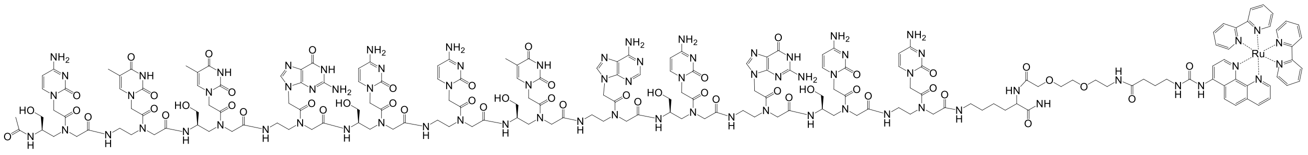

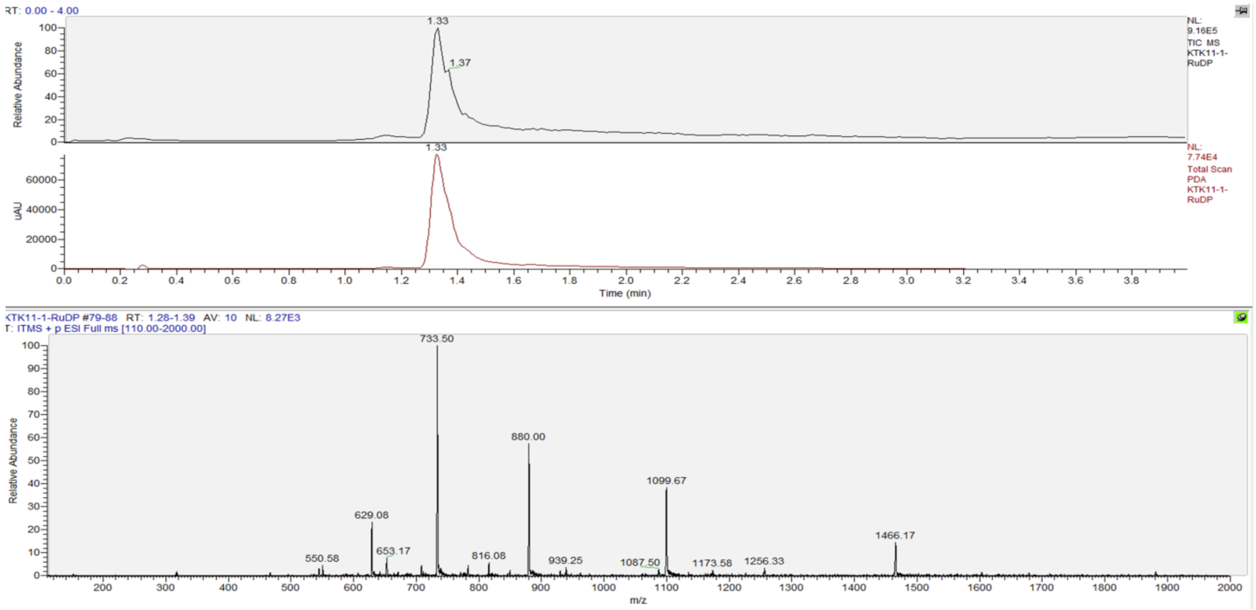

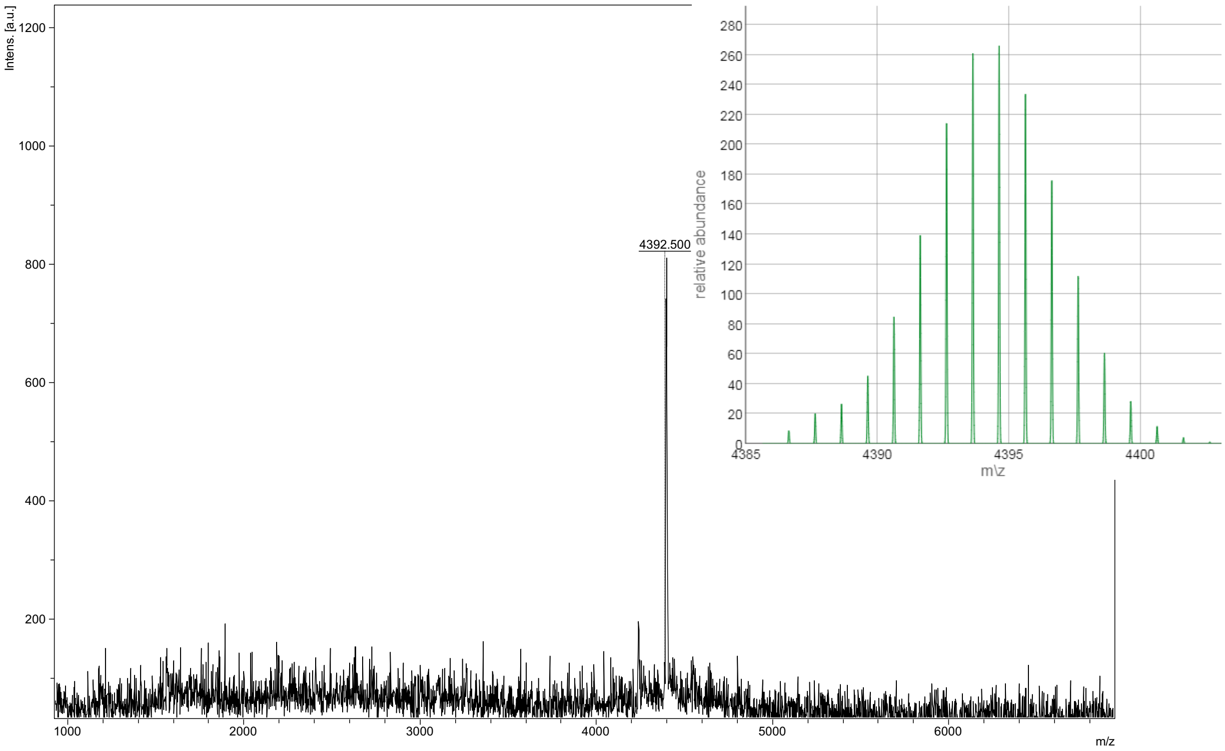

DC-3M (CouPy-Lys-ACC-Lys-PyCou); Exact Mass for $C_{95}H_{114}F_4N_{30}O_{21}^{2+}$: 2086.8711, LC-MS (ESI) RT= 1.56 min. m/z: 1043.5 $[M]^{2+}$, 696.33 $[M+H]^{3+}$, 522.33 $[M+2H]^{4+}$; (positive) MALDI-TOF m/z found: 1666.033 $[M\text{-}H\text{-}2 \bullet \text{Coumarin}]^{+}$.

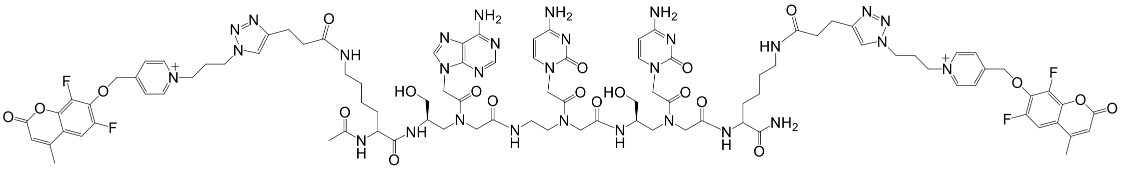

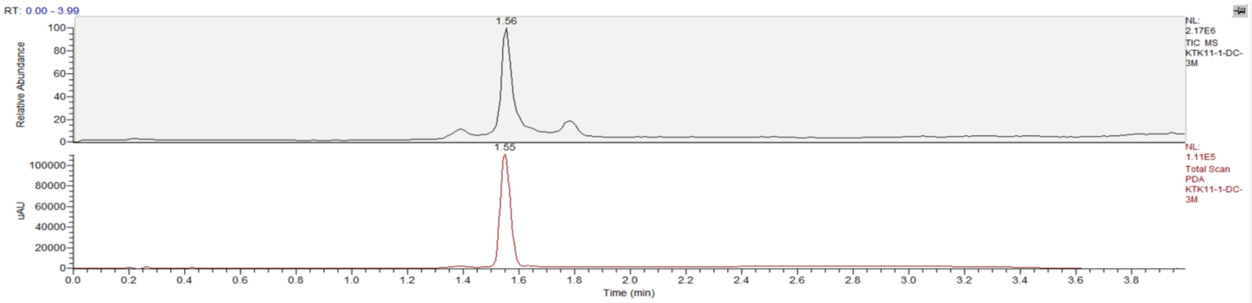

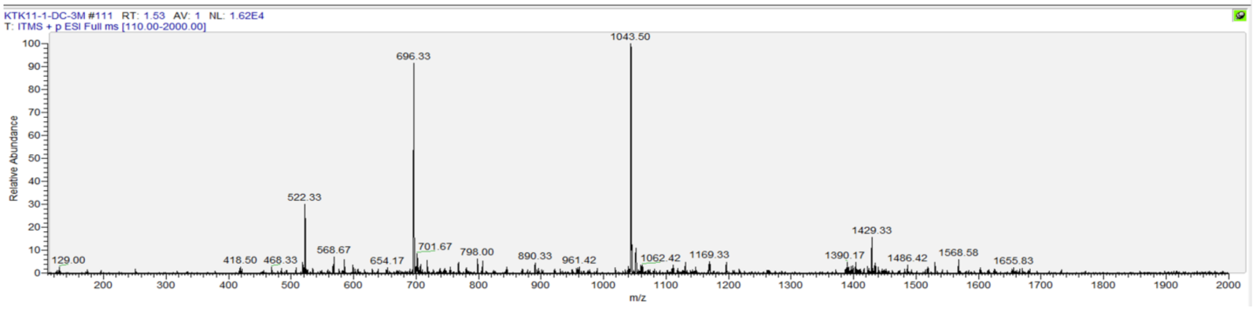

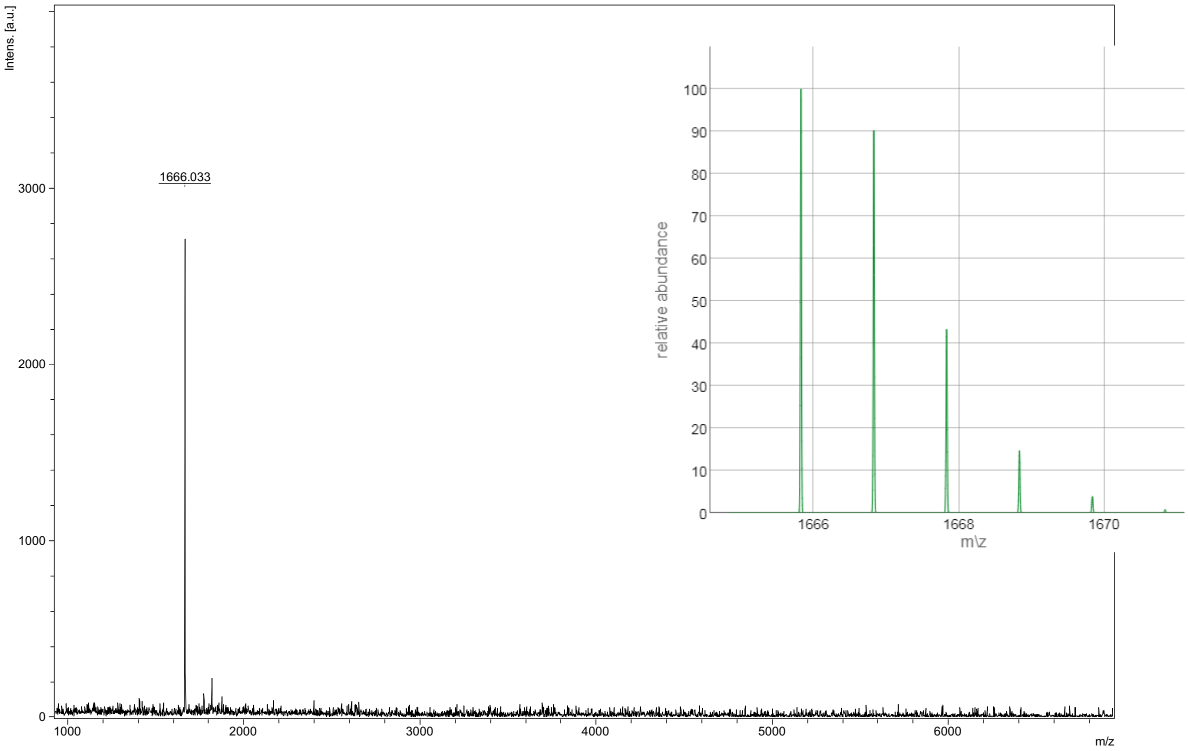

DC2-G (CouPy-Lys-AGTG-Lys-PyCou); Exact Mass for $C_{108}H_{128}F_4N_{38}O_{25}^{2+}$: 2432.9848, LC-MS (ESI) RT= 1.58 min. m/z: 1216.92 $[M]^{2+}$, 811.67 $[M+H]^{3+}$, 609.08 $[M+2H]^{4+}$, 487.25 $[M+3H]^{5+}$; (positive) MALDI-TOF m/z found: 2012.121 [M-H-2 • Coumarin]$^{+}$.

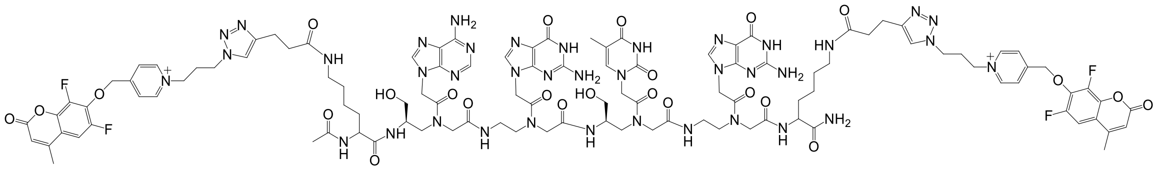

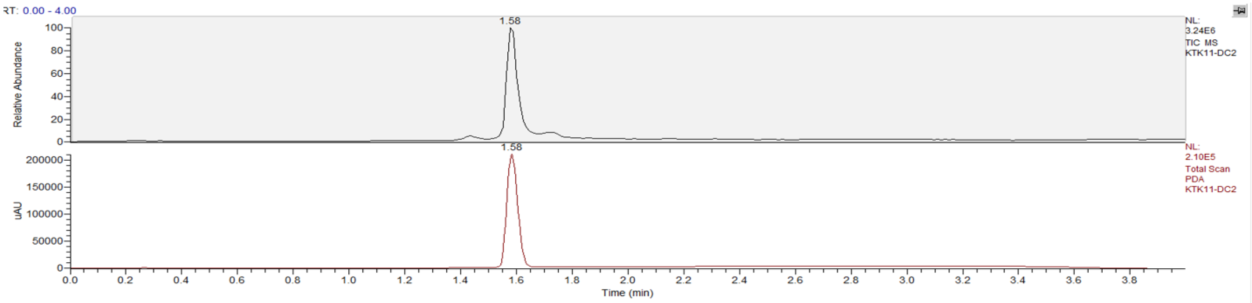

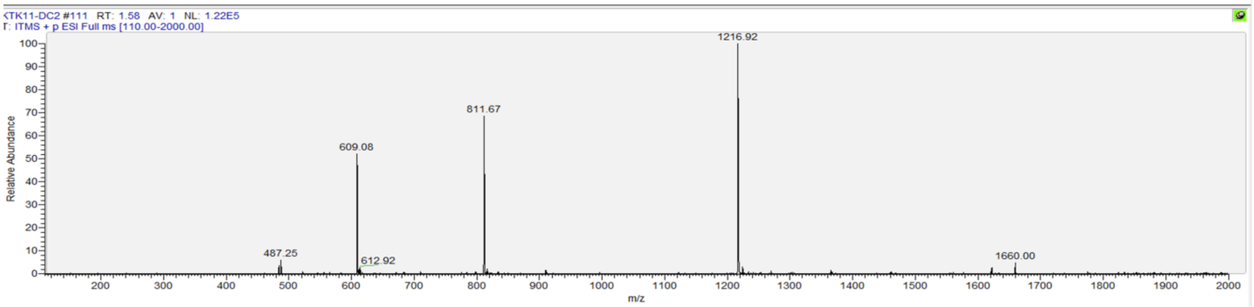

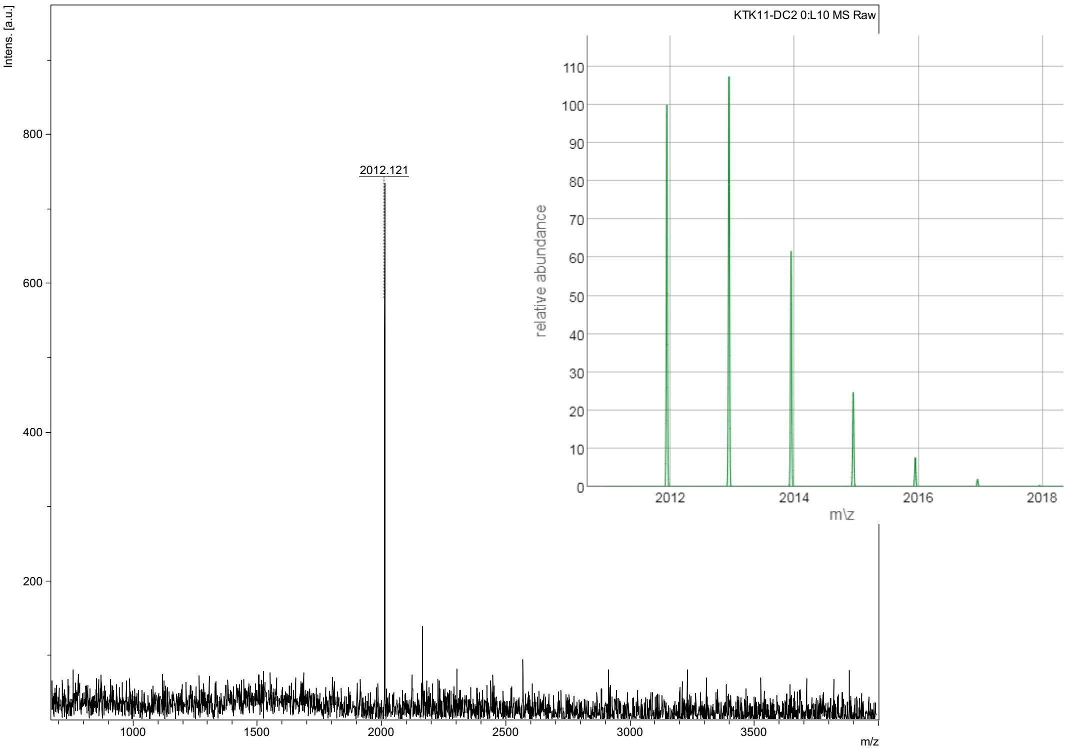

DC3-C (CouPy-Lys-ACAC-Lys-PyCou); Exact Mass for $C_{106}H_{127}F_4N_{37}O_{23}^{2+}$: 2361.9842, LC-MS (ESI) RT= 1.54 min. m/z: 1181.42 $[M]^{2+}$, 788.00 $[M+H]^{3+}$, 591.33 $[M+2H]^{4+}$, 473.17 $[M+3H]^{5+}$; (positive) MALDI-TOF m/z found: 1941.134 [M-H-2 • Coumarin]$^{+}$.

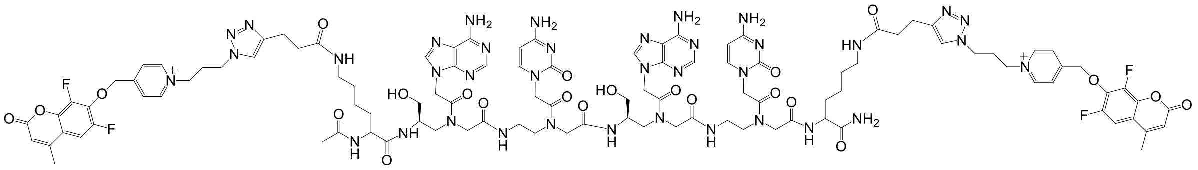

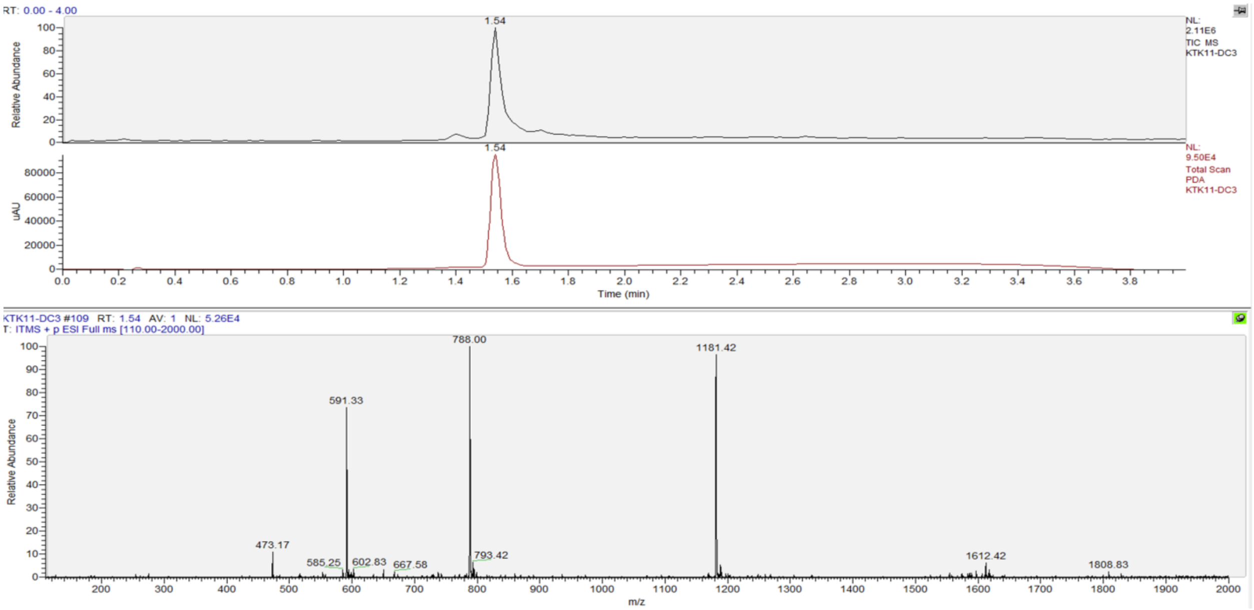

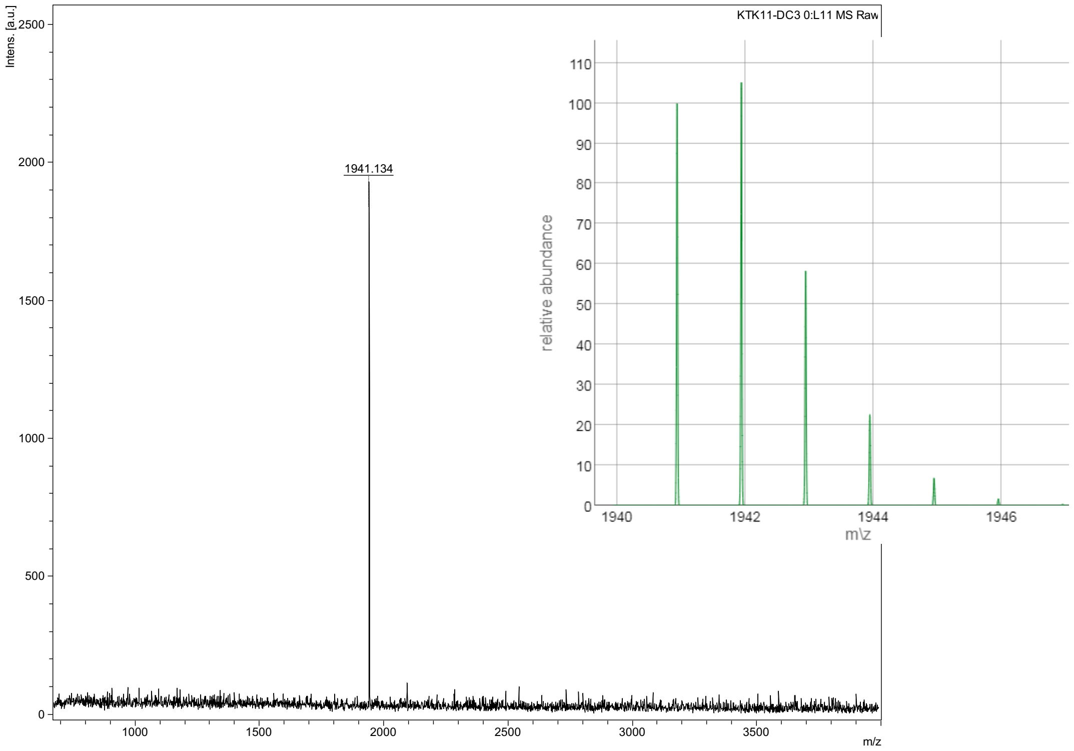

DR1 (RhoPy-Lys-ACCA-Lys-PyRho); Exact Mass for $C_{132}H_{149}N_{43}O_{29}^{2+}$: 2800.1506, LC-MS (ESI) RT= 1.76 min. m/z: 1400.50 $[M]^{2+}$, 934.17 $[M+H]^{3+}$, 700.92 $[M+2H]^{4+}$, 560.92 $[M+3H]^{5+}$, 467.58 $[M+4H]^{6+}$, 401.00 $[M+5H]^{7+}$; (positive) MALDI-TOF m/z found: 1941.167 $[M-H-2 \cdot Rhodamine]^{+}$.

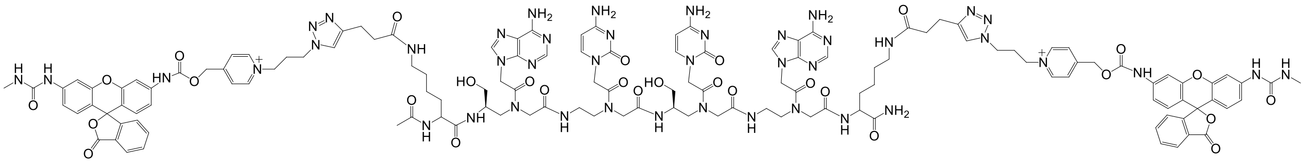

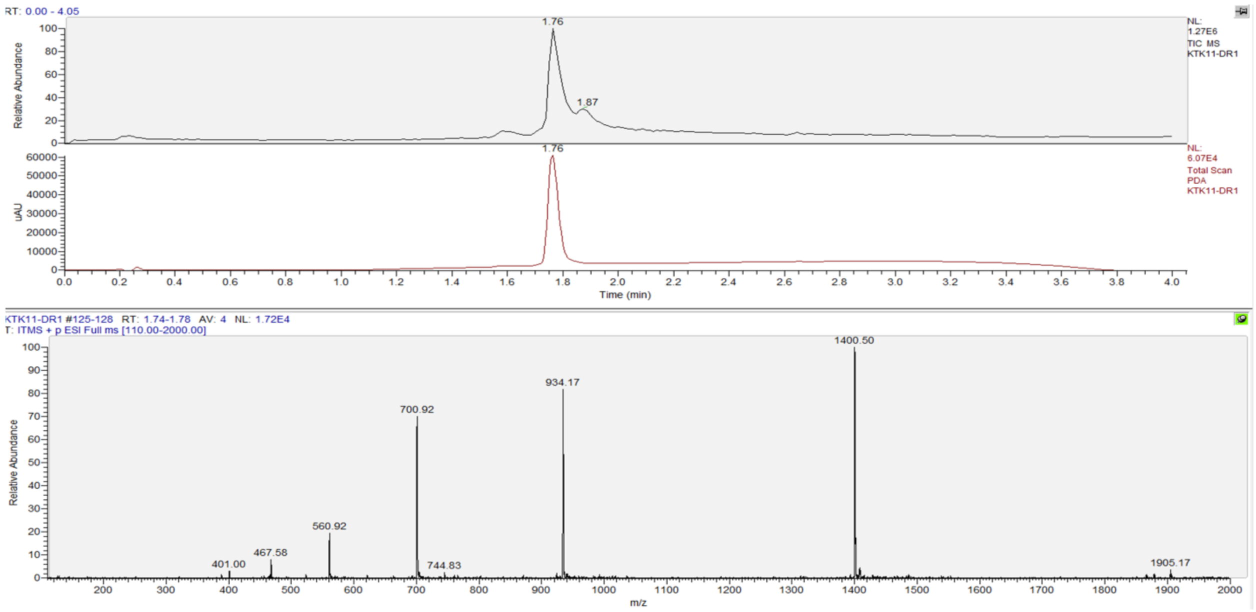

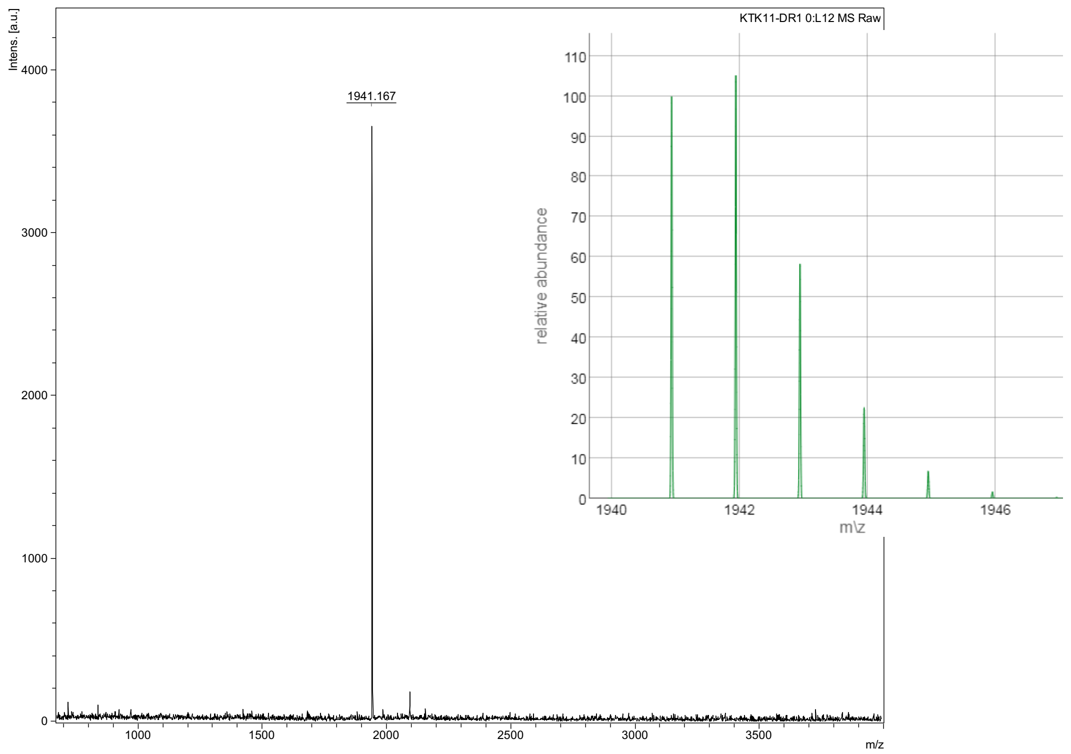

DC1-A (CouPy-Lys-AACA-Lys-PyCou); Exact Mass for $C_{107}H_{127}F_4N_{39}O_{22}^{2+}$: 2385.9954, LC-MS (ESI) RT= 1.55 min. m/z: 1193.42 $[M]^{2+}$, 796.00 $[M+H]^{3+}$, 597.33 $[M+2H]^{4+}$, 478.08 $[M+3H]^{5+}$; (positive) MALDI-TOF m/z found: 1965.186 $[M-H-2 \cdot Coumarin]^{+}$.

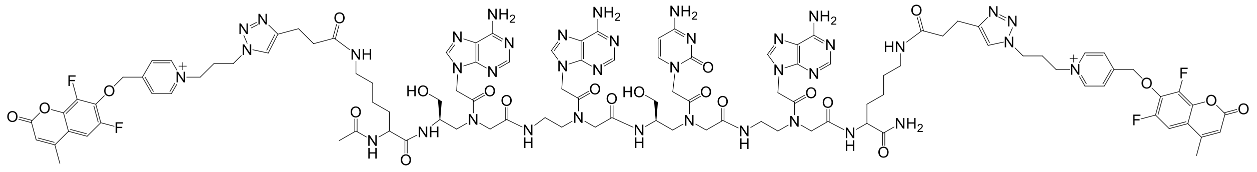

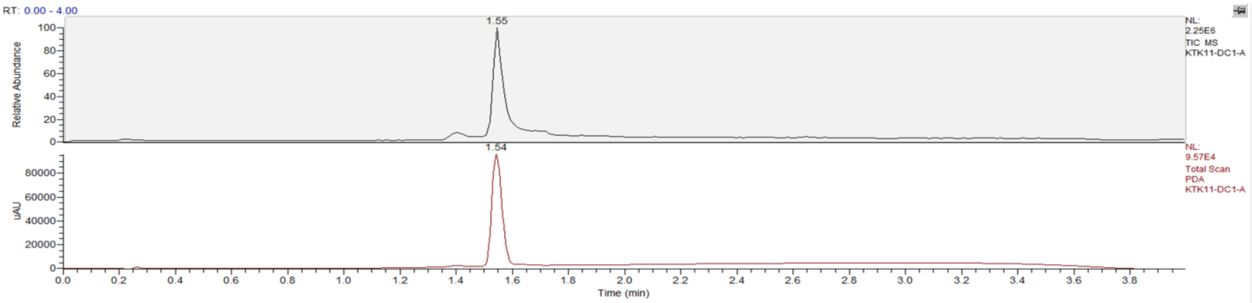

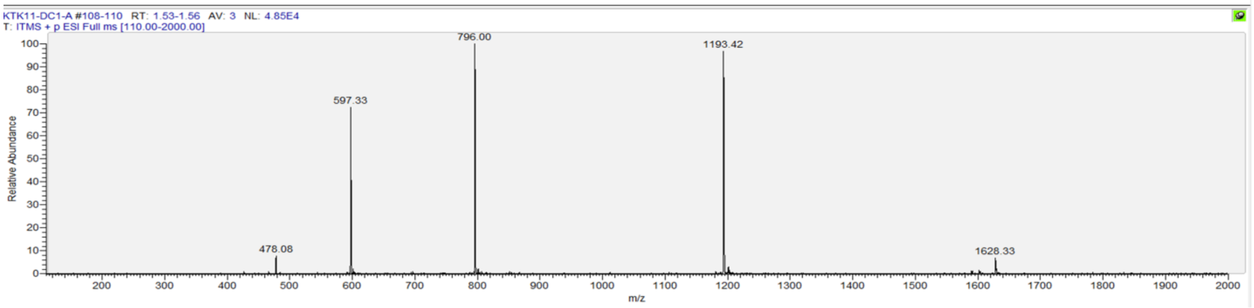

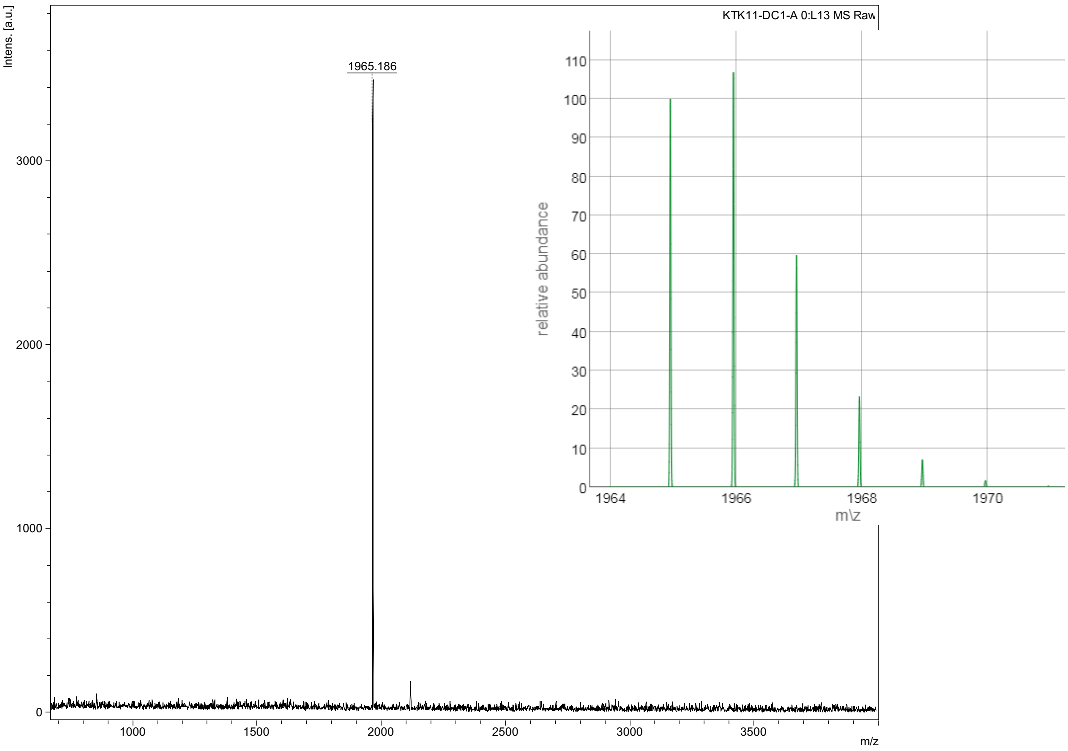

DC1-T (CouPy-Lys-<u>A</u>T<u>C</u>A-Lys-PyCou); Exact Mass for $C_{107}H_{128}F_4N_{36}O_{24}{}^{2+}$: 2376.9838, LC-MS (ESI) RT= 1.58 min. m/z: 1188.92 $[M]^{2+}$, 793.00 $[M+H]^{3+}$, 595.00 $[M+2H]^{4+}$, 476.17 $[M+3H]^{5+}$; (positive) MALDI-TOF m/z found: 1956.295 $[M-H-2 \cdot Coumarin]^{+}$.

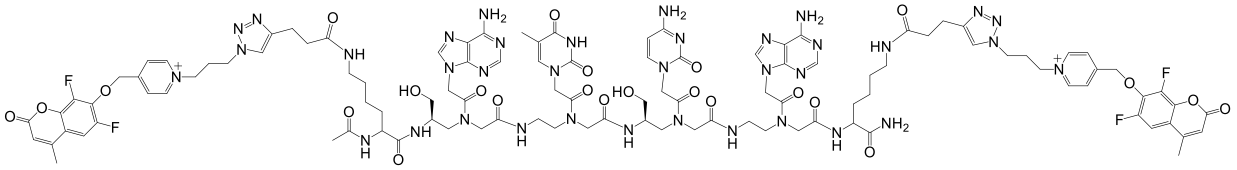

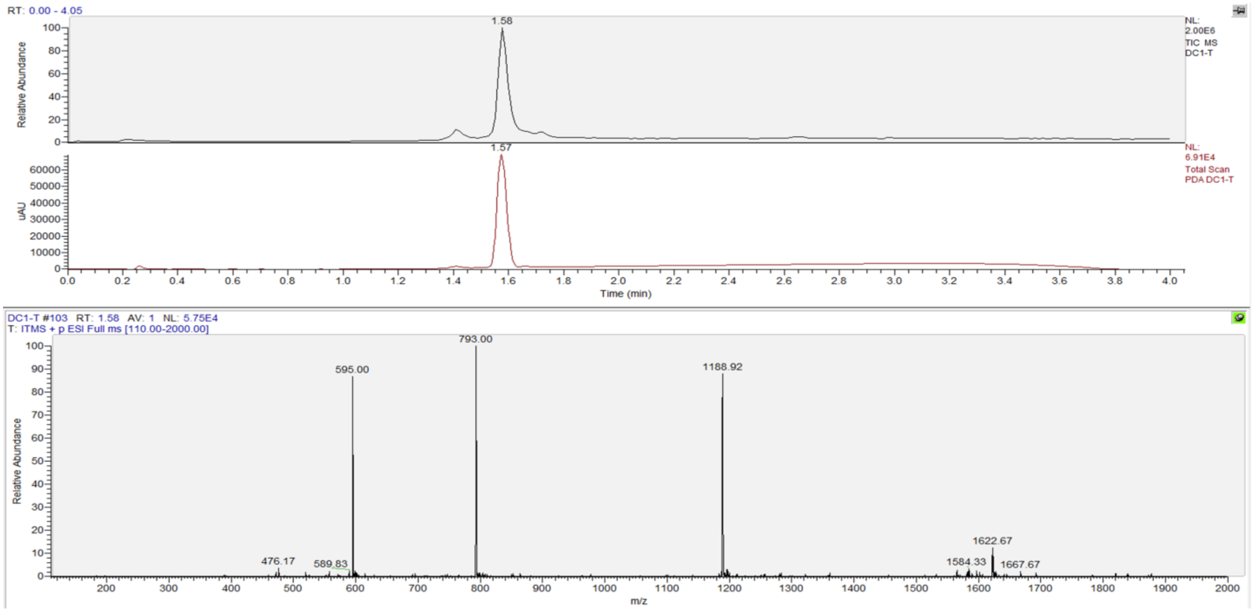

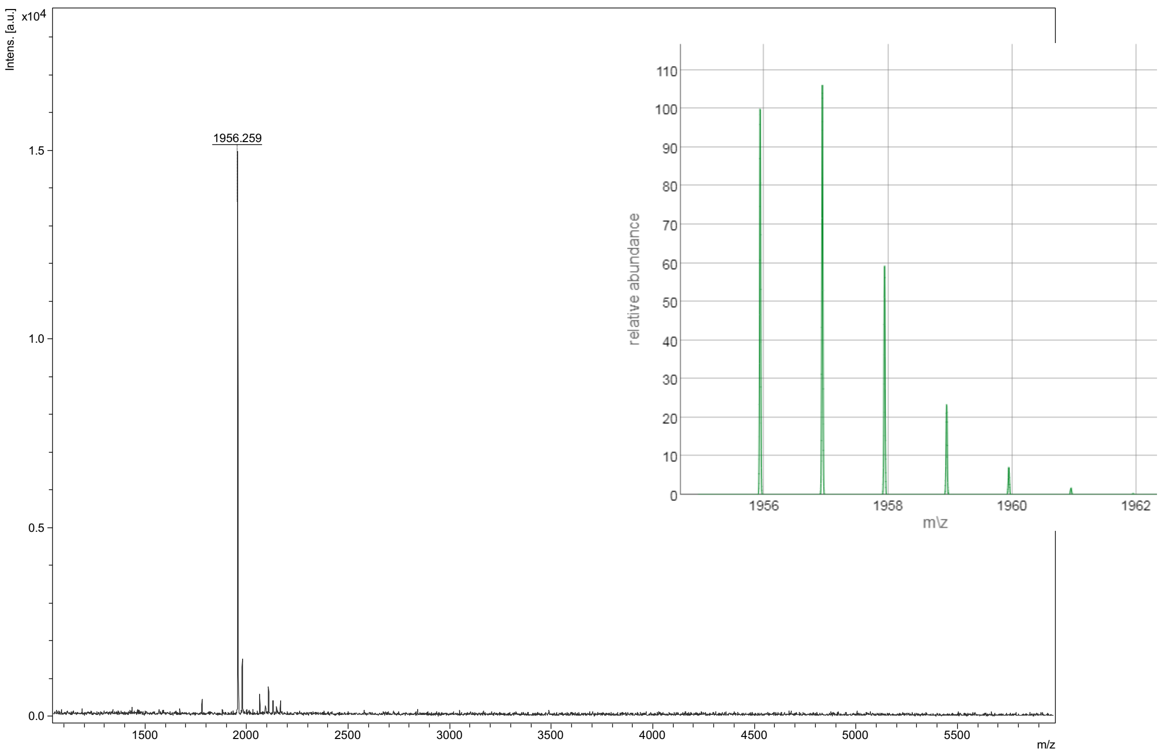

DR2 (RhoPy-Lys-A̲GT̲G-Lys-PyRho); Exact Mass for $C_{134}H_{150}N_{44}O_{31}{}^{2+}$: 2871.1514, LC-MS (ESI) RT= 1.82 min. m/z: 1436.00 $[M]^{2+}$, 957.92 $[M+H]^{3+}$, 718.67 $[M+2H]^{4+}$, 575.17 $[M+3H]^{5+}$, 479.50 $[M+4H]^{6+}$; (positive) MALDI-TOF m/z found: 2012.186 [M-H-2 • Rhodamine]$^{+}$.

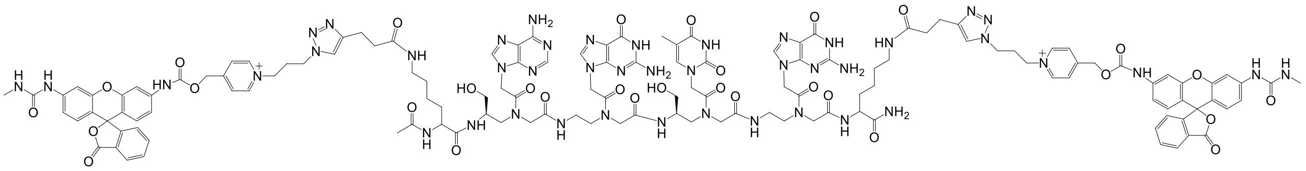

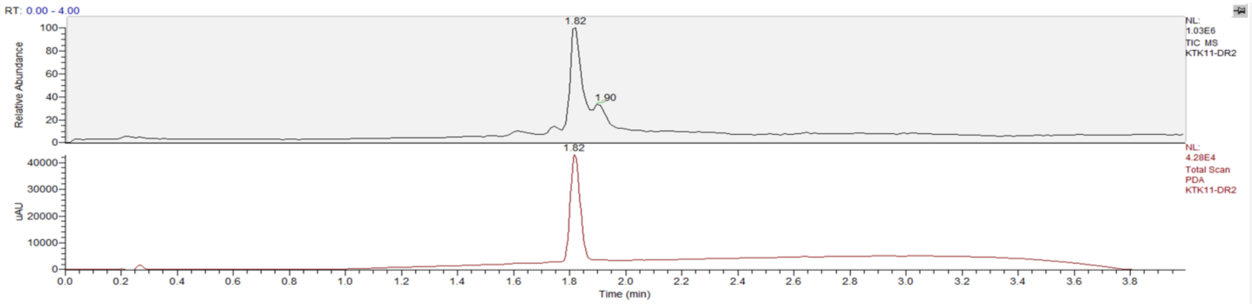

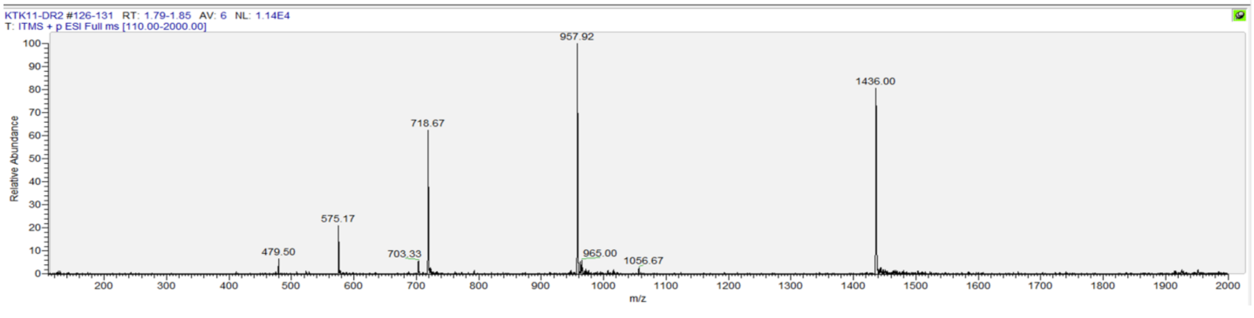

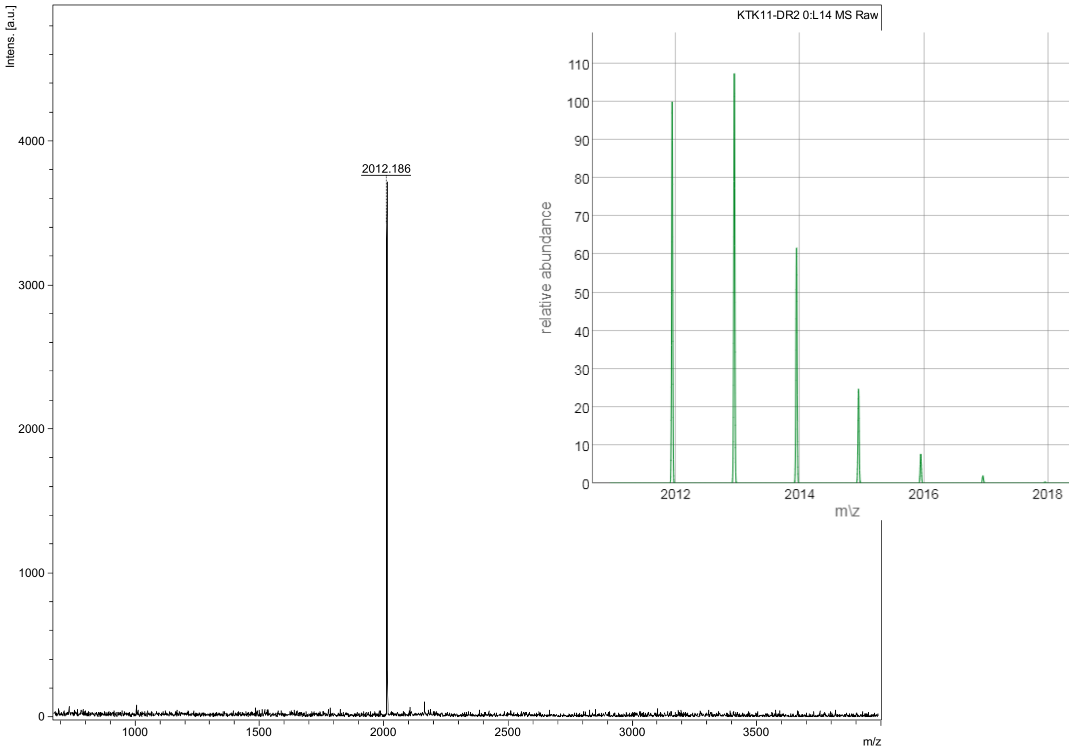

DC2-A (CouPy-Lys-AATG-Lys-PyCou); Exact Mass for $C_{108}H_{128}F_4N_{38}O_{24}^{2+}$: 2416.9900, LC-MS (ESI) RT= 1.58 min. m/z: 1208.92 $[M]^{2+}$, 806.33 $[M+H]^{3+}$, 605.00 $[M+2H]^{4+}$, 484.33 $[M+3H]^{5+}$; (positive) MALDI-TOF m/z found: 1996.333 [M-H-2 • Coumarin]$^{+}$.

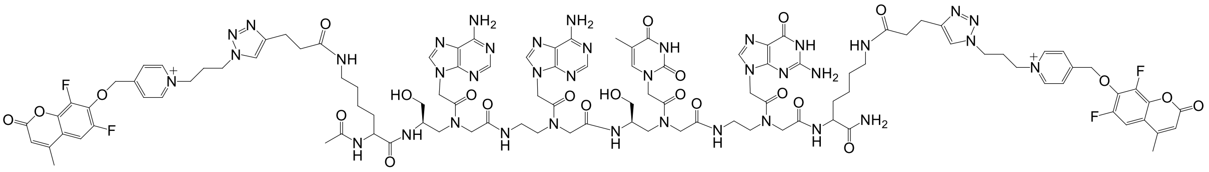

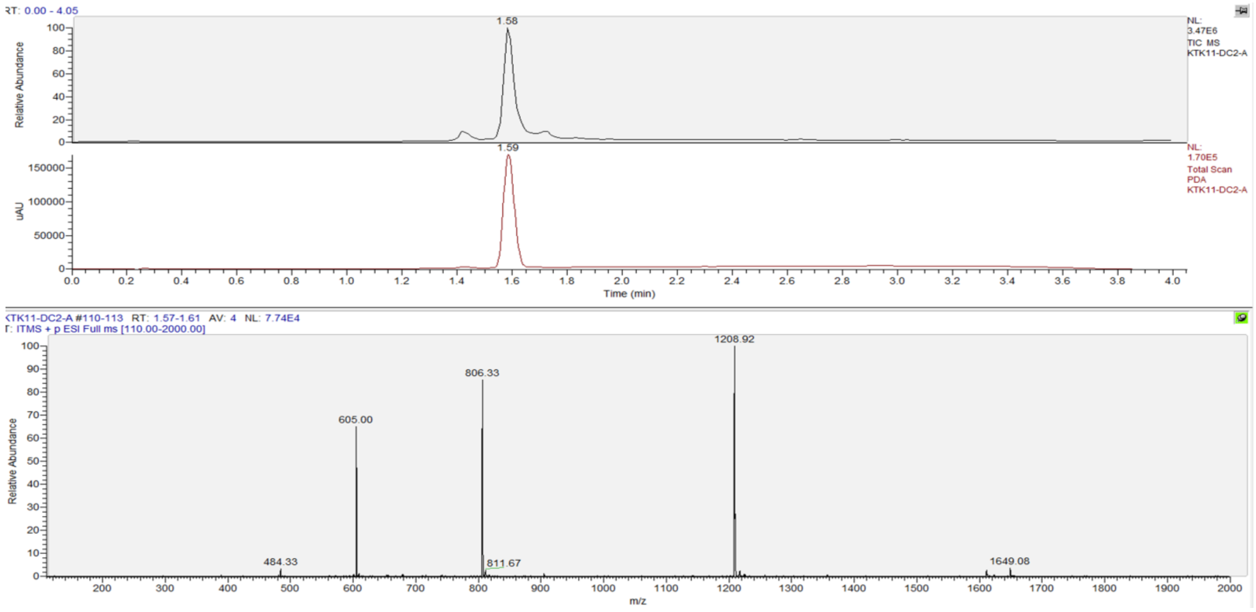

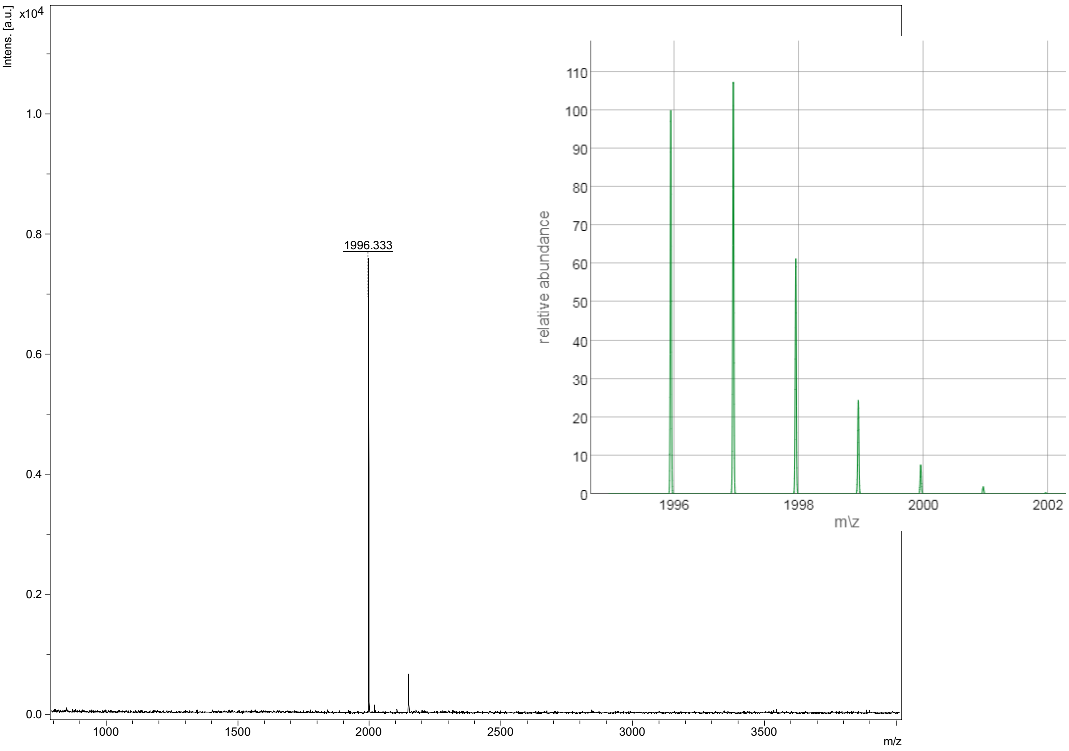

DC2-T (CouPy-Lys-ATTG-Lys-PyCou); Exact Mass for $C_{108}H_{129}F_4N_{35}O_{26}^{2+}$: 2407.9784, LC-MS (ESI) RT= 1.62 min. m/z: 1204.42 $[M]^{2+}$, 803.33 $[M+H]^{3+}$, 602.83 $[M+2H]^{4+}$; (positive) MALDI-TOF m/z found: 1987.276 $[M-H-2 \cdot Coumarin]^{+}$.

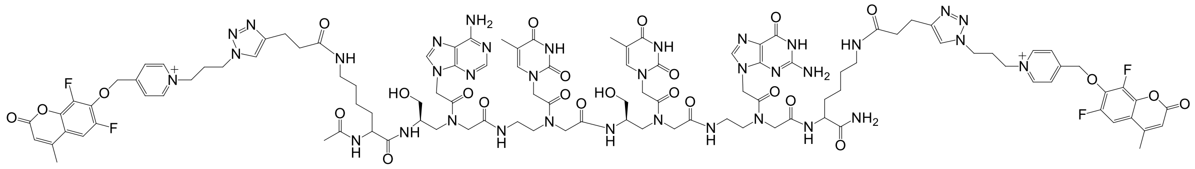

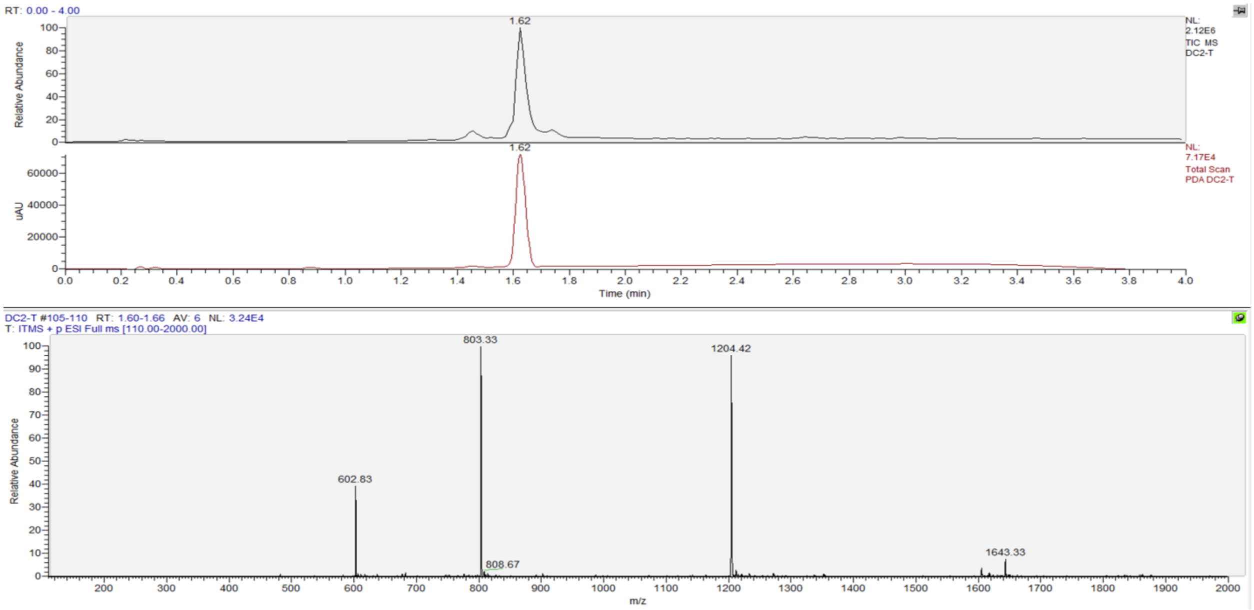

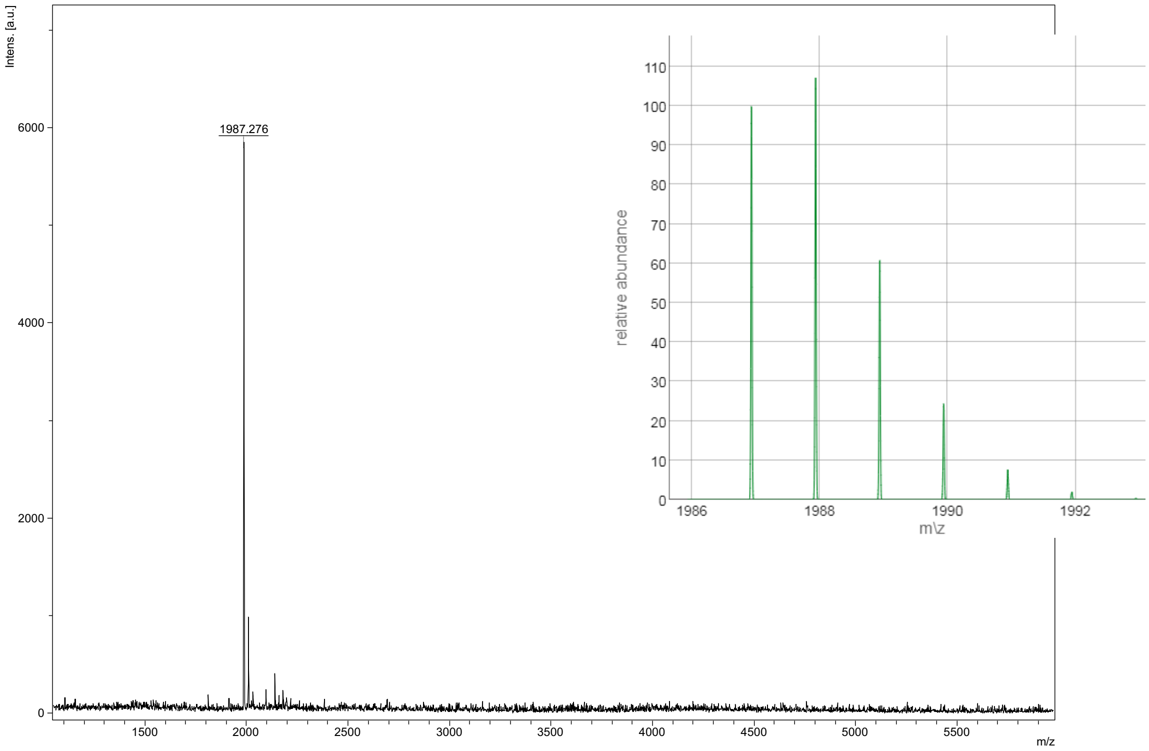

DR3 (RhoPy-Lys-<u>A</u>C<u>A</u>C-Lys-PyRho); Exact Mass for $C_{132}H_{149}N_{43}O_{29}^{2+}$: 2800.1506, LC-MS (ESI) RT= 1.76 min. m/z: 1400.50 $[M]^{2+}$, 934.08 $[M+H]^{3+}$, 700.92 $[M+2H]^{4+}$, 560.92 $[M+3H]^{5+}$, 467.58 $[M+4H]^{6+}$; (positive) MALDI-TOF m/z found: 1941.263 $[M-H-2 \bullet Rhodamine]^{+}$.

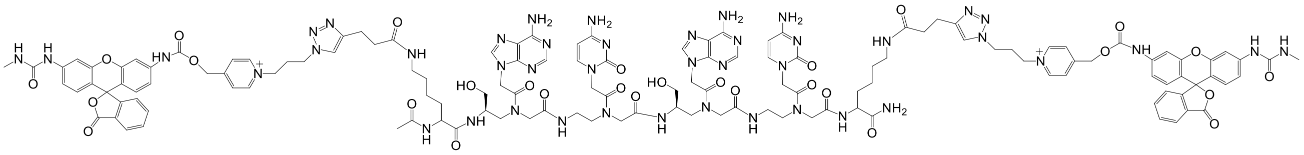

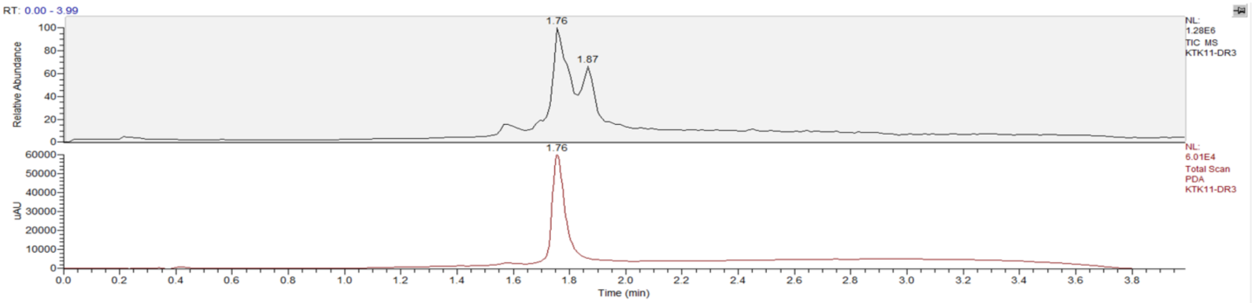

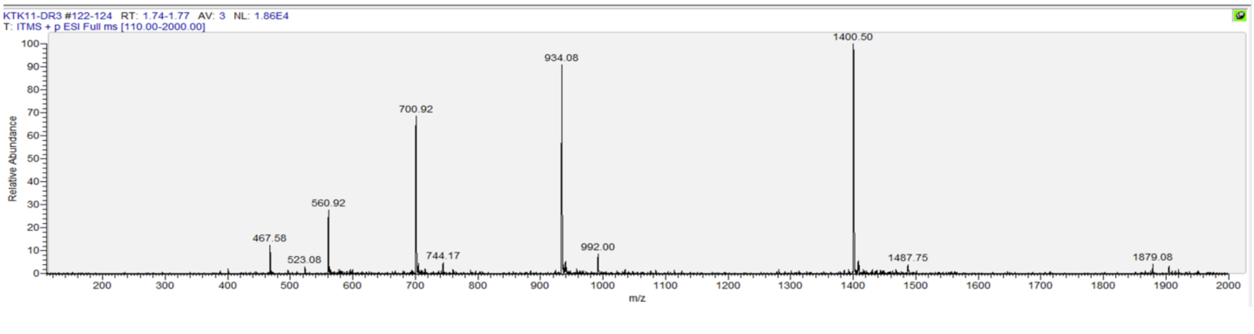

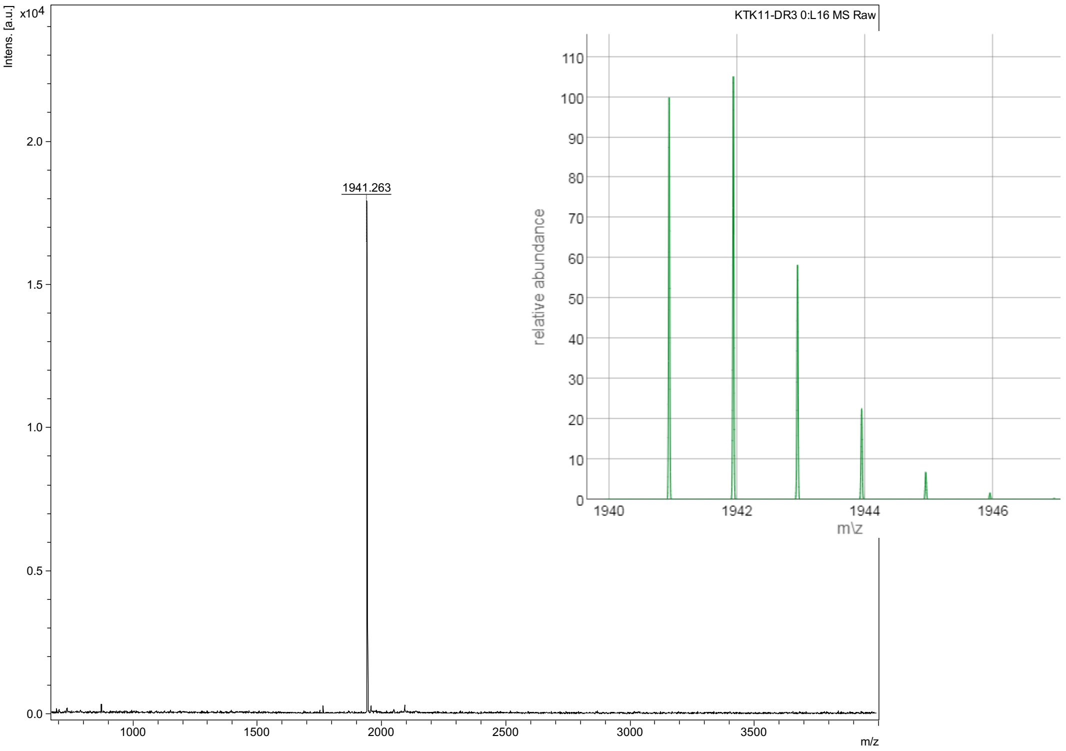

DC3-A (CouPy-Lys-<u>A</u>A<u>A</u>C-Lys-PyCou); Exact Mass for $C_{107}H_{127}F_4N_{39}O_{22}^{2+}$: 2385.9954, LC-MS (ESI) RT= 1.56 min. m/z: 1193.42 $[M]^{2+}$, 796.00 $[M+H]^{3+}$, 597.25 $[M+2H]^{4+}$, 478.00 $[M+3H]^{5+}$; (positive) MALDI-TOF m/z found: 1965.310 [M-H-2 • Coumarin]$^{+}$.

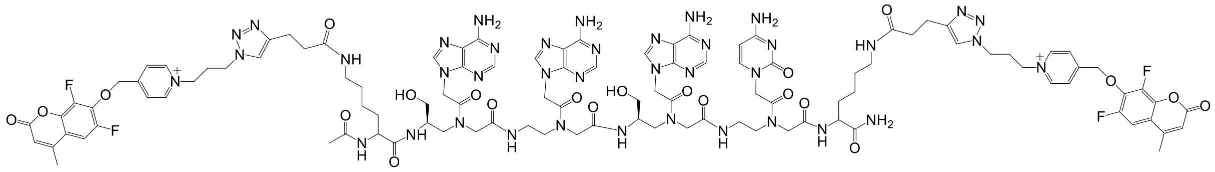

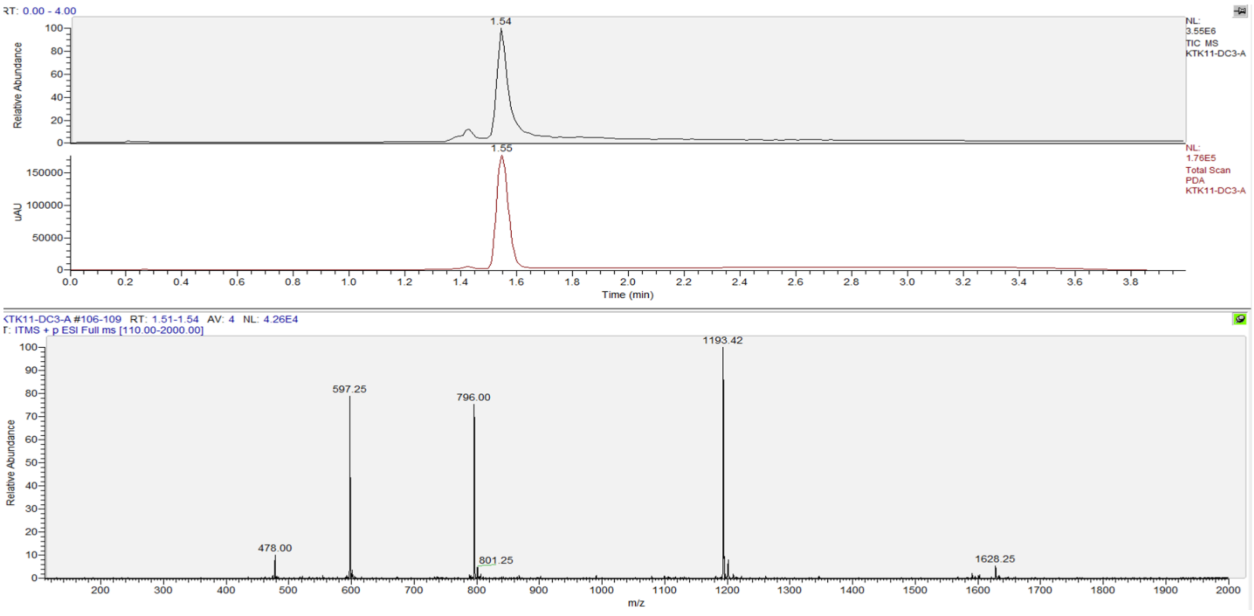

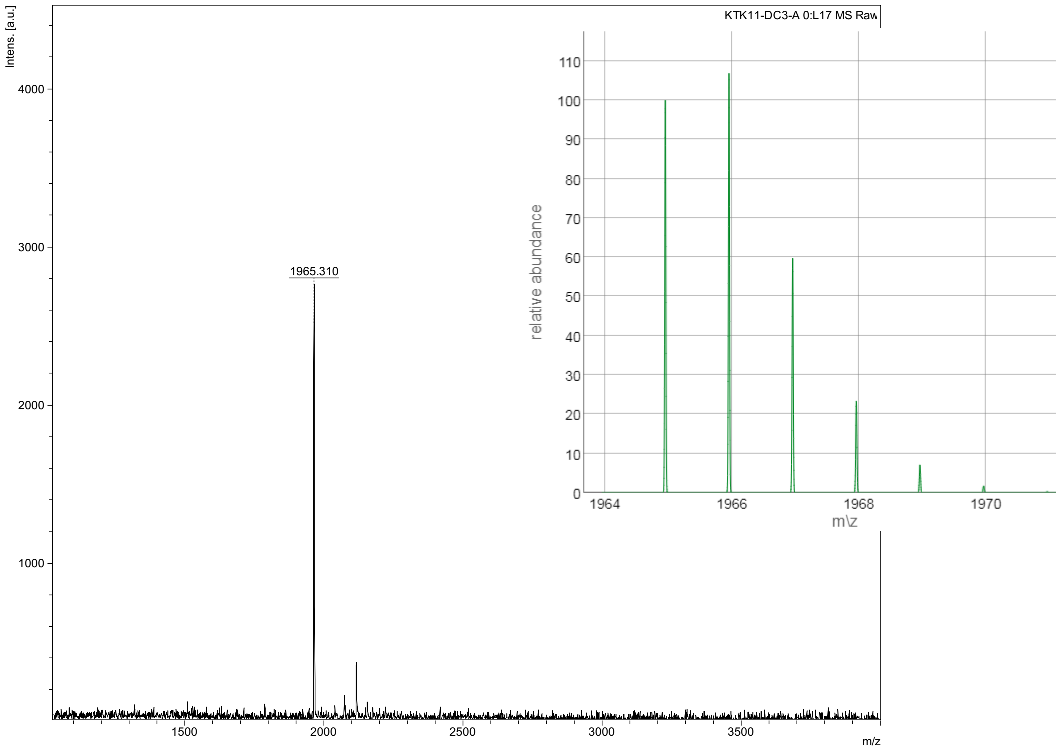

DC3-T (CouPy-Lys-<u>A</u>T<u>A</u>C-Lys-PyCou); Exact Mass for $C_{107}H_{128}F_4N_{36}O_{24}{}^{2+}$: 2376.9838, LC-MS (ESI) RT= 1.58 min. m/z: 1188.92 $[M]^{2+}$, 793.00 $[M+H]^{3+}$, 595.00 $[M+2H]^{4+}$, 476.17 $[M+3H]^{5+}$; (positive) MALDI-TOF m/z found: 1956.311 $[M-H-2 \bullet Coumarin]^{+}$.

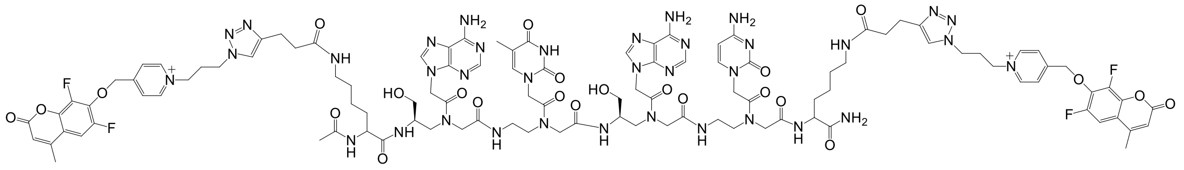

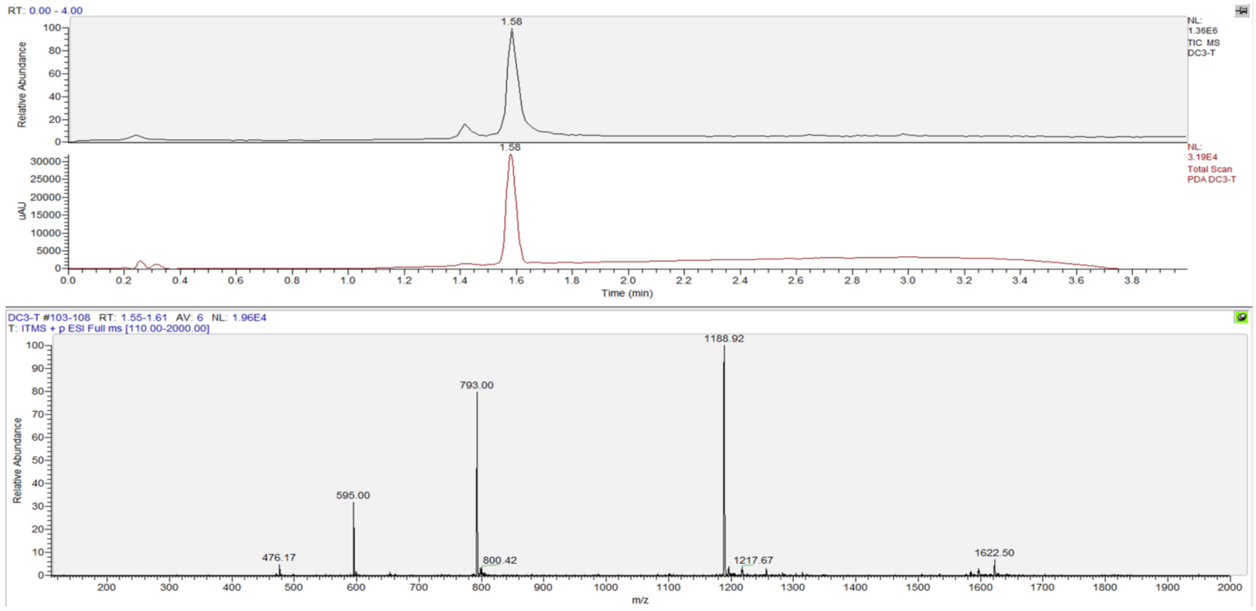

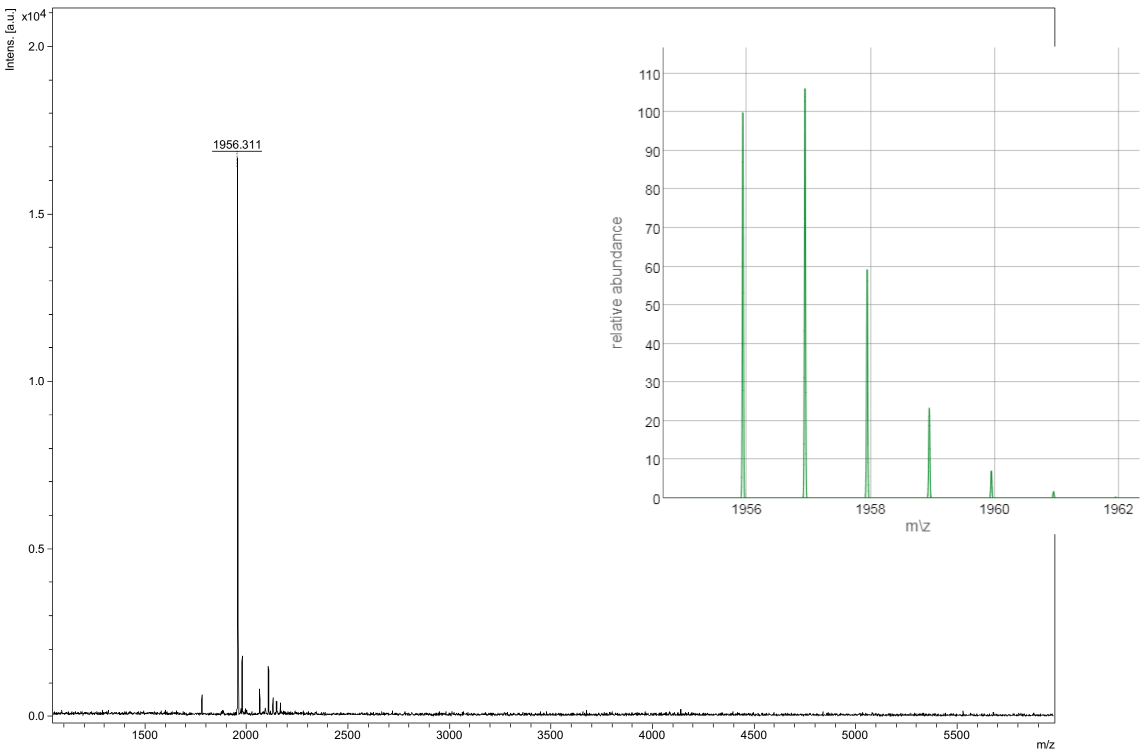

DC1-34A (CouPy-Lys-ACTA-Lys-PyCou); Exact Mass for $C_{107}H_{128}F_4N_{36}O_{24}^{2+}$: 2376.9838, LC-MS (ESI) RT= 1.57 min. m/z: 1188.92 $[M]^{2+}$, 793.00 $[M+H]^{3+}$, 595.00 $[M+2H]^{4+}$; (positive) MALDI-TOF m/z found: 1956.352 [M-H-2 • Coumarin]$^{+}$.

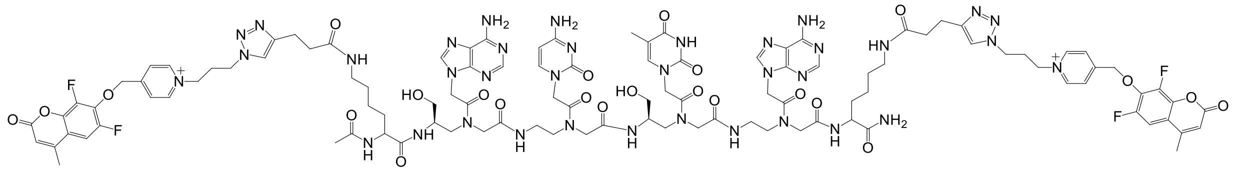

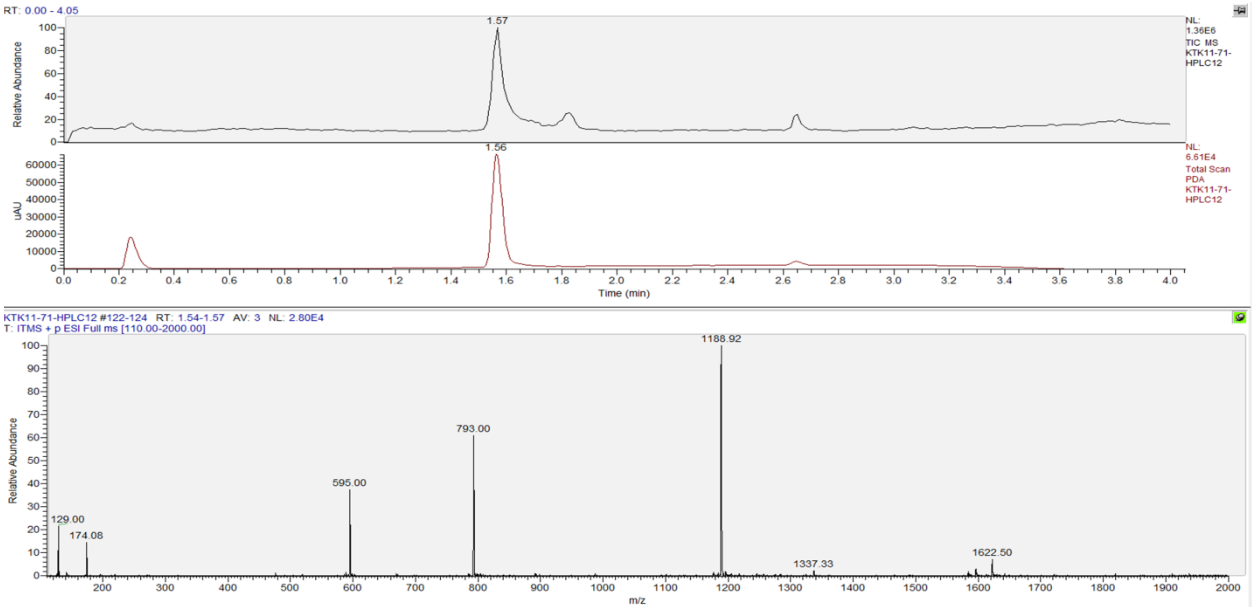

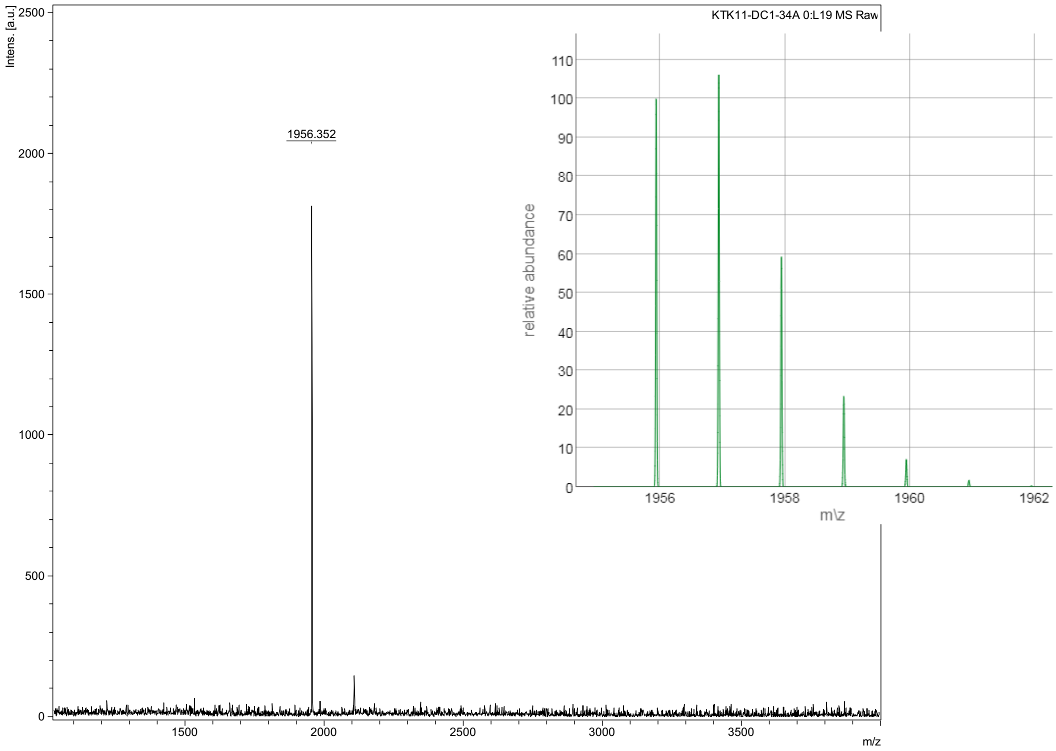

4. References

(1) Kim, K. T.; Angerani, S.; Chang, D.; Winssinger, N. *J. Am. Chem. Soc.* **2019**, *141*, 16288.

(2) Chang, D.; Lindberg, E.; Winssinger, N. *J. Am. Chem. Soc.* **2017**, *139*, 1444.

(3) Saarbach, J.; Lindberg, E.; Folliet, S.; Georgeon, S.; Hantschel, O.; Winssinger, N. *Chem. Sci.* **2017**, *8*, 5119.
